# Synthesis and insecticidal activity of diacylhydrazine derivatives containing a 3-bromo-1-(3-chloropyridin-2-yl)-1*H*-pyrazole scaffold

**DOI:** 10.1186/s13065-017-0279-z

**Published:** 2017-06-05

**Authors:** Yanyan Wang, Fangzhou Xu, Gang Yu, Jun Shi, Chuanhui Li, A’li Dai, Zhiqian Liu, Jiahong Xu, Fenghua Wang, Jian Wu

**Affiliations:** Key Laboratory of Green Pesticide and Agricultural Bioengineering, Ministry of Education, Research and Development Center for Fine Chemicals, Guizhou University, Guiyang, 550025 China

**Keywords:** Diacylhydrazine, 3-Bromo-1-(3-chloropyridin-2-yl)-1*H*-pyrazole, Synthesis and insecticidal activity

## Abstract

**Background:**

The diacylhydrazine derivatives have attracted considerable attention in recently years due to their simple structure, low toxicity, and high insecticidal selectivity. As well as 3-bromo-1-(3-chloropyridin-2-yl)-1*H*-pyrazole is an important scaffold in many insecticidal molecules. In an effort to discover new molecules with good insecticidal activity, a series of diacylhydrazine derivatives containing a 3-bromo-1-(3-chloropyridin-2-yl)-1*H*-pyrazole scaffold was synthesized and bio-assayed.

**Results:**

Bioassays demonstrated that some of the title compounds exhibited favorable insecticidal activities against *Helicoverpa armigera* and *Plutella xylostella*. The insecticidal activity of compounds **10g**, **10h**, and **10w** against *H. armigera* were 70.8, 87.5, and 79.2%, respectively. Compounds **10c**, **10e**, **10g**, **10h**, **10i**, **10j** and **10w** showed good larvicidal activity against *P. xylostella*. In particular, the LC_50_ values of compounds **10g**, **10h**, and **10w** were 27.49, 23.67, and 28.90 mg L^−1^, respectively.

**Conclusions:**

A series of diacylhydrazine derivatives containing a 3-bromo-1-(3-chloropyridin-2-yl)-1*H*-pyrazole scaffold was synthesized and bio-assayed. The results of insecticidal tests revealed that the synthesized diacylhydrazine derivatives possessed weak to good insecticidal activities against *H. armigera* and *P. xylostella*. Compounds **10g**, **10h**, and **10x** showed much higher insecticidal activity than tebufenozide, and exhibited considerable prospects for further optimization. Primary structure–activity relationship revealed that phenyl, 4-fluoro phenyl and four fluorophenyl showed positive influence on their insecticidal activities, and introduction of a heterocyclic ring (pyridine and pyrazole) showed negative impacts on their insecticidal effects.

**Electronic supplementary material:**

The online version of this article (doi:10.1186/s13065-017-0279-z) contains supplementary material, which is available to authorized users.

## Background

**Fig. 1:**
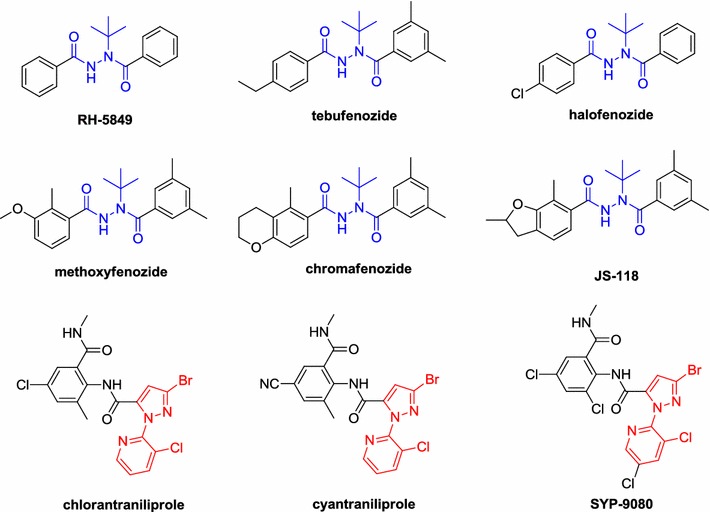
The structures of commercial insecticides containing the substructures of diacylhydrazine and 3-bromo-1-(3-chloropyridin-2-yl)-1*H*-pyrazole

Diacylhydrazines are important of nonsteroidal ecdysone agonists inducing agent against lepidopteron, which show excellent insecticidal activity by inducing precocious molting. The earliest insecticidal diacylhydrazine was developed by Rohm and Haas Company and named RH-5849, which was also investigated for their mode of action [1, 2]. Tebufenozide, the first commercialized diacylhydrazine as a specific insecticide for lepidopteron, was applied widely in many countries [3]. And then, several diacylhydrazine insecticides such as halofenozide, methoxyfenozide, chromafenozide, and JS-118 (Fig. 1), were also commercialized gradually [4–7]. Recently, diacylhydrazine derivatives have attracted considerable attention due to their simple structure, low toxicity, and high insecticidal selectivity, and a large number of insecticidal molecules were discovered [8–23].

3-Bromo-1-(3-chloropyridin-2-yl)-1*H*-pyrazole is an important scaffold and appear in several commercial insecticides structures, such as chlorantraniliprole [24], cyantraniliprole [25], and SYP-9080 (Fig. 1) [26]. In recent years, a large number of insecticidal molecules containing a 3-bromo-1-(3-chloropyridin-2-yl)-1*H*-pyrazole were reported [27–30]. Among which, some diacylhydrazines containing 3-bromo-1-(3-chloropyridin-2-yl)-1*H*-pyrazole scaffold were also reported [11, 31], such as *N*-(2-(2-(3-bromo-1-(3-chloropyridin-2-yl)-1*H*-pyrazole-5-carbonyl)-2-(*tert*-butyl) hydrazinecarbonyl)-5-chloro-3-methylphenyl) acetamide show 100% larvicidal activity against *Mythimna separate* at 100 mg L^−1^. And in our previous works [15, 32–35], a series of diacylhydrazine derivatives containing 3-bromo-1-(3-chloropyridin-2-yl)-1*H*-pyrazole was also been confirmed to show good insecticidal activities.

**Fig. 2:**
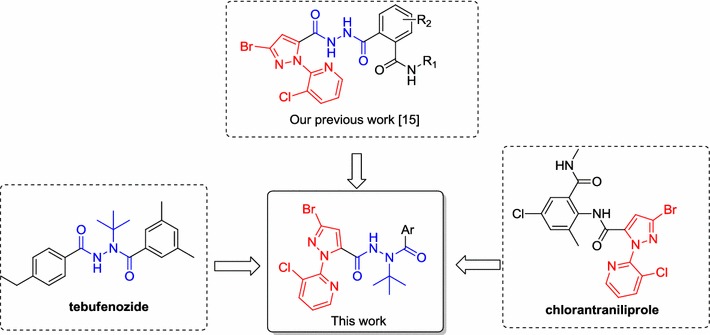
The design of title compounds

Encouraged by descriptions above and as a continuation of insecticidal molecules with 3-bromo-1-(3-chloropyridin-2-yl)-1*H*-pyrazole, we herein sought to retain the substructure of 3-bromo-1-(3-chloropyridin-2-yl)-1*H*-pyrazole and *tert*-butyl diacylhydrazine, and introducing different substituted aryls (Fig. 2). A series of novel diacylhydrazine derivatives was designed and synthesized. Structures of the synthesized compounds were characterized by ^1^H NMR, ^13^C NMR, and HR-MS. Results of bioassays indicated that most synthesized compounds exhibit good insecticidal activities against *P. xylostella*. In particular, the compounds **10g**, **10h**, and **10x** exhibited excellent insecticidal activities, with LC_50_ values of 27.49, 23.67, and 28.90 mg L^−1^, respectively. These compounds showed slightly higher insecticidal activity than commercial tebufenozide (LC_50_ = 37.77 mg L^−1^).

## Results and discussion

### Chemistry

**Scheme 1:**
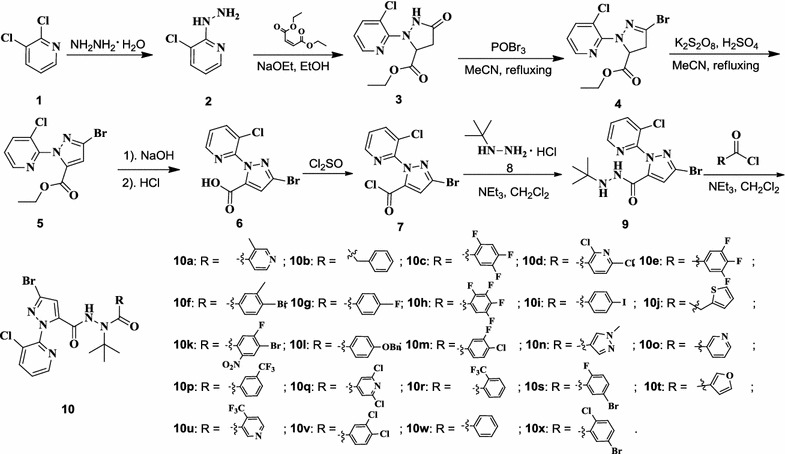
Synthetic route for compounds **10a**–**10x**

The synthesis of the 3-bromo-1-(3-chloropyridin-2-yl)-1*H*-pyrazole-5-carbohydrazide derivatives are depicted in Scheme 1. Firstly, the key intermediate 3-bromo-1-(3-chloropyridin-2-yl)-1*H*-pyrazole-5-carboxylic acid (**5**) was obtained in good yield via reactions of hydrazinolysis, cyclization, bromination, oxydehydrogenation, and acidolysis by employing 2,3-dichloropyridine (**1**), hydrazine hydrate and diethyl maleate as starting materials [24, 33, 34]. Then compound **5** was allowed to further react with thionyl chloride under reflux to afford 3-bromo-1-(3-chloropyridin-2-yl)-1*H*-pyrazole-5-carbonyl chloride (**7**) [35]. Subsequent treatment of intermediate **7**, with *tert*-butyl hydrazine hydrochloride (**8**) in the presence of triethylamine in trichloromethane at ambient temperature afforded 3-bromo-*N*′-(*tert*-butyl)-1-(3-chloropyridin-2-yl)-1*H*-pyrazole-5-carbohydrazide (**9**) in 80% yield. Finally, the title compounds (**10a**–**10x)** were conveniently obtained in an >70% yield by treating of intermediate **9** with the corresponding acyl chloride in the presence of triethylamine in acetone or acetonitrile.

Structures of the title compounds (**10a**–**10x**) were established on basis of their spectroscopic data. In the ^1^H NMR spectra, the N–H proton appeared as a broad singlet near δ 11.10 ppm. The proton at position 5 of pyridine appeared as a doublet of doublets near *δ* 8.45 due to the coupling coefficients from the protons at 3 and 4 positions of the pyridine ring; the coupling constants were ^3^ *J* = 4.7 Hz and ^4^ *J* = 1.5 Hz respectively. As well as the protons at positions 3 and 4 showed as doublet of doublets near *δ* 8.2 and 7.7 ppm, respectively, because of the coupling coefficients from both 5 positions and the each other from 4 and 3 positions of the pyridine ring, respectively. 4-pyrazole-H exhibited a singlet near *δ* 6.90 ppm. The rest of the aromatic protons appeared range from 7.0 to 8.0 ppm, the nine protons (–CH_3_)_3_ appeared as a singlet near *δ* 1.45 ppm; In ^13^C NMR spectra for the fluorine contained compounds, the carbons were split into multiplet due to the coupling coefficients from “F”, take compound **10m** as example, the carbon near “F” resonance frequency is near *δ*_*C*_ 158.27 ppm as a doublet and with the coupling constant (^1^ *J*_*C*-*F*_) was 249.5 Hz; and the carbons at ortho-position of F were also split into doublets with coupling constant (^2^ *J*_*C*-*F*_) ranged from 18.1 Hz to 21.4 Hz. The properties, ^1^H NMR, ^13^C NMR, ^19^F NMR, and HR-MS data of the synthesized compounds **10a** to **10x** are summarized in more detail in the “Experimental section”.

### Insecticidal activity

The insecticidal activities of the synthesized compounds against both *Helicoverpa armigera* and *Plutella xylostella* were evaluated using procedures reported previously [17, 33–36] and summarized in Tables 1 and 2, respectively. Commercial tebufenozide, chlorantraniliprole, and chlorpyrifos were used as positive controls.

**Table 1:** Larvicidal activity of compounds **10a**–**10s** against *Helicoverpa armigera*

Compounds	Larvicidal activity (%) at different concentrations (mg L^−1^)				
	500	200	100	50	25
**10a**	45.8	22.2	0.0	/	/
**10b**	16.7	0.0	/	/	/
**10c**	62.5	44.4	21.4	6.7	/
**10d**	58.3	38.9	14.3	/	/
**10e**	62.5	44.4	21.4	/	/
**10f**	58.3	38.9	14.3	/	/
**10g**	70.8	55.6	35.7	/	/
**10h**	87.5	77.8	64.3	43.3	16.7
**10i**	54.2	33.3	7.1	/	/
**10j**	66.7	40.0	28.6	13.3	/
**10k**	33.3	5.6	0.0	/	/
**10l**	58.3	38.9	14.3	/	/
**10m**	37.5	11.1	0.0	/	/
**10n**	41.7	16.7	0.0	/	/
**10o**	63.3	46.7	26.7	6.7	/
**10p**	54.2	33.3	7.1	/	/
**10q**	58.3	38.9	14.3	/	/
**10r**	30.0	0.0	/	/	/
**10s**	41.7	16.7	0.0	/	/
**10t**	33.3	5.6	0.0	/	/
**10u**	0.0	/	/	/	/
**10v**	54.2	33.3	7.0	/	/
**10w**	*79.2*	60.0	53.3	23.3	6.7
**10x**	41.7	16.7	0.0	/	/
Tebufenozide	100	93.3	70.0	50	40.0
Chlorpyrifos	100	100	100	90	83
Chlorantraniliprole	100	100	100	100	100

**Table 2:** Larvicidal activity of compounds (**10a**–**10s**) against *Plutella xylostella*

Compounds	Larvicidal activity (%) at different concentrations (mg L^−1^)				
	500	200	100	50	25
**10a**	70.0	46.7	21	/	/
**10b**	33.3	16.7	0.0	/	/
**10c**	86.7	56.7	30.0	16.7	/
**10d**	76.7	53.3	23.6	/	/
**10e**	90.0	73.3	53.3	36.7	16.7
**10f**	66.7	53.5	30.2	/	/
**10g**	100	96.7	80.0	66.7	50.0
**10h**	100	100	93.3	76.7	53.3
**10i**	90.0	63.3	43.3	33.3	16.7
**10j**	96.7	83.3	53.3	36.7	23.3
**10k**	56.7	23.3	3.3	/	/
**10l**	73.3	53.3	16.7	6.7	/
**10m**	63.3	33.3	16.7	/	/
**10n**	56.7	33.3	13.1	/	/
**10o**	80.0	63.3	33.7	16.7	/
**10p**	76.7	53.3	13.0	/	/
**10q**	73.3	49.0	20.0	/	/
**10e**	43.3	23.3	13.3	/	/
**10s**	66.7	33.3	16.7	/	/
**10t**	43.3	23.3	6.7	/	/
**10u**	6.7	0.0	/	/	/
**10v**	80.0	66.7	23.3	/	/
**10w**	100	100	86.7	70.0	46.7
**10x**	66.7	33.3	13.3	/	/
Tebufenozide	100	96.7	80.0	56.7	26.7
Chlorpyrifos	100	100	100	90	83
Chlorantraniliprole	100	100	100	100	100

The results listed in Table 1 indicated that the synthesized compounds displayed weak to good larvicidal activity against *Helicoverpa armigera* at the test concentration. For example, the larvicidal activity of compounds **10c** to **10j**, **10l**, **10o**–**10q**, **10v**, and **10w** showed >50% mortality on *H. armigera* at 500 mg L^−1^, and the larvicidal activity of **10g**, **10h**, and **10w** were 70.8, 87.5, and 79.2%, respectively, whereas the concentration was 100 mg L^−1^, the mortalities of *H. armigera* for compounds **10h** and **10w** were still >50%.

As shown in Table 2, the synthesized compounds shown larvicidal activity against *Plutella xylostella*, with mortality range from 6.7 to 100%. And it can be seen that most of the synthesized compounds show over 60% activity at 500 mg L^−1^, and compounds **10e**, **10g** to **10j** and **10w** displayed >90% activities. In particular, compounds **10g**, **10h** and **10w** showed good larvicidal activity, both **10h** and **10w** showed 100% activities against *Plutella xylostella* at 200 mg L^−1^, and the activity of compound **10g** was up to 96.7%. When the concentration was 50 mg L^−1^, the activities of compounds **10g**, **10h** and **10w** were 66.7, 76.7 and 70% at 50 mg L^−1^, respectively, whereas these three compounds showed moderate activity at 25 mg L^−1^.

The median lethal concentrations (LC_50_) of compounds **10c**, **10e**, **10g**, **10h**, **10i**, **10j** and **10w** were further determined. For comparison, the LC_50_ value of tebufenozide (a commonly used insecticide) were also evaluated. The results are given in Table 3. The LC_50_ values of compounds **10e**, **10g**, **10h**, **10j** and **10w** were less than 100 mg L^−1^ (Table 3). In particular, the compounds **10g**, **10h**, and **10w** exhibited excellent insecticidal activities, with LC_50_ values of 27.49, 23.67, and 28.90 mg L^−1^, respectively. These compounds showed slightly higher insecticidal activity than commercial tebufenozide (LC_50_ = 37.77 mg L^−1^). As revealed by data in Tables 1 and 2, the insecticidal activity of the title compound was effected by R group. When R was a benzene ring (**10w**), the compound showed excellent insecticidal activity (compare with tebufenozide), and the activity could be slightly enhanced by introduction of a fluorine at 4 position of benzene (compound **10g**) or four fluorines on benzene (**10h**). However, the activity decreased when benzene was substituted by tri-fluorine at 3, 4, 5 positions, as well as decreased by introducing other substituents, such as nitro, 2-trifluoromethyl, 3-trifluoromethyl, 3,4-di-chloro, and 4-iodine. In addition, when R was a heterocyclic ring (i.e., pyridine, pyrazole, furan), the corresponding compounds showed much weaker activities than the compounds with a benzene ring. Moreover, a compound containing the benzyl show no larvicidal activity. But interestingly, a compound containing the 2-thiophen-2-yl (**10j**) was found to show good insecticidal activity.

**Table 3:** LC_50_ values for insecticidal activity against *Plutella xylostella*

Comp.	y = a + bx	r	LC_50_ (mg L^−1^)
**10c**	Y = 0.632181 + 1.993794x	0.99	155.13
**10e**	Y = 1.699094 + 1.701997x	0.99	86.98
**10g**	Y = 2.248458 + 1.91187x	0.97	27.49
**10h**	Y = 1.687545 + 2.410609x	0.99	23.67
**10i**	Y = 1.661246 + 1.658921x	0.98	102.95
**10j**	Y = 1.699094 + 1.701997x	0.99	69.07
**10w**	Y = 1.85713 + 2.15129x	0.99	28.90
Tebufenozide	Y = 1.429139 + 2.2641 x	0.99	37.77

## Experimental section

### Materials and instruments

All aromatic acids were purchased from Accela ChemBio Co., Ltd (Shanghai, China). Melting points were determined using a XT-4 binocular microscope (Beijing Tech Instrument Co., China) and left uncorrected. The NMR spectra was recorded on a AVANCE III HD 400M NMR (Bruker corporation, Switzerland) or JEOL ECX 500 NMR spectrometer (JEOL Ltd., Japan) operating at room temperature using DMSO as solvent. HR-MS was recorded on an Orbitrap LC–MS instrument (Q-Exative, Thermo Scientific™, American). The course of the reactions was monitored by TLC; analytical TLC was performed on silica gel GF254. All reagents were of analytical grade or chemically pure. All anhydrous solvents were dried and purified according to standard techniques just before use.

### Synthetic procedures

#### General procedure for intermediates (**2**–**6**)

Intermediates **2**–**6** were prepared by following the known procedures, [24, 33, 34] and the acyl chloride (**7**) was synthesized according to reported method [35]. The detailed synthetic procedures and physical properties for these intermediates can be found in Additional file 1.

#### Synthesis of intermediate (**9**)

To a well-stirred suspension of *tert*-butyl hydrazine hydrochloride **8** in dichloromethane, two equivalents of triethylamine was added, the resulted mixture was stirred at room temperature for 10 min, then the solution of acyl chloride **7** in dichloromethane was then added dropwise. After stirring and refluxing for 2 h, dichloromethane was removed in vacuo. The mixture was washed with saturated sodium bicarbonate solution. The solution was filtered to obtain a crude product, which was recrystallized with ethanol to obtain the 3-bromo-*N′*-(*tert*-butyl)-1-(3-chloropyridin-2-yl)-1*H*-pyrazole-5-carbohydrazide (**9**). Brown solid, yield, 80%, ^1^H NMR (500 MHz, DMSO-D6) δ 10.08 (brs, 1H, N–H), 8.47 (d, *J* = 4.6 Hz, 1H, pyridine-H), 8.15 (d, *J* = 8.0 Hz, 1H, pyridine-H), 7.58 (dd, *J* = 8.0, 4.7 Hz, 1H, pyridine-H), 7.25 (s, 1H, pyrazole-H), 4.78 (brs, 1H, N–H), 0.96 (s, 9H, 3 CH_3_).

#### General procedure for the preparation of title compounds (**10a**–**10y**)

Different fresh acyl chloride (1 mmol) were added to a well-stirred solution of **9** (1 mmol) in chloroform (5 mL) in present of triethylamine. The resulting mixture was stirred for 50 min at ambient temperature to afford a white solid, and then filtered and recrystallized from ethanol in good yield.

#### *N*′-(3-Bromo-1-(3-chloropyridin-2-yl)-1*H*-pyrazole-5-carbonyl)-*N*-(*tert*-butyl)-3-methylisonicotinohydrazide **(10a)**

White solid. M.p: 286–287 °C; yield: 78%; ^1^H NMR (400 MHz, DMSO) δ 10.98 (s, 1H, N–H), 8.50 (dd, ^3^ *J* = 4.7 Hz, ^4^ *J* = 1.5 Hz, 1H, pyridine-H), 8.44 (s, 1H, pyridine-H), 8.35 (d, ^3^ *J* = 4.9 Hz, 1H, Ar–H), 8.23 (dd, ^3^ *J* = 8.1 Hz, ^4^ *J* = 1.5 Hz, 1H, pyridine-H), 7.67 (dd, ^3^ *J* = 8.1 Hz, ^4^ *J* = 4.7 Hz, 1H, pyridine-H), 6.97 (s, 1H, pyrazole-H), 6.69 (s, 1H, pyridine-H), 2.17 (s, 3H, –CH_3_), 1.45 (s, 9H, 3CH_3_); ^13^C NMR (100 MHz, DMSO) δ 170.00, 157.50, 151.54, 147.99, 147.70, 147.02, 144.56, 140.09, 137.31, 128.01, 127.45, 127.25, 119.22, 110.78, 61.57, 27.66, 15.68. HR-MS (ESI^+^) *m/z* Calcd for C_20_H_20_BrClN_6_O_2_ [M + H]^+^ 491.05978; found 491.05980.

#### 3-Bromo-*N*′-(*tert*-butyl)-1-(3-chloropyridin-2-yl)-*N*′-(2-phenylacetyl)-1*H*-pyrazole-5-carbohydrazide **(10b)**

White solid, M.p: 211–213 °C; yield: 83%; ^1^H NMR (400 MHz, DMSO) δ 11.10 (s, 1H, N–H), 8.49 (dd, ^3^ *J* = 4.7 Hz, ^4^ *J* = 1.5 Hz, 1H, pyridine-H), 8.27 (dd, ^3^ *J* = 8.1 Hz, ^4^ *J* = 1.5 Hz, 1H, pyridine-H), 7.68 (dd, ^3^ *J* = 8.1 Hz, ^4^ *J* = 4.7 Hz, 1H, pyridine-H), 7.31 (s, 1H, benzene-H), 7.30–7.19 (m, 3H, benzene-H), 7.12–7.07 (m, 2H, benzene-H), 4.04 (s, 2H, –CH_2_–), 1.33 (s, 9H, 3CH_3_); ^13^C NMR (100 MHz, DMSO) δ 172.28, 157.85, 150.97, 147.72, 140.25, 137.77, 135.92, 129.97, 128.59, 127.96, 127.42, 126.82, 123.46, 111.46, 61.06, 40.94, 27.87. HR-MS (ESI^+^) *m/z* Calcd for C_21_H_21_BrClN_5_O_2_ [M + H]^+^ 490.06399; found 490.06392.

#### 3-Bromo-*N*′-(*tert*-butyl)-1-(3-chloropyridin-2-yl)-*N*′-(2,4,5-trifluorobenzoyl)-1*H*-pyrazole-5-carbohydrazide (**10c**)

White solid, M.p: 226–227 °C; yield: 85%; ^1^H NMR (400 MHz, DMSO) δ 11.18 (s, 1H, N–H), 8.45 (dd, ^3^ *J* = 4.7 Hz, ^4^ *J* = 1.5 Hz, 1H, pyridine-H), 8.19 (dd, ^3^ *J* = 8.1 Hz, ^4^ *J* = 1.5 Hz, 1H, pyridine-H), 7.67 (dd, ^3^ *J* = 8.1 Hz, ^4^ *J* = 4.7 Hz, 1H, pyridine-H), 7.65–7.59 (m, 1H, benzene-H), 7.20 (td, ^3^ *J* = 9.4 Hz, ^4^ *J* = 6.3 Hz, 1H, benzene-H), 7.03 (s, 1H, pyrazole-H), 1.42 (s, 9H, 3CH_3_); ^19^F NMR (471 MHz, DMSO-D6) δ −116.38, −132.12; ^13^C NMR (100 MHz, DMSO) δ 165.61, 163.14 (d, *J* = 229.6 Hz), 157.08, 153.64 (d, *J* = 243.2 Hz), 148.14, 147.62, 139.98, 136.94, 128.10, 127.49, 127.36, 122.50 (dd, *J* = 20.0, 4.3 Hz), 111.11, 116.74 (dd, *J* = 20.8, 5.8 Hz), 106.83 (dd, *J* = 28.6, 21.8 Hz) 61.97, 27.66; HR-MS (ESI^+^) *m/z* Calcd for C_20_H_16_BrClF_3_N_5_O_2_ [M + H]^+^ 530.02008; found ​530.02012.

#### *N*′-(3-Bromo-1-(3-chloropyridin-2-yl)-1*H*-pyrazole-5-carbonyl)-*N*-(*tert*-butyl)-2,6-dichloronicotinohydrazide (**10d**)

White solid. M.p: 223–224 °C; yield: 65%; ^1^H NMR (400 MHz, DMSO) δ 11.20 (s, 1H, N–H), 8.50 (d, ^3^ *J* = 3.5 Hz, 1H, pyridine-H), 8.21 (dd, ^3^ *J* = 8.1 Hz, ^4^ *J* = 1.4 Hz, 1H, pyridine-H), 7.68 (dd, ^3^ *J* = 8.1 Hz, ^4^ *J* = 4.7 Hz, 1H, pyridine-H), 7.56 (s, 1H, pyridine-H), 7.55 (s, 1H, pyridine-H), 6.99 (s, 1H, pyrazole-H), 1.44 (s, 9H, 3CH_3_). ^13^C NMR (100 MHz, DMSO) δ 166.76, 166.00, 165.37, 149.28, 148.40, 148.00, 147.98, 147.73, 140.17, 140.14, 139.45, 136.93, 136.91, 127.96, 127.53, 127.37, 123.72, 111.42, 62.07, 27.51; HR-MS (ESI^+^) *m/z* Calcd for C_19_H_16_BrCl_3_N_6_O_2,_ [M + H]^+^ 544.96565; found 544.96531; [M + Na]^+^ 566.94759; found 566.94752.

#### 3-Bromo-*N*′-(*tert*-butyl)-1-(3-chloropyridin-2-yl)-*N*′-(3,4,5-trifluorobenzoyl)-1H-pyrazole-5-carbohydrazide (**10e**)

White solid. M.p: 260–262; yield: 73%; ^1^H NMR (400 MHz, DMSO) δ 11.13 (s, 1H, N–H), 8.42 (dd, ^3^ *J* = 4.7 Hz, ^4^ *J* = 1.5 Hz, 1H, pyridine-H), 8.18 (dd, ^3^ *J* = 8.1 Hz, ^4^ *J* = 1.5 Hz, 1H, pyridine-H), 7.66 (dd, ^3^ *J* = 8.1 Hz, ^4^ *J* = 4.7 Hz, 1H, pyridine-H), 7.31–7.23 (m, 2H, benzene-H), 7.05 (s, 1H, pyrazole-H), 1.41 (s, 9H, 3CH_3_); ^19^F NMR (471 MHz, DMSO-D6) δ −116.37, −132.12, −142.79; ^13^C NMR (100 MHz, DMSO) δ 168.68, 156.82 (d, *J* = 245 Hz), 151.24 (d, *J* = 9.7 Hz) 148.08 (d, *J* = 245 Hz), 147.55, 139.95, 137.11, 128.11, 127.50, 127.46, 112.58, 112.36, 111.01, 100.00, 61.78, 27.61; HR-MS (ESI^+^) *m/z* Calcd for C_20_H_16_BrClF_3_N_5_O_2_, [M + H]^+^ 530.02008; found 530.02013; [M + Na]^+^ 552.00202, found 552.00243.

#### 3-Bromo-*N*′-(4-bromo-3-methylbenzoyl)-*N*′-(*tert*-butyl)-1-(3-chloropyridin-2-yl)-1*H*-pyrazole-5-carbohydrazide (**10f**)

White solid. M.p: 262–263 °C; yield: 72%; ^1^H NMR (400 MHz, DMSO) δ 10.88 (s, 1H, N–H), 8.53–8.44 (m, 1H, Ar–H), 8.27–8.15 (m, 1H, Ar–H), 7.67 (dd, ^3^ *J* = 12.2 Hz, ^4^ *J* = 7.3 Hz, 1H, pyridine-H), 7.52–7.41 (m, 1H, Ar–H), 7.33 (s, 1H, Ar–H), 6.98 (s, 1H, pyrazole-H), 6.70 (d, ^3^ *J* = 16.0 Hz, 1H, Ar–H), 2.17 (s, 3H, CH_3_), 1.44 (s, 9H, 3CH_3_). ^13^C NMR (100 MHz, DMSO) δ 171.28, 157.32, 147.98, 147.63, 140.04, 137.60, 133.14, 128.19, 127.96, 127.41, 127.20, 121.90, 110.79, 61.30, 27.76, 18.63; HR-MS (ESI^+^) *m/z* Calcd for C_21_H_20_Br_2_ClN_5_O_2_, [M + H]^+^ 567.97450; found 567.97471.

#### 3-Bromo-*N*′-(*tert*-butyl)-1-(3-chloropyridin-2-yl)-*N*′-(4-fluorobenzoyl)-1*H*-pyrazole-5-carbohydrazide (**10g**)

White solid, M.p: 256–257 °C; yield: 82%; ^1^H NMR (400 MHz, DMSO) δ 11.04 (s, 1H, N–H), 8.45 (dd, ^3^ *J* = 4.7 Hz, ^4^ *J* = 1.4 Hz, 1H, pyridine-H), 8.17 (dd, ^3^ *J* = 8.1 Hz, ^4^ *J* = 1.4 Hz, 1H, pyridine-H), 7.63 (dd, ^3^ *J* = 8.1 Hz,^4^ *J* = 4.7 Hz, 1H, pyridine-H), 7.46–7.37 (m, 2H, benzene-H), 7.19 (t, ^3^ *J* = 8.9 Hz, 2H, benzene-H), 6.90 (s, 1H, pyrazole-H), 1.41 (s, 9H, 3CH_3_); ^19^F NMR (471 MHz, DMSO-D6) δ −110.71; ^13^C NMR (100 MHz, DMSO) δ 170.98, 164.36, (d, ^1^ *J*_C-F_ = 246.7 Hz), 156.79, 148.08, 147.62, 139.95, 137.58, 133.68, 129.89, 129.81, 127.92, 127.33, 115.24 (d, ^2^ *J*_C-F_ = 21.7 Hz), 110.67, 61.31, 27.81; HR-MS (ESI^+^) *m/z* Calcd for C_20_H_18_BrClFN_5_O_2_, [M + H]^+^ 494.03892, found 494.03852.

#### 3-Bromo-*N*′-(*tert*-butyl)-1-(3-chloropyridin-2-yl)-*N*′-(2,3,4,5-tetrafluorobenzoyl)-1*H*-pyrazole-5-carbohydrazide (**10h**)

White solid, M.p: 185–187 °C; yield: 69%; ^1^H NMR (400 MHz, DMSO) δ 11.24 (s, 1H, N–H), 8.44 (dd, ^3^ *J* = 4.7 Hz, ^4^ *J* = 1.5 Hz, 1H, pyridine-H), 8.20 (dd, ^3^ *J* = 8.1, ^4^ *J* = 1.5 Hz, 1H, pyridine-H), 7.68 (dd, ^3^ *J* = 8.1, ^4^ *J* = 4.7 Hz, 1H, pyridine-H), 7.19–7.11 (m, 1H, benzene-H), 7.09 (s, 1H, pyrazole-H), 1.43 (s, 9H, 3CH_3_); ^19^F NMR (471 MHz, DMSO-D6) δ −138.96, −141.16, −154.38, −155.29; ^13^C NMR (126 MHz, DMSO-D6) δ 164.54, 157.29, 148.20, 147.65, 147.47–147.17, 145.68–144.33, 143.11–142.51, 141.91–140.72, 140.05, 139.83–139.15, 136.84, 128.23, 127.61, 127.50, 110.55 (d, *J* = 20.3 Hz), 62.35, 27.65; HR-MS (ESI^+^) *m/z* Calcd for C_20_H_15_BrClF_4_N_5_O_2_, [M + H]^+^ 548.01065, found 548.01032.

#### 3-Bromo-*N*′-(*tert*-butyl)-1-(3-chloropyridin-2-yl)-*N*′-(4-iodobenzoyl)-1*H*-pyrazole-5-carbohydrazide (**10i**)

White solid. M.p: 268–269 °C; yield: 76%; ^1^H NMR (400 MHz, DMSO) δ 11.05 (s, 1H, N–H), 8.44 (dd, ^3^ *J* = 4.7 Hz, ^4^ *J* = 1.5 Hz, 1H, pyridine-H), 8.16 (dd, ^3^ *J* = 8.1 Hz, ^4^ *J* = 1.5 Hz, 1H, pyridine-H), 7.73 (d, ^3^ *J* = 8.4 Hz, 2H, benzene-H), 7.63 (dd, ^3^ *J* = 8.1 Hz, ^4^ *J* = 4.7 Hz, 1H, pyridine-H), 7.15 (d, ^3^ *J* = 8.4 Hz, 2H, benzene-H), 6.90 (s, 1H, pyrazole-H), 1.41 (s, 9H, 3CH_3_); ^13^C NMR (100 MHz, DMSO) δ 171.22, 156.79, 148.06, 147.60, 139.96, 137.53, 136.98, 136.73, 129.23, 127.94, 127.34, 110.75, 97.17, 61.39, 27.77; HR-MS (ESI^+^) *m/z* Calcd for C_20_H_18_BrClIN_5_O_2_, [M + H]^+^ 601.94498, found 601.94452.

#### 3-Bromo-*N*′-(*tert*-butyl)-1-(3-chloropyridin-2-yl)-*N*′-(2-(thiophen-2-yl)acetyl)-1*H*-pyrazole-5-carbohydrazide (**10j**)

White solid, M.p: 219–220 °C; yield: 72%; ^1^H NMR (400 MHz, DMSO) δ 11.13 (s, 1H, N–H), 8.50 (dd, ^3^ *J* = 4.7 Hz, ^4^ *J* = 1.5 Hz, 1H, pyridine-H), 8.27 (dd, ^3^ *J* = 8.1 Hz, ^4^ *J* = 1.5 Hz, 1H, pyridine-H), 7.67 (dd, ^3^ *J* = 8.1 Hz, ^4^ *J* = 4.7 Hz, 1H, pyridine-H), 7.39 (dd, ^3^ *J* = 5.1 Hz, ^4^ *J* = 1.2 Hz, 1H), 7.35 (s, 1H, pyrazole-H), 6.95 (dd, ^3^ *J* = 5.1 Hz, ^4^ *J* = 3.4 Hz, 1H), 6.83 (dd, ^3^ *J* = 3.4 Hz, ^4^ *J* = 1.0 Hz, 1H), 3.95 (d, ^3^ *J* = 17.3 Hz, 1H), 3.54 (dd, ^3^ *J* = 17.0, ^4^ *J* = 0.7 Hz, 1H), 1.34 (s, 9H, 3CH_3_); ^13^C NMR (100 MHz, DMSO) δ 171.06, 157.86, 148.30, 147.73, 140.27, 137.69, 136.94, 127.92, 127.62, 127.43, 127.07, 126.88, 125.73, 111.55, 61.25, 35.27, 27.79; HR-MS (ESI^+^) *m/z* Calcd for C_19_H_19_BrClN_5_O_2_S, [M + H]^+^ 496.02041, found 496.02063.

#### 3-Bromo-*N*′-(4-bromo-5-fluoro-2-nitrobenzoyl)-*N*′-(*tert*-butyl)-1-(3-chloropyridin-2-yl)-1*H*-pyrazole-5-carbohydrazide (**10k**)

White solid. M.p: 126–127 °C yield: 68%; ^1^H NMR (400 MHz, DMSO) δ 11.05 (s, 1H, N–H), 8.62 (d, ^3^ *J* = 5.9 Hz, 1H, benzene-H), 8.47 (d, ^3^ *J* = 4.5 Hz, 1H, pyridine-H), 8.20 (d, ^3^ *J* = 8.0 Hz, 1H, pyridine-H), 7.70 (dd, ^3^ *J* = 8.1 Hz, ^4^ *J* = 4.7 Hz, 1H, pyridine-H), 7.13 (d, ^3^ *J* = 8.0 Hz, 1H, benzene-H), 7.07 (s, 1H, pyrazole-H), 1.45 (s, 9H, 3CH_3_); ^19^F NMR (471 MHz, DMSO-D6) δ −96.90; ^13^C NMR (100 MHz, DMSO) δ 166.32, 162.91, 160.36, 157.54, 148.23, 147.67, 140.43, 140.00, 136.55, 135.52, 135.43, 130.60, 128.27, 127.58, 127.31, 115.64, 115.38, 111.63, 109.91, 109.68, 100.00, 61.87, 27.25; HR-MS (ESI^+^) *m/z* Calcd for C_20_H_16_Br_2_ClFN_6_O_4_, [M + H]^+^ 616.93451, found 616.93433; [M + Na]^+^ 638.91464, found 638.91453.

#### *N*′-(4-(Benzyloxy)benzoyl)-3-bromo-*N*′-(*tert*-butyl)-1-(3-chloropyridin-2-yl)-1*H*-pyrazole-5-carbohydrazide (**10l**)

White solid. M.p: 236–238 °C yield: 68%; ^1^H NMR (400 MHz, DMSO) δ 10.99 (s, 1H, N–H), 8.43 (dd, ^3^ *J* = 4.7 Hz, ^4^ *J* = 1.5 Hz, 1H, pyridine-H), 8.15 (dd, ^3^ *J* = 8.1 Hz, ^4^ *J* = 1.5 Hz, 1H, pyridine-H), 7.62 (dd, ^3^ *J* = 8.1 Hz, ^4^ *J* = 4.7 Hz, 1H, pyridine-H), 7.46–7.31 (m, 7H, benzene-H), 7.00–6.93 (m, 2H, benzene-H), 6.91 (s, 1H, pyrazole-H), 5.12 (s, 2H, –CH_2_–), 1.41 (s, 9H, 3CH_3_); ^13^C NMR (100 MHz, DMSO) δ 171.50, 159.95, 156.79, 148.13, 147.60, 139.93, 137.84, 137.21, 129.49, 129.44, 128.90, 128.38, 128.23, 127.89, 127.30, 127.27, 114.21, 110.61, 69.72, 61.11, 27.91; HR-MS (ESI^+^) *m/z* Calcd for C_27_H_25_BrClN_5_O_3_, [M + H]^+^ 582.09021, found 582.09052.

#### 3-Bromo-*N*′-(*tert*-butyl)-*N*′-(4-chloro-3-fluorobenzoyl)-1-(3-chloropyridin-2-yl)-1*H*-pyrazole-5-carbohydrazide (**10m**)

White solid. M.p: 269–270 °C; yield: 72%; ^1^H NMR (400 MHz, DMSO) δ 11.12 (s, 1H, N–H), 8.44 (dd, ^3^ *J* = 4.7 Hz, ^4^ *J* = 1.5 Hz, 1H, pyridine-H), 8.16 (dd, ^3^ *J* = 8.1 Hz, ^4^ *J* = 1.5 Hz, 1H, pyridine-H), 7.64 (dd, ^3^ *J* = 8.1 Hz, ^4^ *J* = 4.7 Hz, 1H, pyridine-H), 7.57 (dd, ^3^ *J* = 7.2 Hz, ^4^ *J* = 1.9 Hz, 1H, benzene-H), 7.49–7.33 (m, 2H, benzene-H), 6.98 (s, 1H, pyrazole-H), 1.42 (s, 9H, 3CH_3_); ^19^F NMR (471 MHz, DMSO-D6) δ −113.90;^13^C NMR (100 MHz, DMSO) δ 169.60, 158.27 (d, *J*_*C*-*F*_ = 249.5 Hz), 157.03, 156.69, 148.04, 147.62, 139.92, 137.36, 134.80, 134.76, 129.79, 128.49, 128.41, 127.99, 127.39, 119.40 (d, *J*_C-F_ = 18.1 Hz), 119.31, 116.94 (d, *J*_C-F_ = 21.4 Hz), 116.83, 110.81, 61.55, 40.60, 40.39, 40.19, 39.98, 39.77, 39.56, 39.35, 27.72; HR-MS (ESI^+^) *m/z* Calcd for C_20_H_17_BrCl_2_FN_5_O_2_, [M + H]^+^ 527.9995, found 528.0013; [M + H]^+^ 549.98189, found 549.98161.

#### *N*′-(3-Bromo-1-(3-chloropyridin-2-yl)-1*H*-pyrazole-5-carbonyl)-*N*-(*tert*-butyl)-1-methyl-1*H*-pyrazole-3-carbohydrazide (**10n**)

White solid. M.p: 234–235 °C yield: 74%; ^1^H NMR (400 MHz, DMSO) δ 11.17 (s, 1H, N–H), 8.46 (dd, ^3^ *J* = 4.7 Hz, ^4^ *J* = 1.5 Hz, 1H, pyridine-H), 8.19 (dd, ^3^ *J* = 8.1 Hz, ^4^ *J* = 1.5 Hz, 1H, pyridine-H), 7.64 (dd, ^3^ *J* = 8.1 Hz, ^4^ *J* = 4.7 Hz, 1H, pyridine-H), 7.37 (d, ^3^ *J* = 2.0 Hz, 1H, pyrazole-H), 7.07 (s, 1H, pyrazole-H), 6.44 (d, ^3^ *J* = 2.0 Hz, 1H, pyrazole-H), 3.69 (s, 3H), 1.42 (s, 9H, 3CH_3_); ^13^C NMR (100 MHz, DMSO) δ 164.05, 157.45, 148.12, 147.63, 139.98, 137.51, 137.27, 136.68, 127.92, 127.43, 127.29, 110.96, 106.38, 61.66, 38.07, 27.74; HR-MS (ESI^+^) *m/z* Calcd for C_18_H_19_BrClN_7_O_2_, [M + H]^+^ 480.05449, found 480.05432.

#### *N*′-(3-Bromo-1-(3-chloropyridin-2-yl)-1*H*-pyrazole-5-carbonyl)-*N*-(*tert*-butyl) nicotinohydrazide (**10o**)

White solid. M.p: 203–205 °C; yield: 81%; ^1^H NMR (400 MHz, DMSO) δ 11.19 (s, 1H, N–H), 8.63–8.50 (m, 2H, pyridine-H), 8.47–8.39 (m, 1H, pyridine-H), 8.21–8.11 (m, 1H, pyridine-H), 7.74 (d, ^3^ *J* = 7.9 Hz, 1H, pyridine-H), 7.63 (dd, ^3^ *J* = 8.1 Hz, ^4^ *J* = 4.7 Hz, 1H, pyridine-H), 7.40 (dd, ^3^ *J* = 7.5 Hz, ^4^ *J* = 5.1 Hz, 1H, pyridine-H), 6.92 (s, 1H, pyrazole-H), 1.44 (s, 9H, 3CH_3_); ^13^C NMR (100 MHz, DMSO) δ 170.02, 156.86, 150.96, 147.99, 147.82, 147.65, 139.98, 137.33, 134.79, 133.04, 127.85, 127.34, 127.30, 123.45, 110.81, 61.56, 27.76; HR-MS (ESI^+^) *m/z* Calcd for C_19_H_18_BrClN_6_O_2_, [M + H]^+^ 477.04359, found 477.04385; [M + Na]^+^ 499.02554, found 499.02576.

#### 3-Bromo-*N*′-(*tert*-butyl)-1-(3-chloropyridin-2-yl)-*N*′-(3-(trifluoromethyl)benzoyl)-1*H*-pyrazole-5-carbohydrazide (**10p**)

White solid. M.p: 274–276 °C; yield: 67%; ^1^H NMR (400 MHz, DMSO) δ 11.15 (s, 1H, N–H), 8.43 (dd, ^3^ *J* = 4.7 Hz, ^4^ *J* = 1.4 Hz, 1H, pyridine-H), 8.13 (dd, ^3^ *J* = 8.1 Hz, ^4^ *J* = 1.4 Hz, 1H, pyridine-H), 7.81–7.72 (m, 2H, benzene-H), 7.68–7.56 (m, 3H, benzene-H), 6.87 (s, 1H, pyrazole-H), 1.44 (s, 9H, 3CH_3_); ^19^F NMR (471 MHz, DMSO-D6) δ −61.02; ^13^C NMR (100 MHz, DMSO) δ 170.37, 156.69, 148.03, 147.62, 139.88, 138.10, 137.31, 131.42, 129.57, δ 128.88 (q, *J*_*C*-*F*_ = 32.0 Hz), 128.40, 127.94, 127.34, 127.02 (q, *J*_*C*-*F*_ = 7.6 Hz), 125.75, 124.40 (q, *J*_*C*-*F*_ = 272.5 Hz),123.90 (q, *J*_*C*-*F*_ = 7.6 Hz), 123.04, 110.68, 61.53, 27.73; HR-MS (ESI^+^) *m/z* Calcd for C_21_H_18_BrClF_3_N_5_O_2_, [M + H]^+^ 544.03573, found 544.03551.

#### *N*′-(3-Bromo-1-(3-chloropyridin-2-yl)-1*H*-pyrazole-5-carbonyl)-*N*-(*tert*-butyl)-2,6-dichloroisonicotinohydrazide (**10q**)

White solid. M.p: 235–236 °C; yield: 65%; ^1^H NMR (400 MHz, DMSO) δ 11.15 (s, 1H, N–H), 8.46 (dd, ^3^ *J* = 4.7 Hz, ^4^ *J* = 1.5 Hz, 1H, pyridine-H), 8.18 (dd, ^3^ *J* = 8.1 Hz, ^4^ *J* = 1.5 Hz, 1H, pyridine-H), 7.67 (dd, ^3^ *J* = 8.1 Hz, ^4^ *J* = 4.7 Hz, 1H, pyridine-H), 7.42 (s, 2H, pyridine-H), 7.07 (s, 1H, pyrazole-H), 1.42 (s, 9H, 3CH_3_). ^13^C NMR (100 MHz, DMSO) δ 167.14, 156.91, 150.64, 149.55, 148.02, 147.74, 139.95, 136.82, 128.10, 127.50, 121.20, 111.26, 62.20, 27.50; HR-MS (ESI^+^) *m/z* Calcd for C_19_H_16_BrCl_3_N_6_O_2_, [M + H]^+^ 544.96565, found 544.96541.

#### 3-Bromo-*N*′-(*tert*-butyl)-1-(3-chloropyridin-2-yl)-*N*′-(2-(trifluoromethyl)benzoyl)-1*H*-pyrazole-5-carbohydrazide (**10r**)

White solid. M.p: 260–262 °C; yield: 74%; ^1^H NMR (400 MHz, DMSO) δ 10.87 (s, 1H, N–H), 8.52 (s, 1H, pyridine-H), 8.23 (s, 1H, pyridine-H), 7.80–7.65 (m, 2H, benzene-H + pyridine-H), 7.57 (d, ^3^ *J* = 6.6 Hz, 2H, benzene-H), 7.13 (s, 1H, pyrazole-H), 6.66 (s, 1H, benzene-H), 1.44 (s, 9H, 3CH_3_); ^13^C NMR (100 MHz, DMSO) δ 170.37, 156.69, 148.03, 147.62, 139.88, 138.10, 137.31, 131.42, 129.57, δ 128.88 (q, *J*_*C*-*F*_ = 32.0 Hz), 128.40, 127.94, 127.34, 127.02 (q, *J*_*C*-*F*_ = 7.6 Hz), 125.75, 124.40 (q, *J*_*C*-*F*_ = 272.5 Hz),123.90 (q, *J*_*C*-*F*_ = 7.6 Hz), 123.04, 110.68, 61.53, 27.73; HR-MS (ESI^+^) *m/z* Calcd for C_21_H_18_BrClF_3_N_5_O_2_, [M + H]^+^ 544.03573, found 544.03557.

#### 3-Bromo-*N*′-(5-bromo-2-fluorobenzoyl)-*N*′-(*tert*-butyl)-1-(3-chloropyridin-2-yl)-1*H*-pyrazole-5-carbohydrazide (**10s**)

White solid. M.p: 223–224 °C yield: 72%; ^1^H NMR (400 MHz, DMSO) δ 11.14 (s, 1H, N–H), 8.47 (dd, ^3^ *J* = 4.7 Hz, ^4^ *J* = 1.5 Hz, 1H, pyridine-H), 8.19 (dd, ^3^ *J* = 8.1 Hz, ^4^ *J* = 1.5 Hz, 1H, pyridine-H), 7.65 (dd, ^3^ *J* = 8.1 Hz, ^4^ *J* = 4.7 Hz, 1H, pyridine-H), 7.62 (dd, ^3^ *J* = 9.4 Hz, ^4^ *J* = 1.8 Hz, 1H, Ar–H), 7.38 (dd, ^3^ *J* = 8.2 Hz, ^4^ *J* = 1.8 Hz, 1H, Ar–H), 7.11 (t, ^3^ *J* = 7.8 Hz, 1H, Ar–H), 6.92 (s, 1H, pyrazole-H), 1.42 (s, 9H, 3CH_3_); ^13^C NMR (100 MHz, DMSO) δ 166.85, 157.95 (d, *J*_*C*-*F*_ = 251.7 Hz) 157.14, 148.06, 147.64, 140.01, 137.21, 130.03 127.97, 127.78, 127.42, 127.31, 125.14 (d, *J*_*C*-*F*_ = 17.4 Hz), 123.17 (d, *J*_*C*-*F*_ = 9.4 Hz), 119.41 (d, *J*_*C*-*F*_ = 25.0 Hz) 111.01, 61.80, 27.69; HR-MS (ESI^+^) *m/z* Calcd for C_20_H_17_Br_2_ClFN_5_O_2_, [M + H]^+^ 571.94943, found 571.94928, [M + Na]^+^ 593.93138, found 593.93181.

#### 3-Bromo-*N*′-(*tert*-butyl)-1-(3-chloropyridin-2-yl)-*N*′-(furan-3-carbonyl)-1*H*-pyrazole-5-carbohydrazide (**10t**)

White solid. M.p: 221–223 °C yield: 73%; ^1^H NMR (400 MHz, DMSO) δ 11.21 (s, 1H, N–H), 8.45 (dd, ^3^ *J* = 4.7 Hz, ^4^ *J* = 1.5 Hz, 1H, pyridine-H), 8.19 (dd, ^3^ *J* = 8.1 Hz, ^4^ *J* = 1.5 Hz, 1H, pyridine-H), 7.96 (dd, ^3^ *J* = 1.5 Hz, ^4^ *J* = 0.8 Hz, 1H, furan-H), 7.67–7.65 (m, 1H, Furan-H), 7.63 (dd, ^3^ *J* = 8.1 Hz, ^4^ *J* = 4.7 Hz, 1H, pyridine-H), 7.31 (s, 1H, pyrazole-H), 6.65 (dd, ^3^ *J* = 1.9 Hz, ^4^ *J* = 0.8 Hz, 1H, furan-H), 1.39 (s, 9H, 3CH_3_). ^13^C NMR (100 MHz, DMSO) δ 164.93, 157.48, 148.39, 147.62, 145.52, 143.52, 139.97, 137.53, 128.06, 127.61, 127.36, 122.44, 110.99, 61.47, 27.92; HR-MS (ESI^+^) *m/z* Calcd for C_18_H_17_BrClN_5_O_3_, [M + H]^+^ 466.02761, found 466.02732, [M + Na]^+^ 488.00955, found 488.00913.

#### *N*′-(3-bromo-1-(3-chloropyridin-2-yl)-1*H*-pyrazole-5-carbonyl)-*N*-(*tert*-butyl)-4-(trifluoromethyl)nicotinohydrazide (**10u**)

White solid. M.p: 187–189 °C; yield: 70%; ^1^H NMR (400 MHz, DMSO) δ 11.07 (s, 1H, N–H), 8.84 (d, ^3^ *J* = 5.1 Hz, 1H, pyridine-H), 8.50 (s, 1H, pyridine-H), 8.21 (d, ^3^ *J* = 7.7 Hz, 1H, pyridine-H), 7.80 (d, ^3^ *J* = 5.1 Hz, 1H, pyridine-H), 7.67 (dd, ^3^ *J* = 7.9 Hz, ^3^ *J* = 4.7 Hz, 1H, pyridine-H), 6.84 (s, 1H, pyrazole-H), 1.45 (s, 9H, 3CH_3_); ^19^F NMR (471 MHz, DMSO-D6) δ −60.17; ^13^C NMR (100 MHz, DMSO) δ 170.83, 167.31, 151.50, 147.93, 147.76, 140.13, 137.06, 129.88, 127.87, 127.38, 127.28, 120.75, 111.24, 62.12, 27.34; HR-MS (ESI^+^) *m/z* Calcd for C_20_H_17_BrClF_3_N_6_O_2_, [M + H]^+^ 545.03098, found 545.03062.

#### 3-Bromo-*N*′-(*tert*-butyl)-1-(3-chloropyridin-2-yl)-*N*′-(3,4-dichlorobenzoyl)-1*H*-pyrazole-5-carbohydrazide (**10v**)

White solid. M.p: 228–225 °C; yield: 71%; ^1^H NMR (400 MHz, DMSO) δ 11.08 (s, 1H, N–H), 8.36 (dd, *J* = 4.7, 1.5 Hz, 1H, pyridine-H), 8.08 (dd, ^3^ *J* = 8.1 Hz, ^4^ *J* = 1.5 Hz, 1H, pyridine-H), 7.58 (dd, ^3^ *J* = 3.4 Hz, ^4^ *J* = 1.3 Hz, 1H, Ar–H), 7.56 (dd, ^3^ *J* = 3.2 Hz, ^4^ *J* = 1.4 Hz, 1H, Ar–H), 7.51 (d, ^4^ *J* = 2.0 Hz, 1H, Ar–H), 7.29 (d, ^4^ *J* = 1.1 Hz, 1H, Ar–H), 7.26 (dd, ^3^ *J* = 8.3, ^4^ *J* = 2.0 Hz, 1H, Ar–H), 6.91 (s, 1H, pyrazole-H), 1.34 (s, 9H, 3CH_3_). ^13^C NMR (100 MHz, DMSO) δ 169.54, 156.69, 148.02, 147.61, 139.92, 137.56, 137.30, 132.93, 131.05, 130.64, 129.32, 128.13, 128.00, 127.55, 127.40, 127.12, 110.86, 61.63, 27.69; HR-MS (ESI^+^) *m/z* Calcd for C_20_H_17_BrCl_3_N_5_O_2_, [M + H]^+^ 543.97040, found 543.97081, [M + Na]^+^ 565.95234, found 565.95271.

#### *N*′-Benzoyl-3-bromo-*N*′-(*tert*-butyl)-1-(3-chloropyridin-2-yl)-1*H*-pyrazole-5-carbohydrazide (**10w**)

White solid. M.p: 269–270 °C; yield: 78%; ^1^H NMR (400 MHz, DMSO) δ 11.00 (s, 1H, N–H), 8.45 (dd, ^3^ *J* = 4.7 Hz, ^4^ *J* = 1.5 Hz, 1H, pyridine-H), 8.17 (dd, ^3^ *J* = 8.1 Hz, ^4^ *J* = 1.5 Hz, 1H, pyridine-H), 7.63 (dd, ^3^ *J* = 8.1, ^4^ *J* = 4.7 Hz, 1H, pyridine-H), 7.42–7.34 (m, 5H, benzene-H), 6.79 (s, 1H, pyrazole-H), 1.43 (s, 9H, 3CH_3_); ^13^C NMR (100 MHz, DMSO) δ 181.36, 172.00, 156.91, 148.08, 147.62, 139.98, 137.72, 137.38, 130.11, 128.13, 127.90, 127.29, 127.21, 127.12, 110.58, 61.17, 27.83; HR-MS (ESI^+^) *m/z* Calcd for C_20_H_19_BrClN_5_O_2_, [M + H]^+^ 476.04834, found 476.04871, [M + Na]^+^ 498.03029, found 498.03072.

#### 3-Bromo-*N*′-(2-bromo-5-chlorobenzoyl)-*N*′-(*tert*-butyl)-1-(3-chloropyridin-2-yl)-1*H*-pyrazole-5-carbohydrazide (**10x**)

White solid. M.p: 208–210 °C; yield: 72%; ^1^H NMR (400 MHz, DMSO) δ 11.03 (s, 1H, N–H), 8.52 (d, ^3^ *J* = 3.9 Hz, 1H, benzene-H), 8.21 (dd, ^3^ *J* = 8.1 Hz, ^4^ *J* = 1.4 Hz, 1H, pyridine-H), 7.67 (dd, ^3^ *J* = 8.1 Hz, ^4^ *J* = 4.7 Hz, 1H, pyridine-H), 7.56 (dd, ^3^ *J* = 8.6 Hz, ^4^ *J* = 2.4 Hz, 1H, pyridine-H), 7.42 (d, ^3^ *J* = 8.5 Hz, 1H, benzene-H), 6.90 (s, 1H, pyrazole-H), 1.45 (s, 9H, 3CH_3_). ^13^C NMR (100 MHz, DMSO) δ 167.44, 157.30, 148.15, 147.75, 140.01, 137.04, 133.41, 131.50, 129.59, 128.21, 127.40, 127.22, 119.95, 111.11, 56.51, 27.56; HR-MS (ESI^+^) *m/z* Calcd for C_20_H_17_Br_2_Cl_2_N_5_O_2_, [M + H]^+^ 587.91988, found 587.91951.

### Biological assay

All bioassays were conducted on test organisms reared in the lab and repeated at 25 ± 1 °C according to statistical requirements. Mortalities were corrected using Abbott’s formula [37]. Evaluations were based on a percentage scale (0 = no activity and 100 = complete eradication), at intervals of 5%.

#### Insecticidal activity against *H. armigera*

The insecticidal activities of some of the synthesised compounds and avermectins against *Helicoverpa armigera* were evaluated by the diet-incorporated method [33]. A quantity of 3 mL of prepared solutions containing the compounds was added to the forage (27 g), subsequently diluted to different concentrations and then placed in a 24-pore plate. One larva was placed in each of the wells on the plate. Mortalities were determined after 72–96 h.

#### Insecticidal activity against *P. xylostella*

The insecticidal activities of compounds **10a**–**10y** against third instar larvae of *P. xylostella* were evaluated according to a previously reported procedure [33–35]. Fresh cabbage discs (diameter: 2 cm) were dipped into the prepared solutions containing compounds **10a**–**10y** for 10 s, air-dried, and then placed in a Petri dish (diameter: 9 cm) lined with filter paper. Then, ten third instar larvae of *P. xylostella* were carefully transferred to the Petri dish. Each assay was conducted in triplicate. Mortality was calculated 72 h after treatment. The control groups were treated with distilled water containing TW-80 (0.1 mL/L). Commercial insecticides (i.e., chlorantraniliprole, chlorpyrifos, and avermectins) were tested and compared under the same conditions.

## Conclusions

Twenty-four novel 3-bromo-1-(3-chloropyridin-2-yl)-1*H*-pyrazole-5-carbohydrazide derivatives (**10a**–**10x**) were designed and synthesized based on combinating the sub-structures of chlorantraniliprole and diacylhydrazines. These compounds were characterized and confirmed by ^1^H NMR, ^13^C NMR, HR-MS. A preliminary evaluation of the insecticidal activities of the synthesized compounds was conducted. Most compounds exhibited good insecticidal activity against *Helicoverpa armigera* and *P. xylostella*. In particular, the LC_50_ values of compounds **10e**, **10g**, **10h**, **10j** and **10x** were 86.98, 27.49, 23.67, 69.07, and 28.90 mg L^−1^, respectively. Notably, compounds **10g**, **10h**, and **10x** showed much higher insecticidal activity than that of tebufenozide (LC_50_ = 37.77 mg L^−1^). Preliminary SAR analysis indicated that phenyl, 4-fluoro phenyl and four fluorophenyl had positive influence on the insecticidal activity of synthesized compounds, and introduction of a heterocyclic ring (pyridine and pyrazole) could decrease their insecticidal effects. Further structural modification and biological evaluation to explore the full potential of this kind of 3-bromo-1-(3-chloropyridin-2-yl)-1*H*-pyrazole-5-carbohydrazide derivatives are currently underway.

## Additional file

**Additional file 1.** All the copies of ^1^H NMR, ^19^F NMR and ^13^C NMR for the title compounds were presented in Additional information.
