# Supplementary material for: Synthesis and insecticidal activity of diacylhydrazine derivatives containing a 3-bromo-1-(3-chloropyridin-2-yl)-1H-pyrazole scaffold
Source: Chem Cent J. 2017 Jun 5;11:50. doi: 10.1186/s13065-017-0279-z (PMC5459783; doi:10.1186/s13065-017-0279-z)
Supplement: Supplementary file 1 — Additional file 1. All the copies of 1H NMR, 19F NMR and 13C NMR for the title compounds were presented in Additional information. [file 13065_2017_279_MOESM1_ESM.doc]

**Additional file**

**Synthesis and insecticidal activity of diacylhydrazine derivatives containing a 3-bromo-1-(3-chloropyridin-2-yl)-1*H*-pyrazole scaffold**

Yanyan Wang ^1#^, Fanzhou Xu^1#^, Gang Yu^1^, Jun Shi^1^, Chuanhui Li^1^, A’li Dai^1^, Zhiqian Liu, Jiahong Xu^1^, Fenghua Wang^1^, and Jian Wu^1^*

*^1^Key Laboratory of Green Pesticide and Agricultural Bioengineering, Ministry of Education, Research and Development Center for Fine Chemicals, Guizhou University, Guiyang 550025, China.*

*^#^* Co-first author for this manuscript.

*To whom correspondence should be addressed. Tel.: +86(851)88292090; fax: +86(851) 88292090. E-mails: [wujian2691@126.com](mailto:wujian2691@126.com), [jwu6@gzu.edu.cn](mailto:jwu6@gzu.edu.cn)

**1. Preparation of the intermediates**

*1.1 Preparation of 3-chloro-2-hydrazinylpyridine*

To a suspension of 2,3-dichloropyridine (7.4 g, 0.05 mol) in anhydrous ethanol (50 mL) was added 50% hydrazine hydrate (20 mL). The resulting mixture was refluxed for 10 h and then cooled to room temperature. The white crystal product precipitated out of solution and was collected by filtration, washed thoroughly with cold ethanol and dried to give 3-chloro-2-hydrazinylpyridine in 85% yield, m.p. 160–1162 °C.

*1.2 Preparation of ethyl 2-(3-chloropyridin-2-yl)-5-oxopyrazolidine-3-carboxylate*

To 60 mL of absolute ethanol was added 1.7 g (0.3 mol) of sodium. The mixture was heated to reflux for 20 min, and 3-chloro-2-hydrazinylpyridine (9 g) was added. Then the mixture was refluxed for 10 min, and subsequently diethyl maleate (11.7 g, 0.3 mol) was added slowly. The resulting solution was refluxed for another 20 min and treated with glacial acetic acid (6.6 g) after cooling to 65 °C. The mixture was then diluted with water (100 mL) and frozen in a refrigerator for 24 h. The solid was collected by filtration and washed with aqueous ethanol (50%, 50 mL × 3) to give ethyl 2-(3-chloro-2- pyridinyl)-5-oxopyrzolidine-3-carboxylate (4) in 50% yield.

*1.3 Preparation of ethyl 3-bromo-1-(3-chloropyridin-2-yl)-4,5-dihydro-1H-pyrazole- 5-carboxylate*

To a solution of 4 (0.05 mol) in acetonitrile (150 mL) was added phosphorus oxyhalide (0.06 mmol). The resulting mixture was refluxed for 5 h, and then about 120 mL of solvent was removed by distillation. The residue was slowly poured into saturated aqueous Na_2_CO_3_ (120 mL), stirred vigorously for 30 min and extracted with CH_2_Cl_2_ (50 mL × 3), and the organic extract was separated, dried, filtered and concentrated to afford intermediates 5.

*1.4 Preparation of ethyl 3-bromo-1-(3-chloropyridin-2-yl)-1H-pyrazole-5-carboxylate*

To a mixture of 5 (4 g, 1 mmol) in acetonitrile (30 mL) was slowly added concentrated H_2_SO_4_ (2 g) and potassium persulfate (2.5 mmol), and the resulting mixture was refluxed for 4 h. The potassium salt formed was separated by filtration after cooling to 60 °C and then dissolved in water (60 mL), and the precipitated solid was easily separated from the mixture to afford 6 in 80% yield, m.p. 117.5–118.6 °C.

*1.5 Preparation of 3-bromo-1-(3-chloropyridin-2-yl)-1H-pyrazole-5-carboxylic acid*

To a well-stirred suspension of 6 (3.6 g) in water (10 mL) were added sodium hydroxide (0.7 g) and methanol (20 mL). The resulting mixture was stirred for 4–6 h at room temperature and then concentrated in vacuo to about 10 mL, and the resulting mixture was diluted with H_2_O (20 mL) and subsequently acidified using concentrated hydrochloric acid to pH 1.5. The precipitated solid was collected by filtration, washed with water (15 mL) and then dried to give pyrazolecarboxylic acids 7 in 70% yield, m.p. 200–201 °C.

**2. The copies of ^1^H NMR, ^19^F NMR and ^13^C NMR for the title compounds.**

**
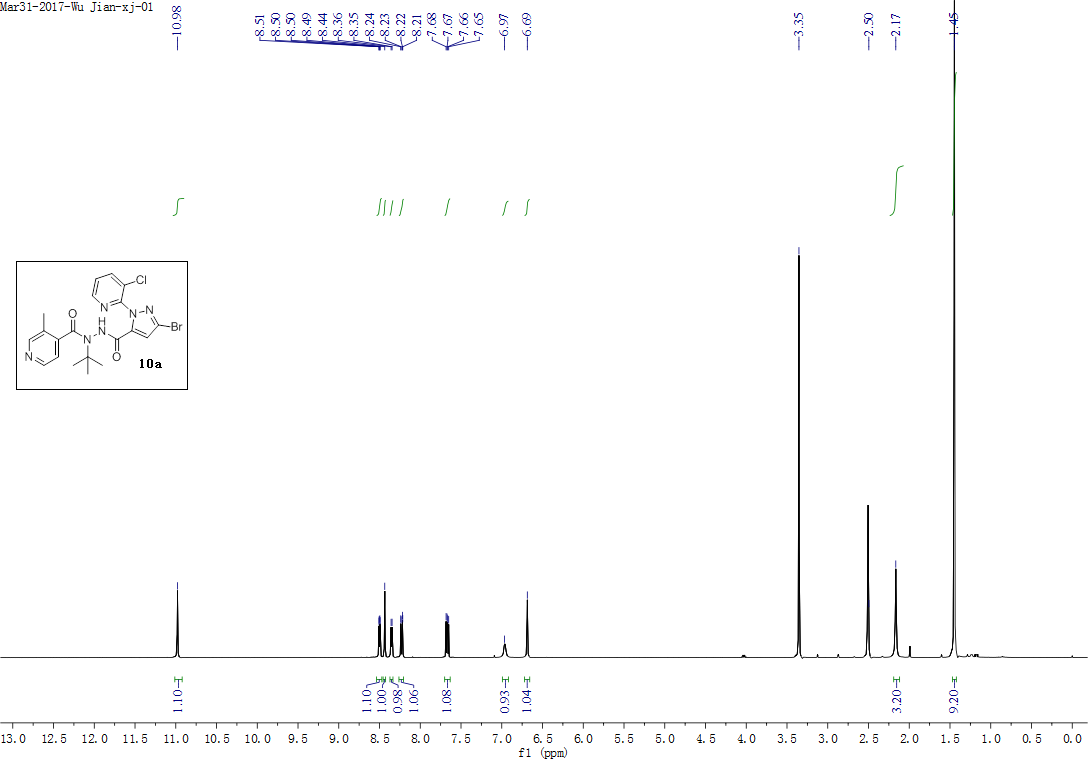
**

**Fig. S1** The copy of ^1^H NMR for compound 10a


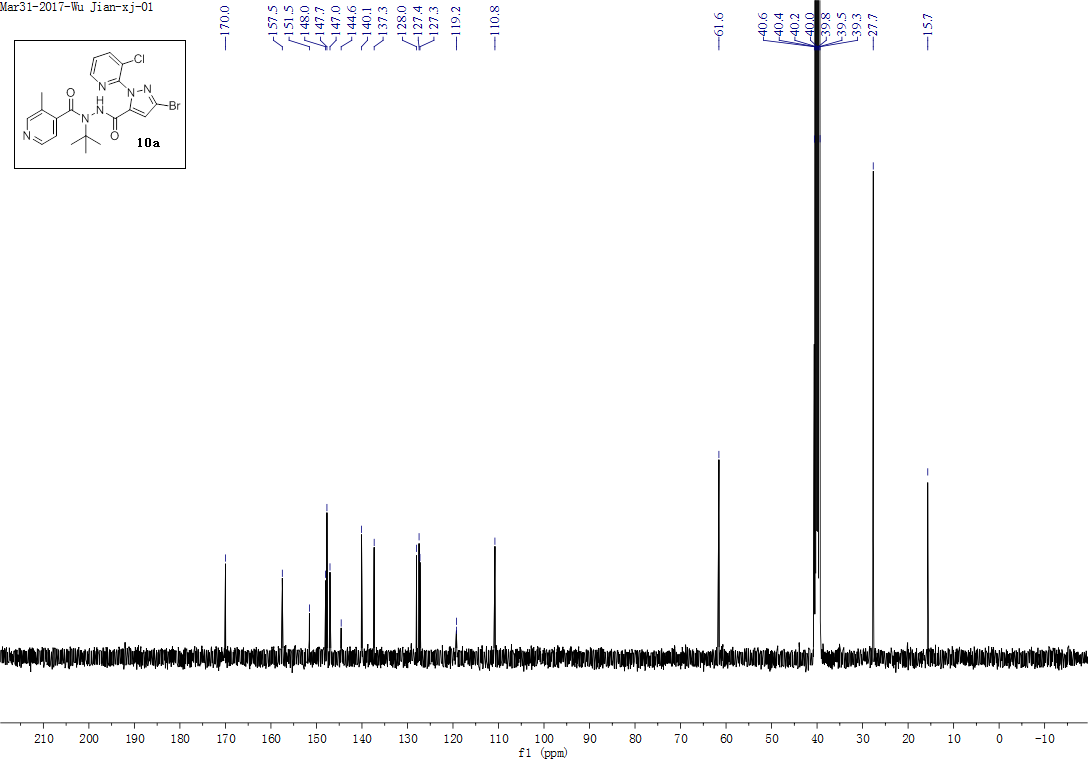


**Fig. S2** The copy of ^13^C NMR for compound 10a


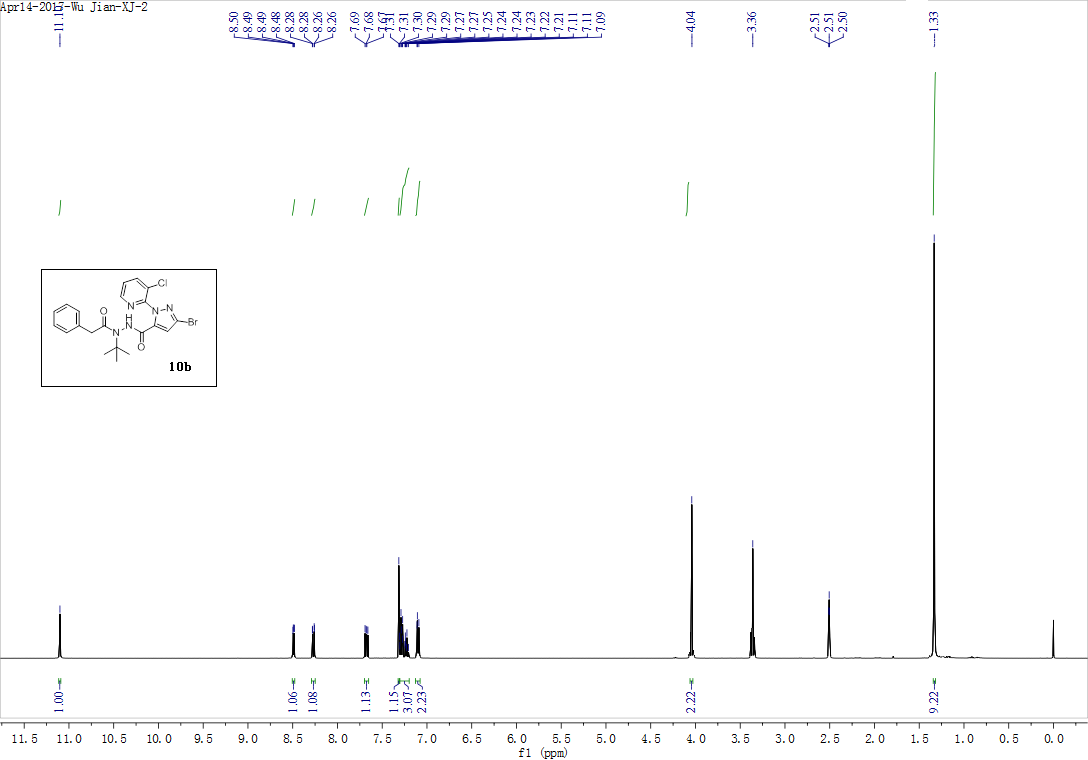


**Fig. S3** The copy of ^1^H NMR for compound **10b**


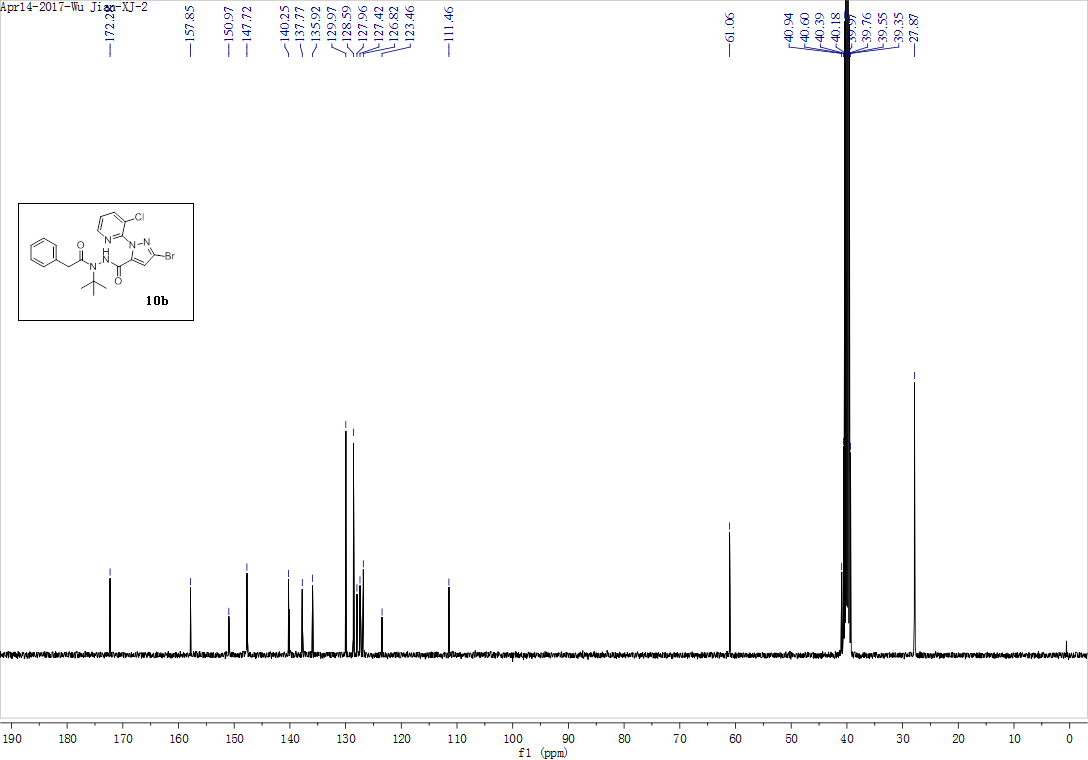


**Fig. S4** The copy of ^13^C NMR for compound **10b**


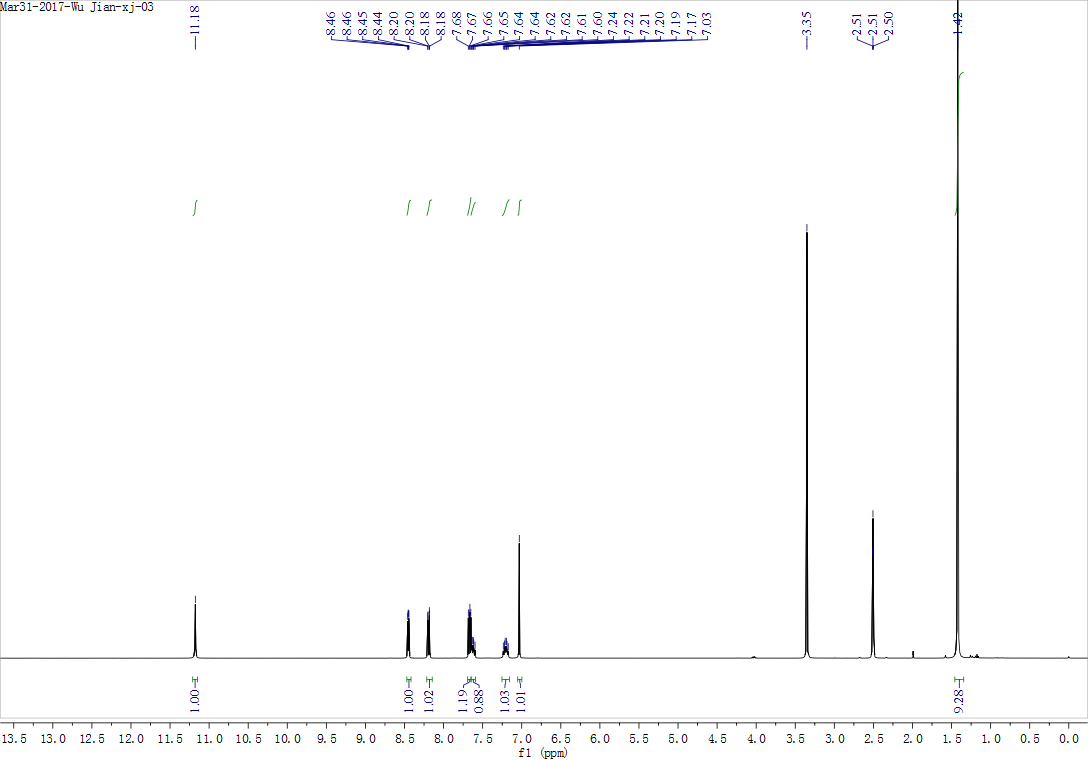


**Fig. S5** The copy of ^1^H NMR for compound **10c**


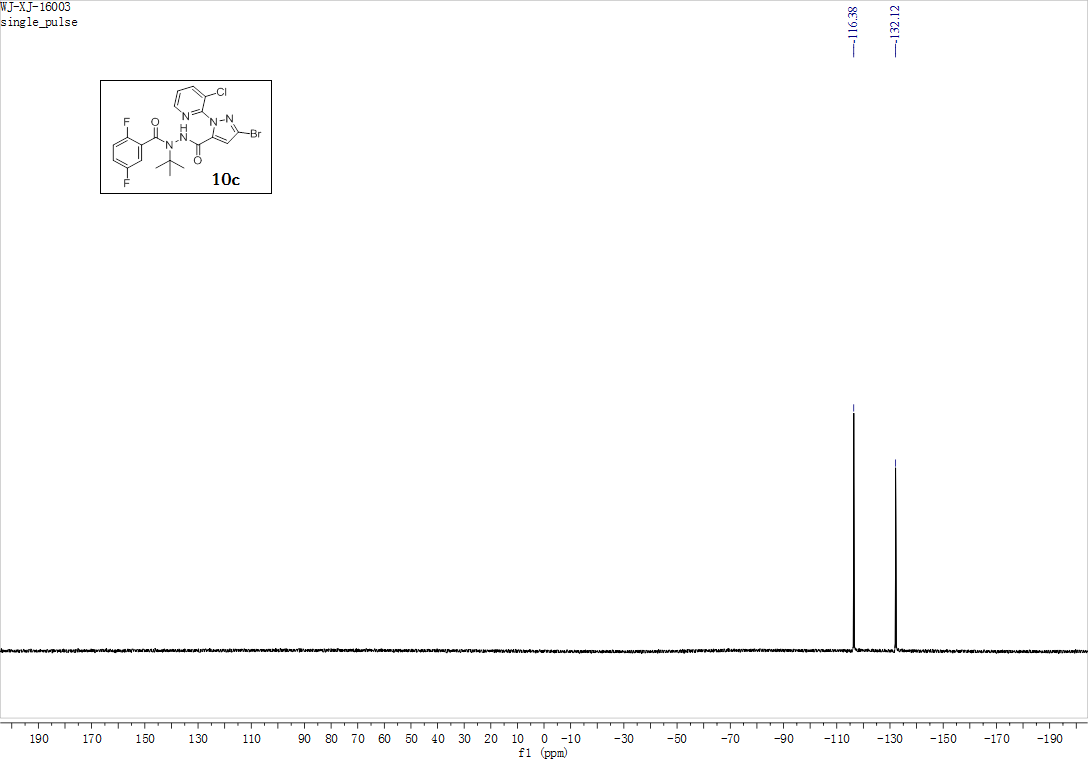


**Fig. S6** The copy of ^19^F NMR for compound **10c**


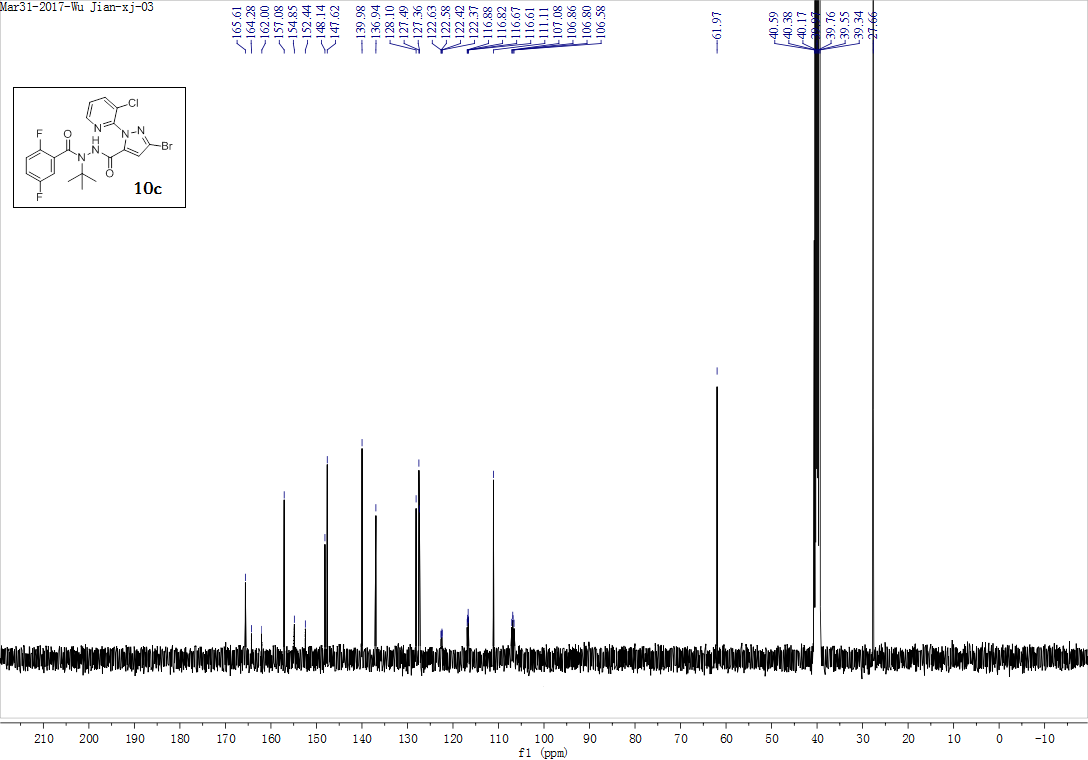


**Fig. S7** The copy of ^13^C NMR for compound **10c**


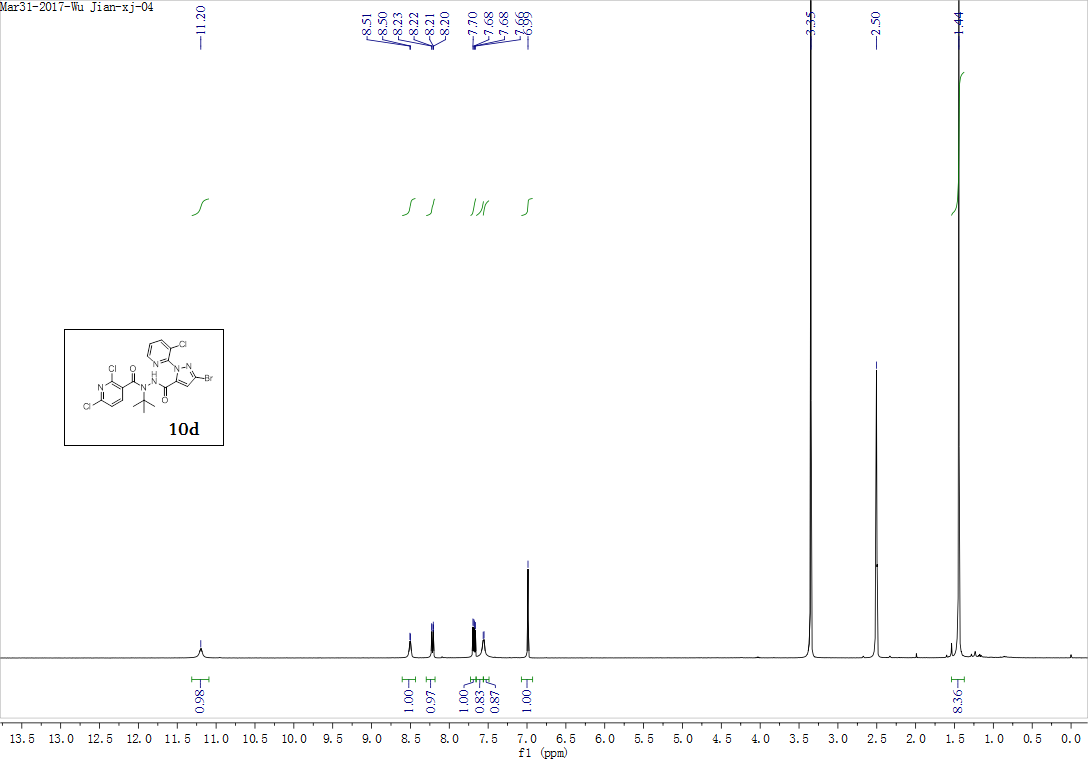


**Fig. S8** The copy of ^1^H NMR for compound **10d**


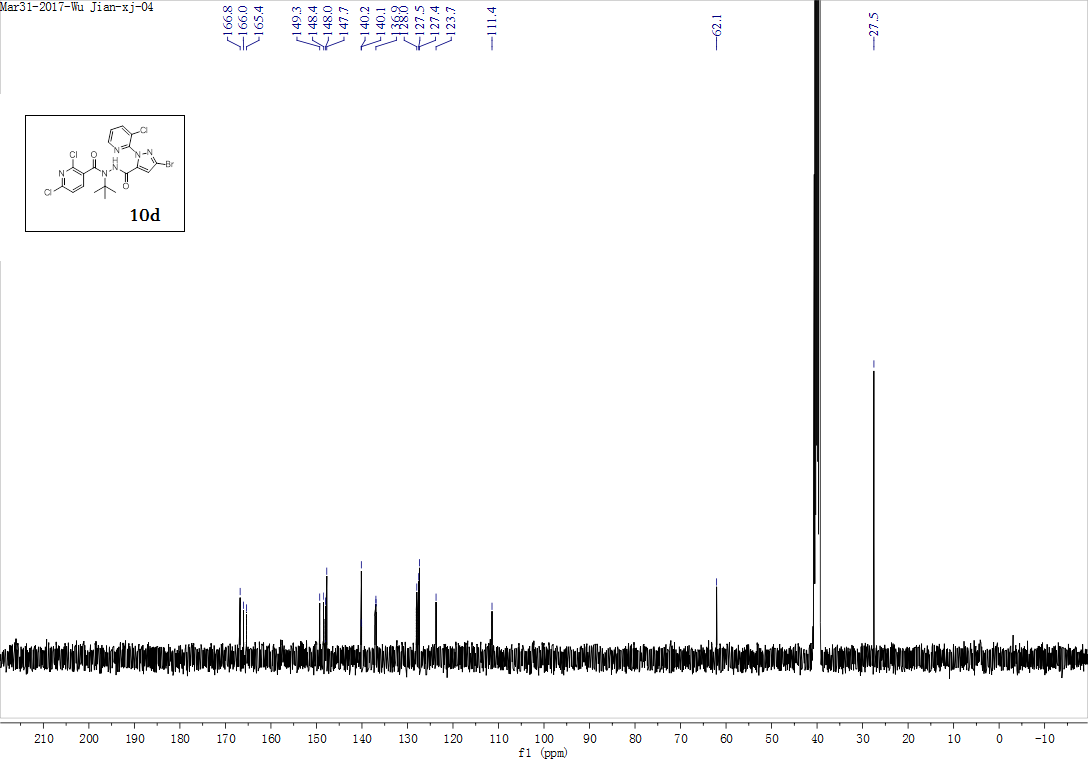


**Fig. S9** The copy of ^13^C NMR for compound **10d**


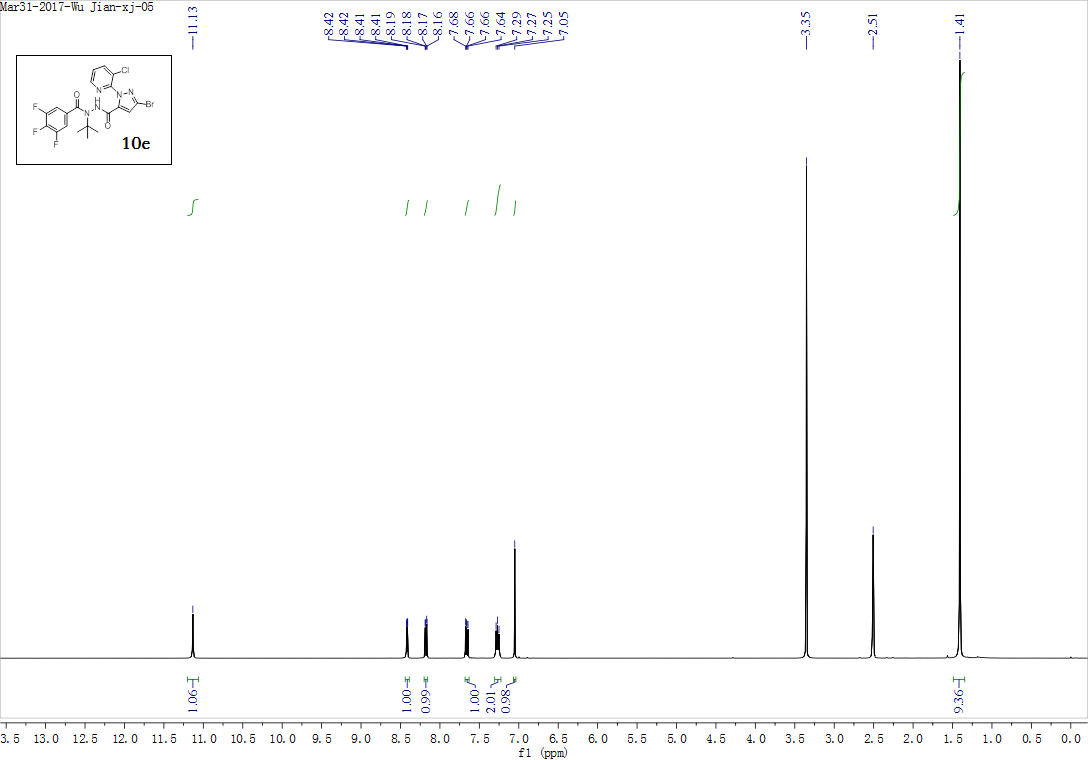


**Fig. S10** The copy of ^1^H NMR for compound **10e**


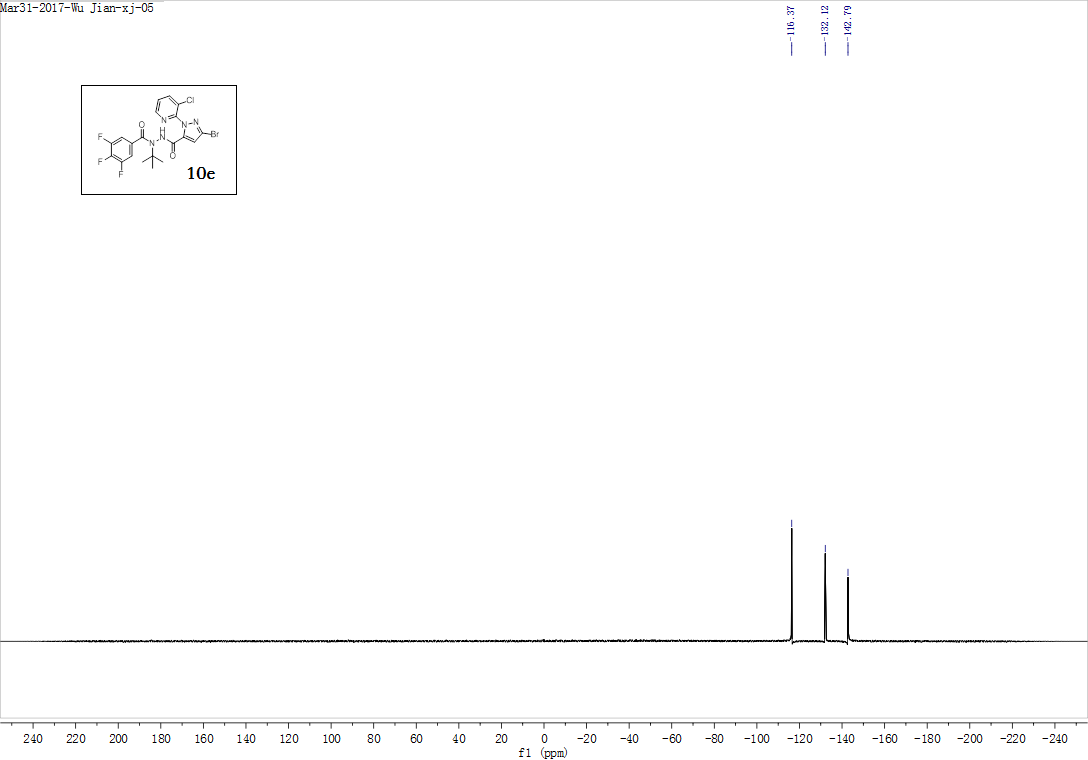


**Fig. S11** The copy of ^19^F NMR for compound **10e**


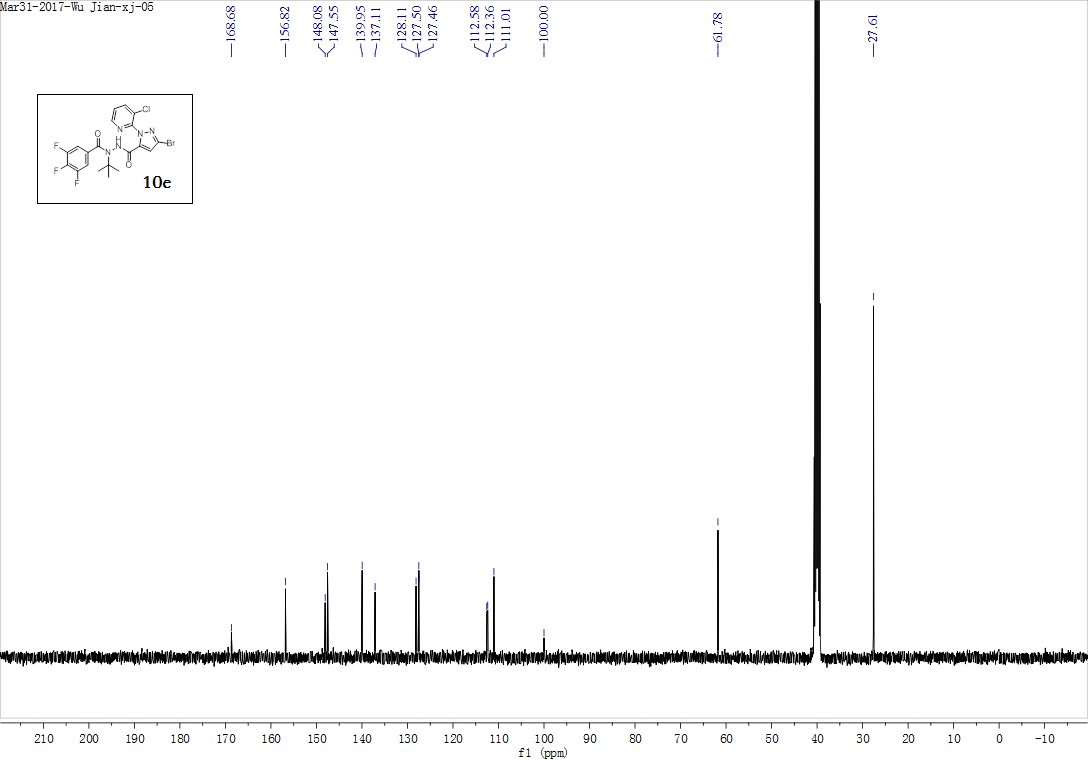


**Fig. S12** The copy of ^13^C NMR for compound **10e**


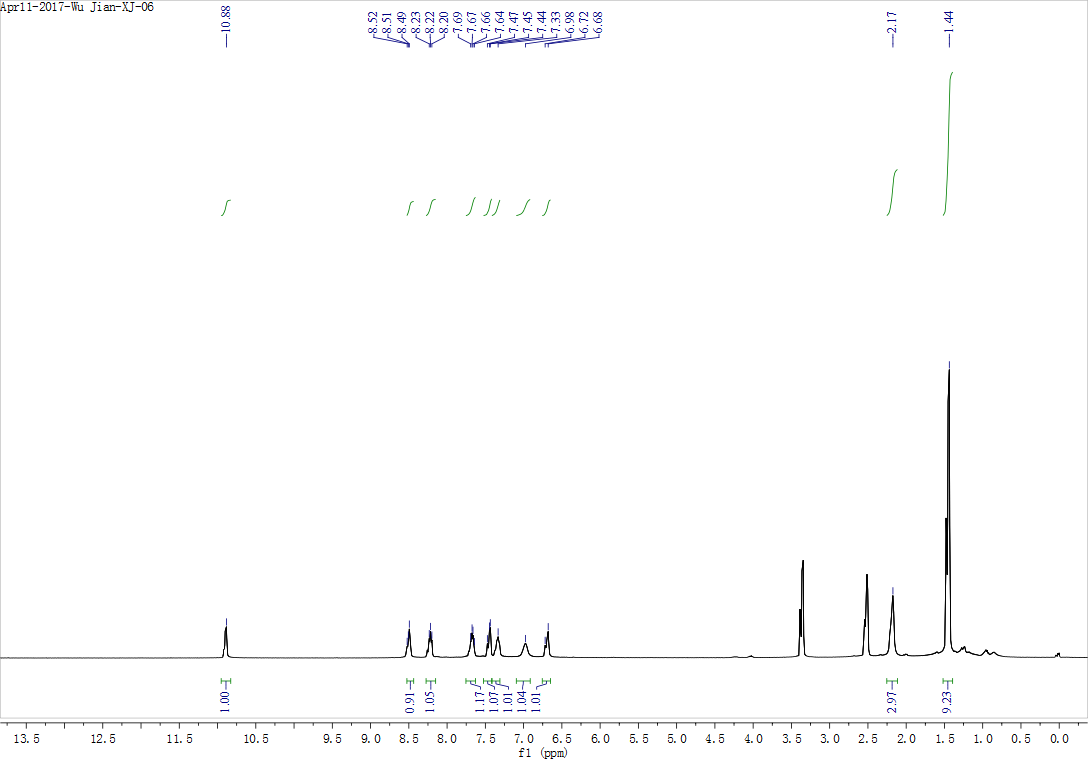


**Fig. S13** The copy of ^1^H NMR for compound **10f**


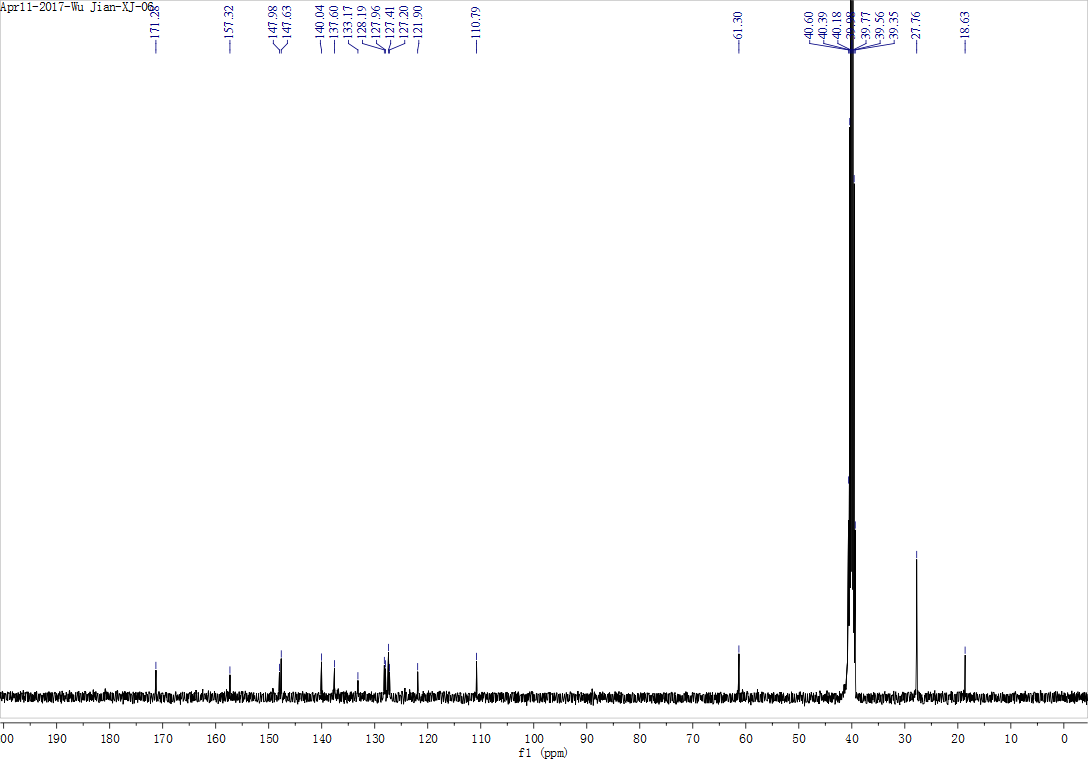


**Fig. S14** The copy of ^13^C NMR for compound **10f**


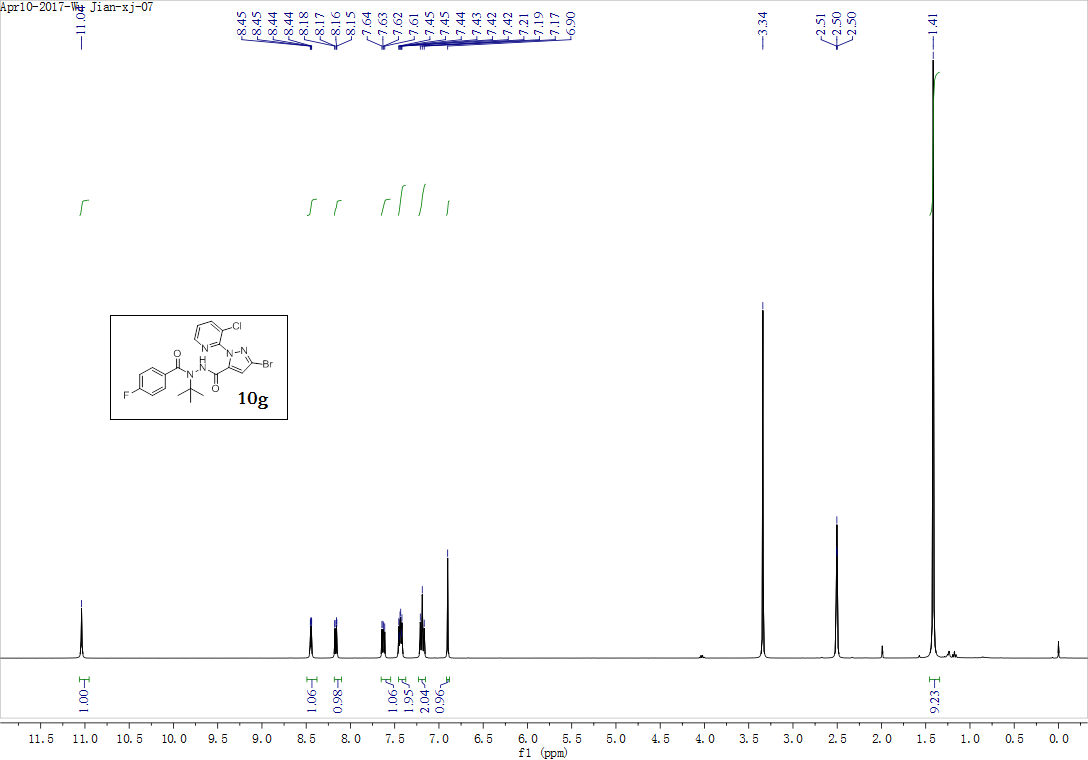


**Fig. S15** The copy of ^1^H NMR for compound **10g**


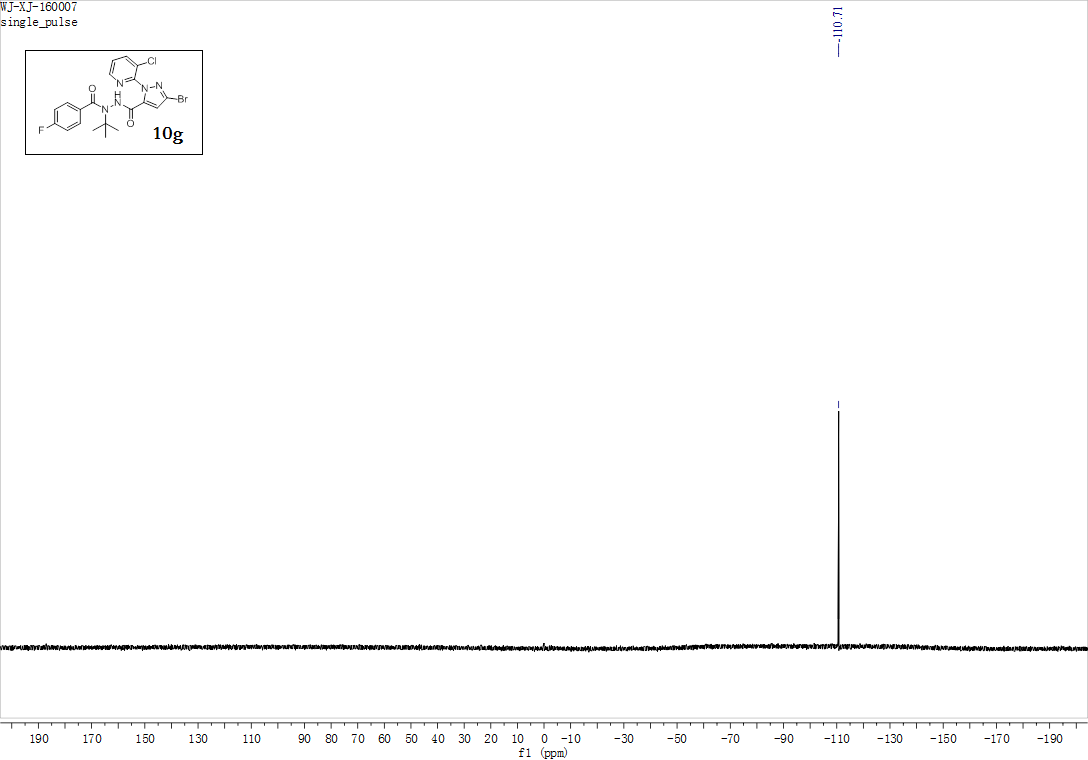


**Fig. S16** The copy of ^19^F NMR for compound **10g**


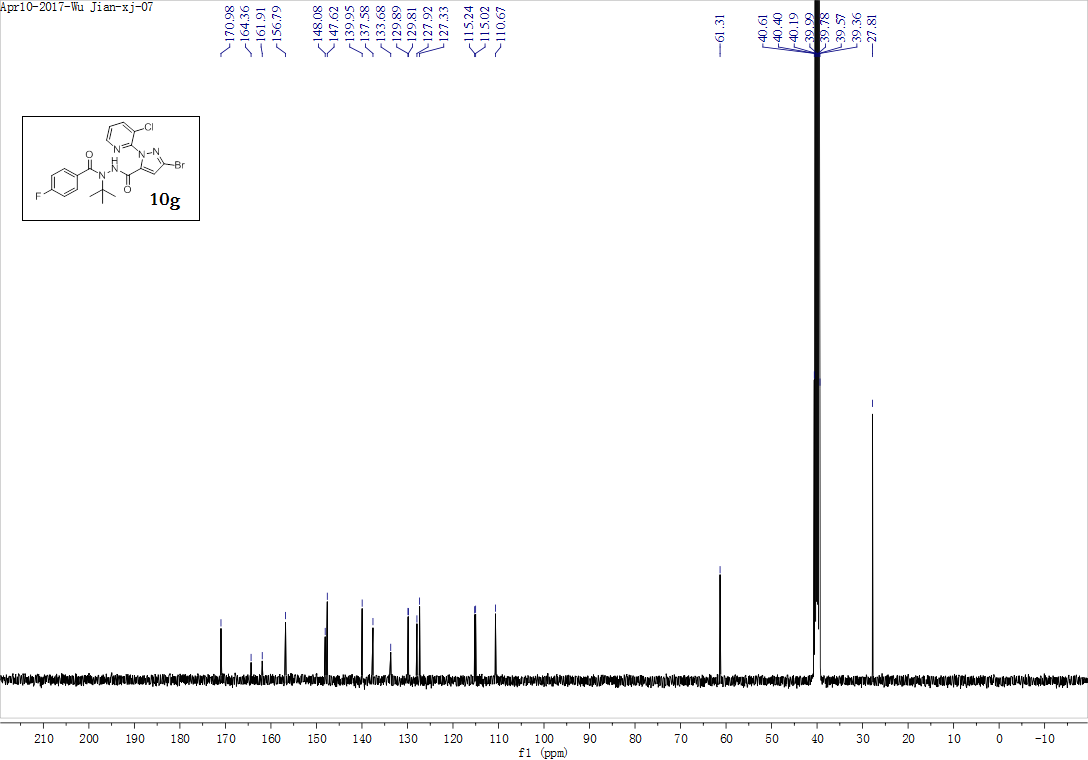


**Fig. S17** The copy of ^13^C NMR for compound **10g**


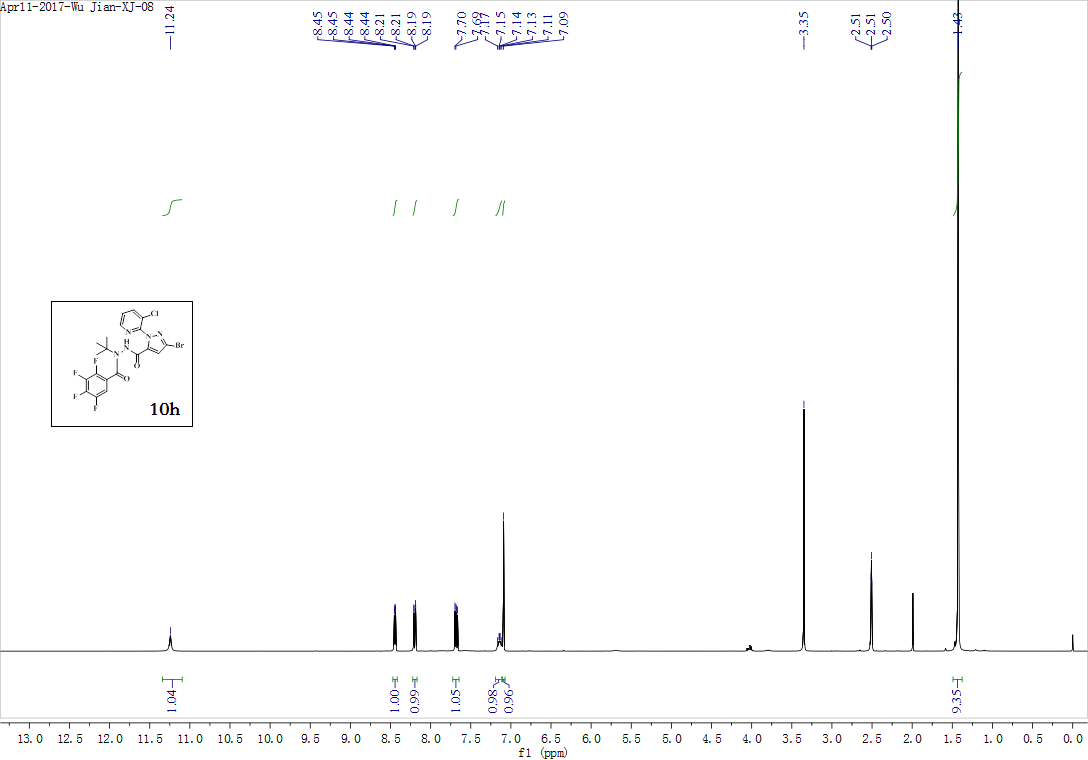


**Fig. S18** The copy of ^1^H NMR for compound **10h**


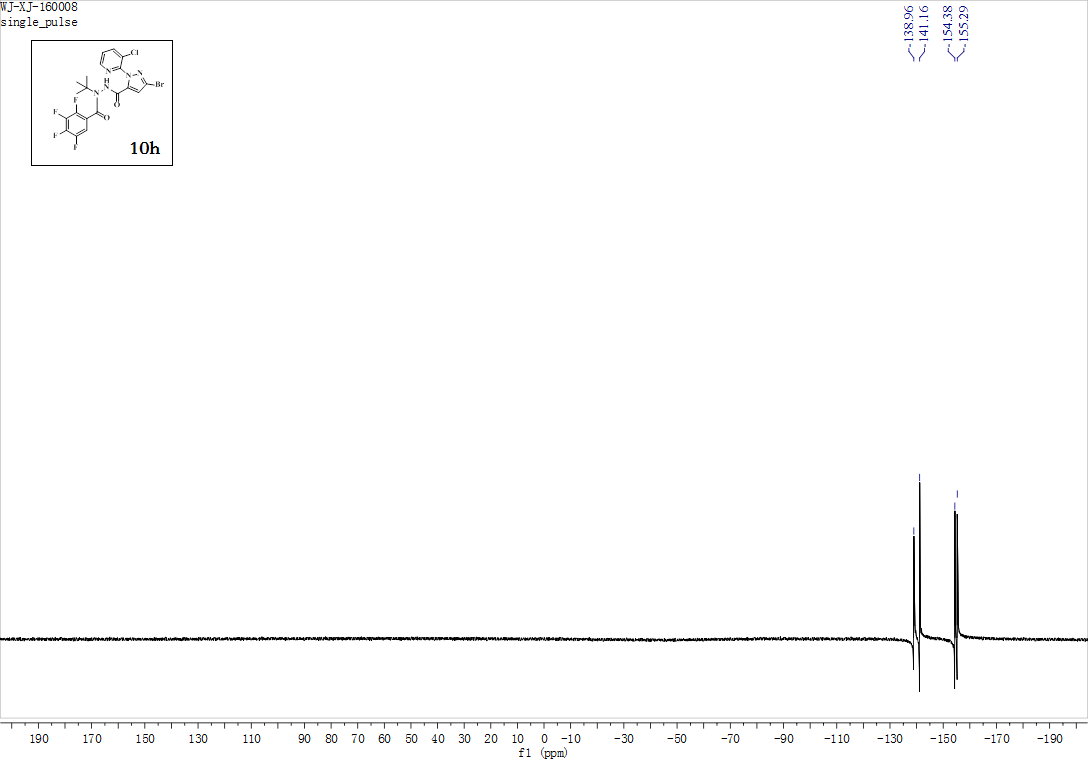


**Fig. S19** The copy of ^19^F NMR for compound **10h**


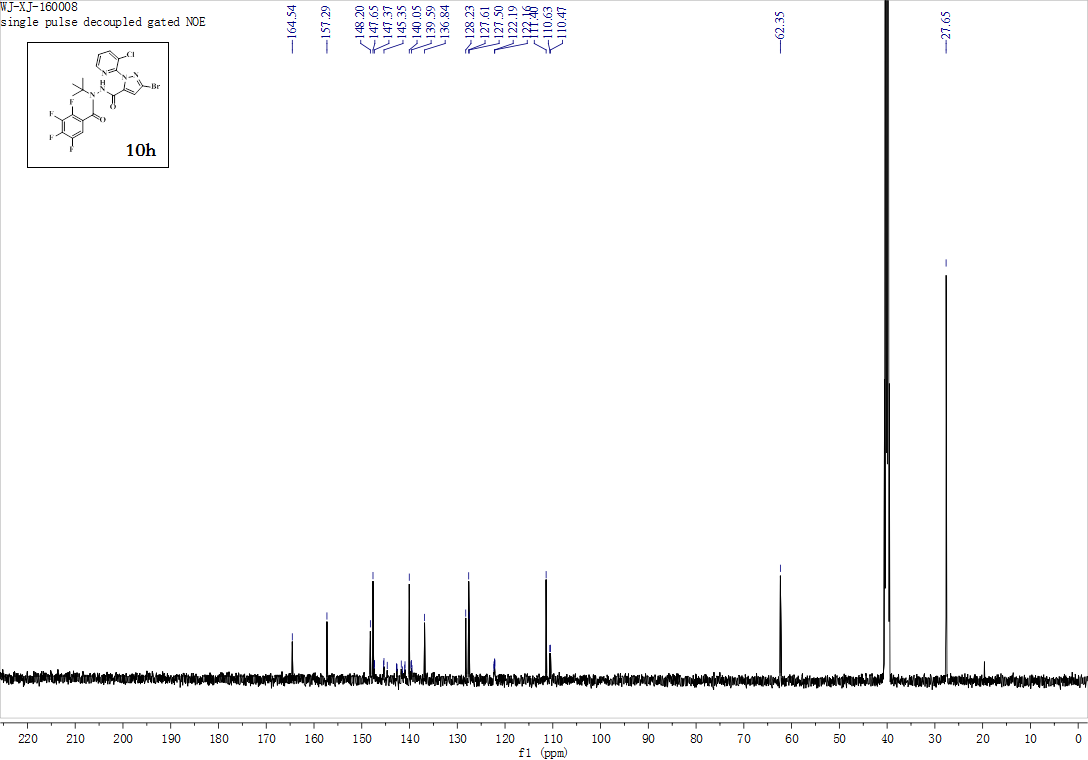


**Fig. S20** The copy of ^13^C NMR for compound **10h**


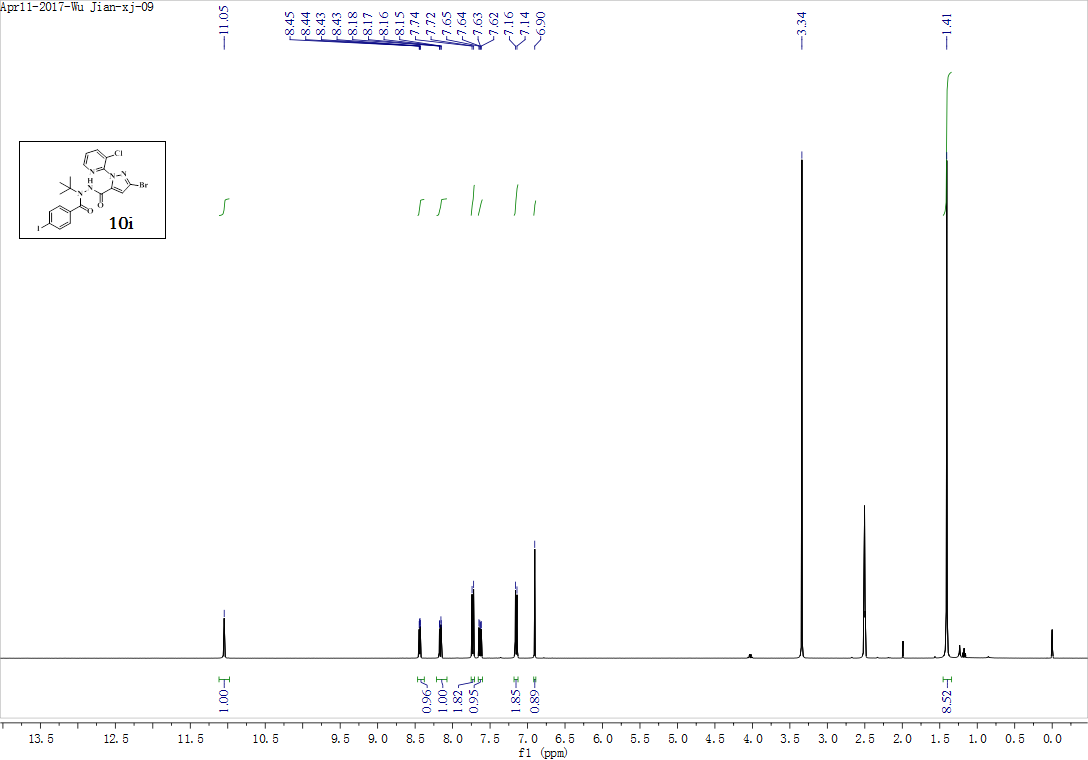


**Fig. S21** The copy of ^1^H NMR for compound **10i**


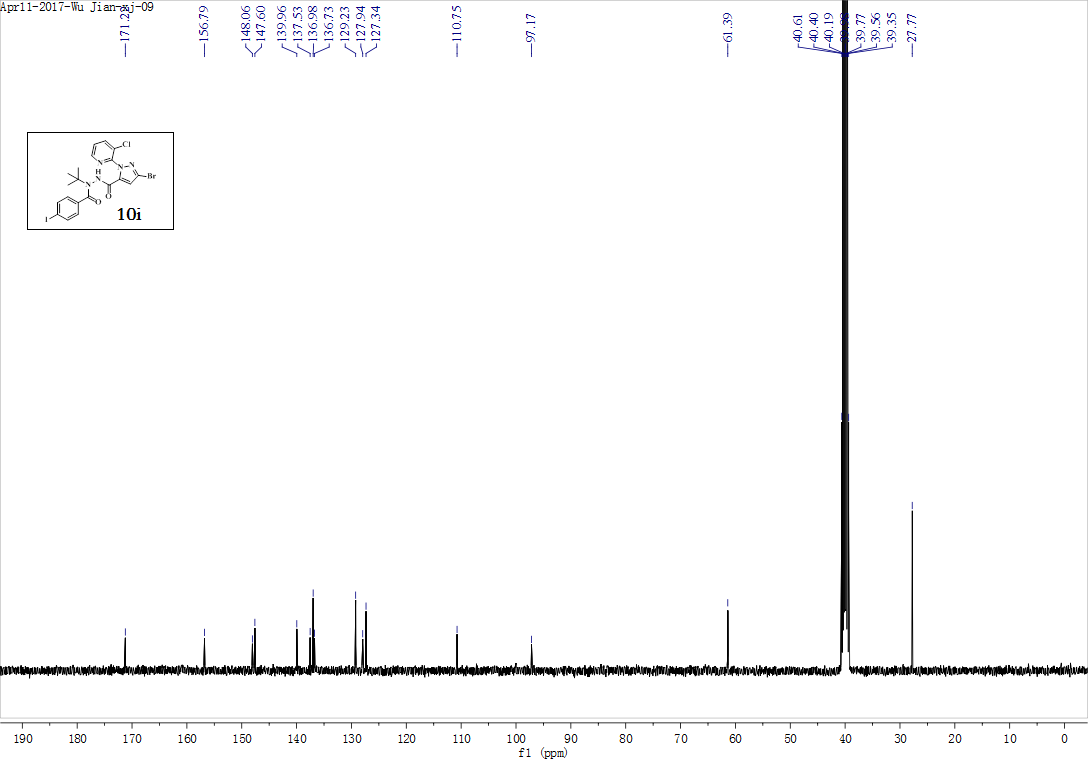


**Fig. S23** The copy of ^13^C NMR for compound **10i**


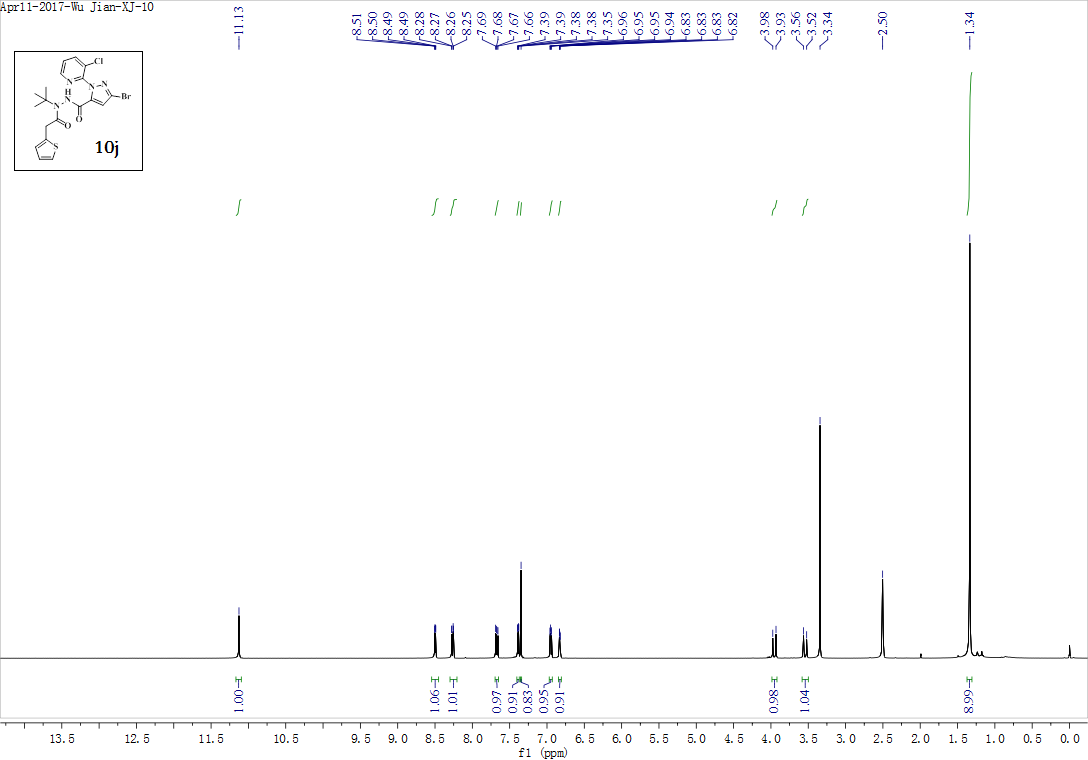


**Fig. S24** The copy of ^1^H NMR for compound **10j**


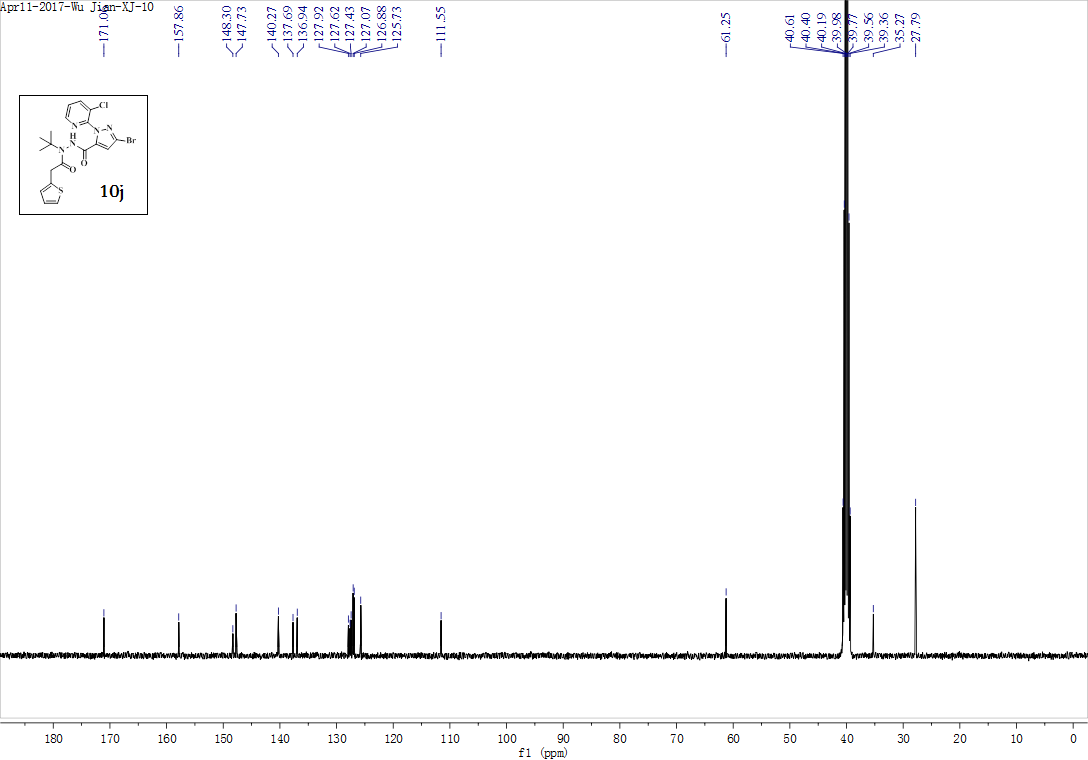


**Fig. S25** The copy of ^13^C NMR for compound **10j**


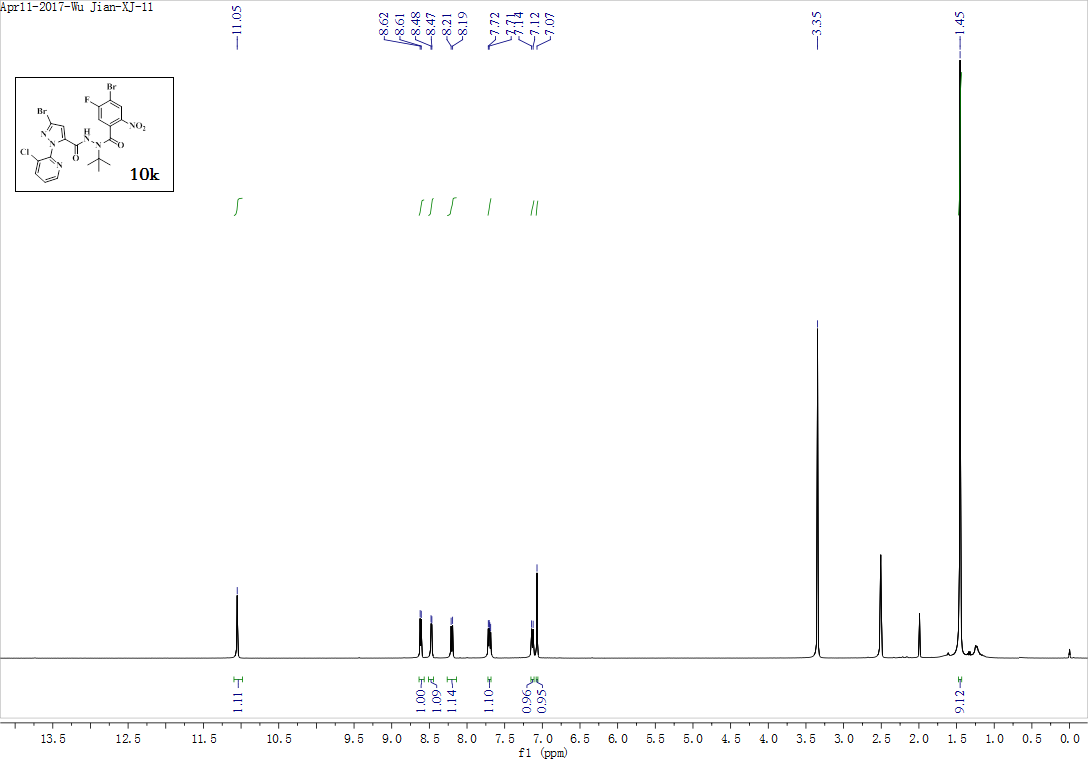


**Fig. S26** The copy of ^1^H NMR for compound **10k**


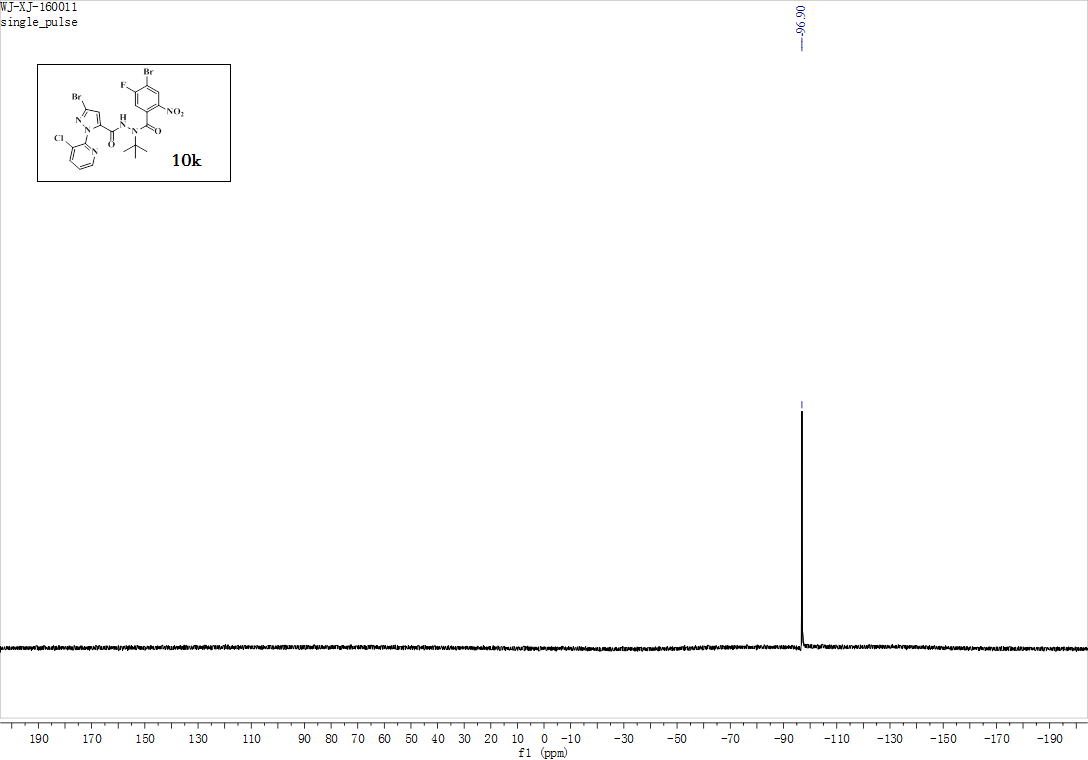


**Fig. S27** The copy of ^19^F NMR for compound **10k**


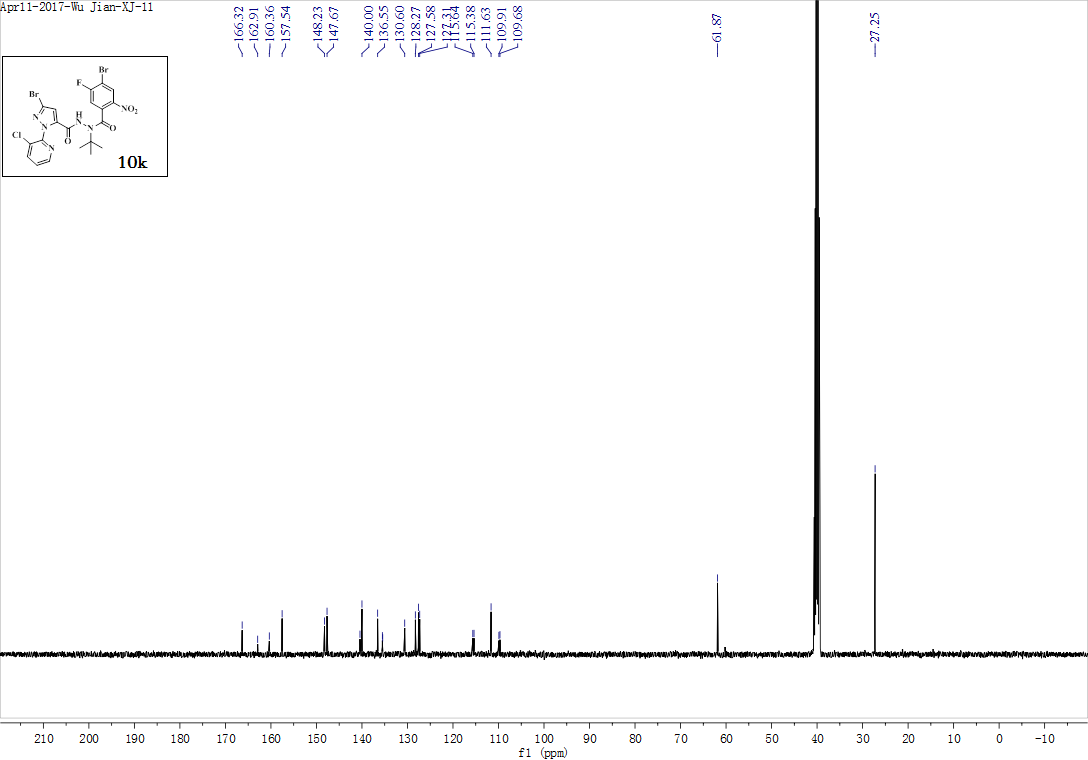


**Fig. S28** The copy of ^13^C NMR for compound **10k**


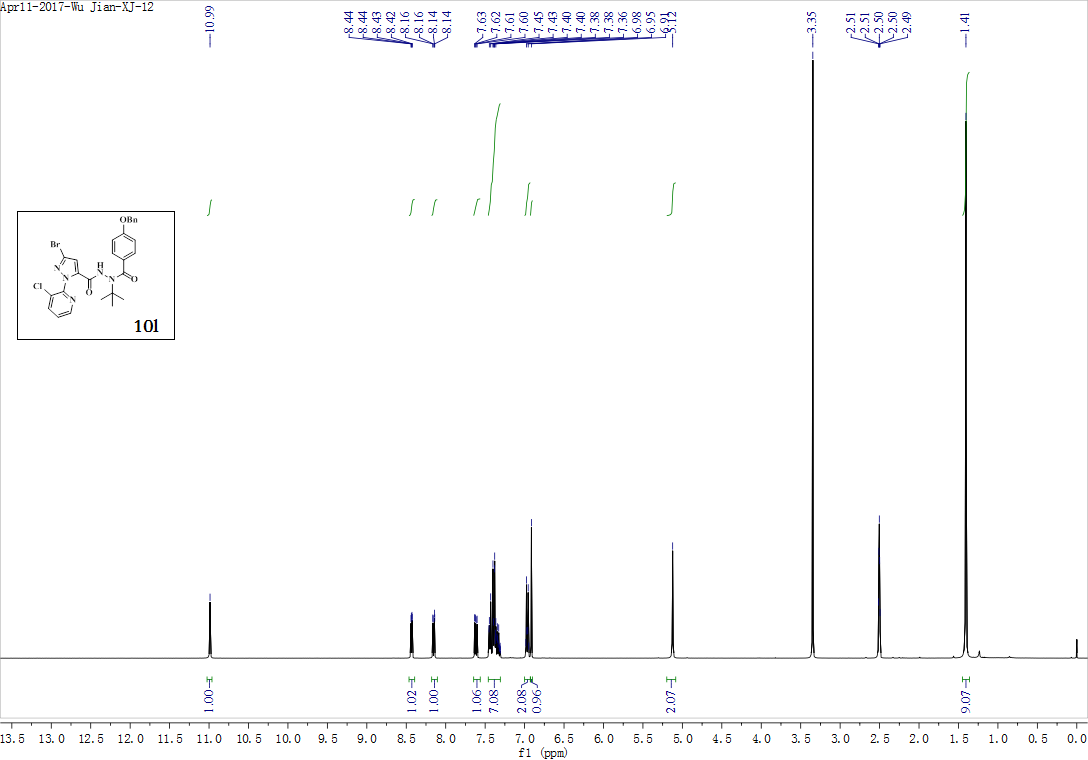


**Fig. S29** The copy of ^1^H NMR for compound **10l**


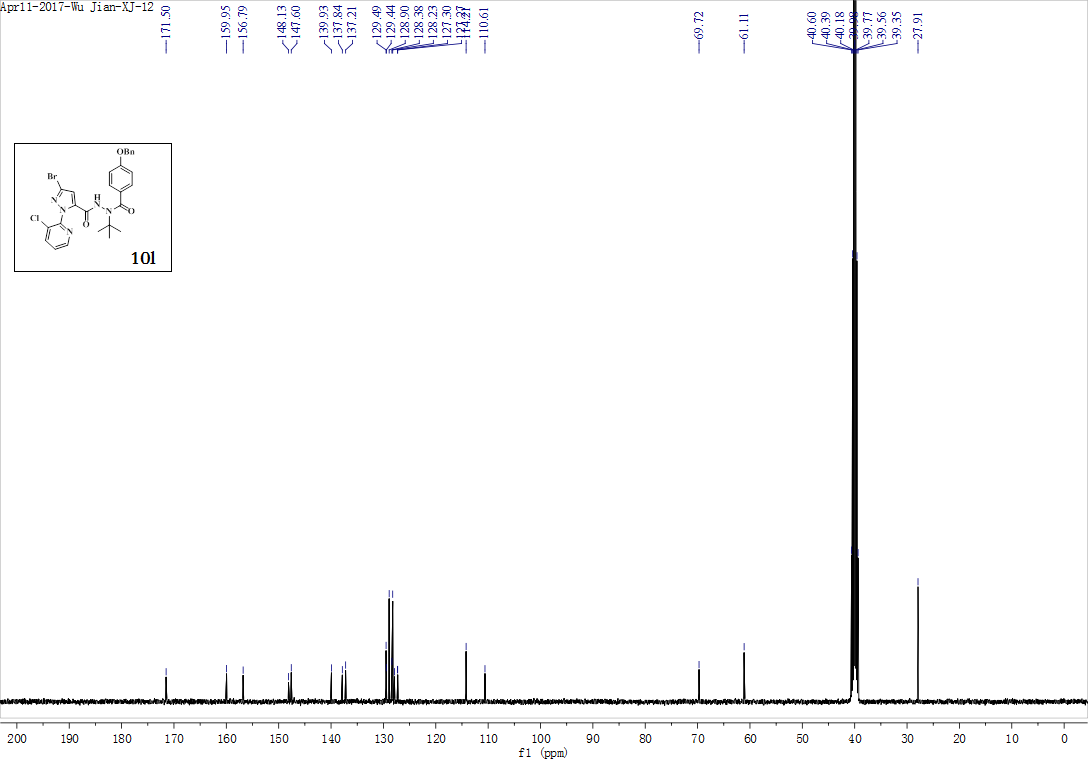


**Fig. S30** The copy of ^13^C NMR for compound **10l**


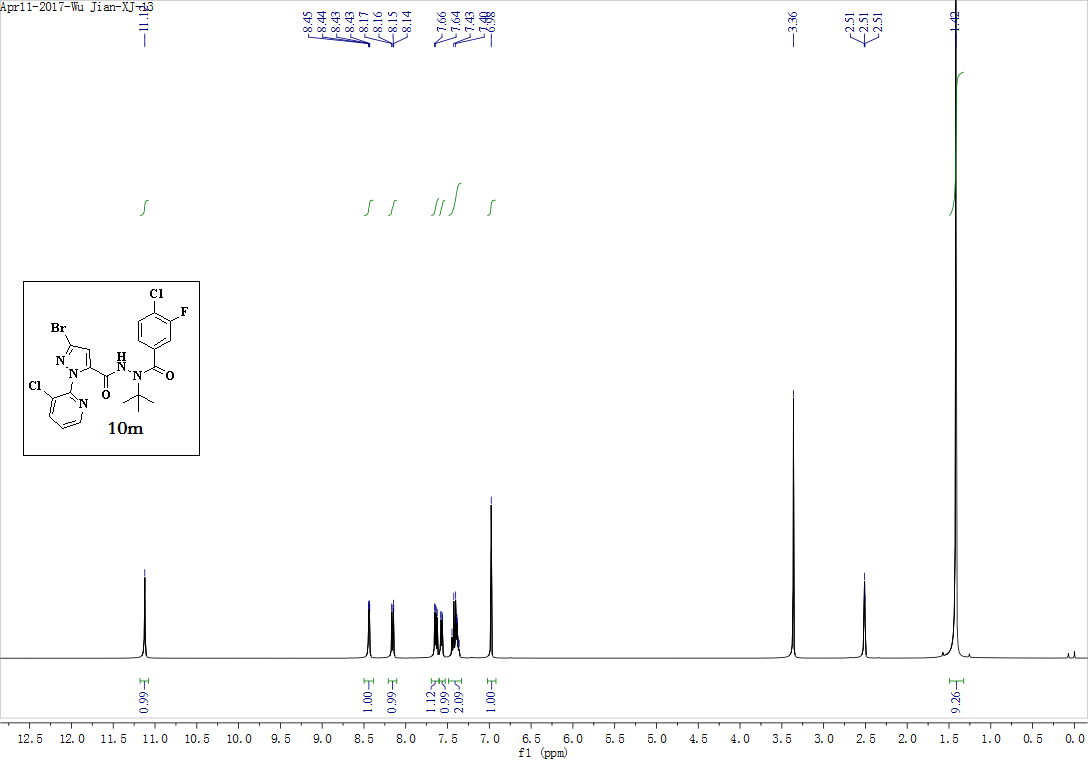


**Fig. S31** The copy of ^1^H NMR for compound **10m**


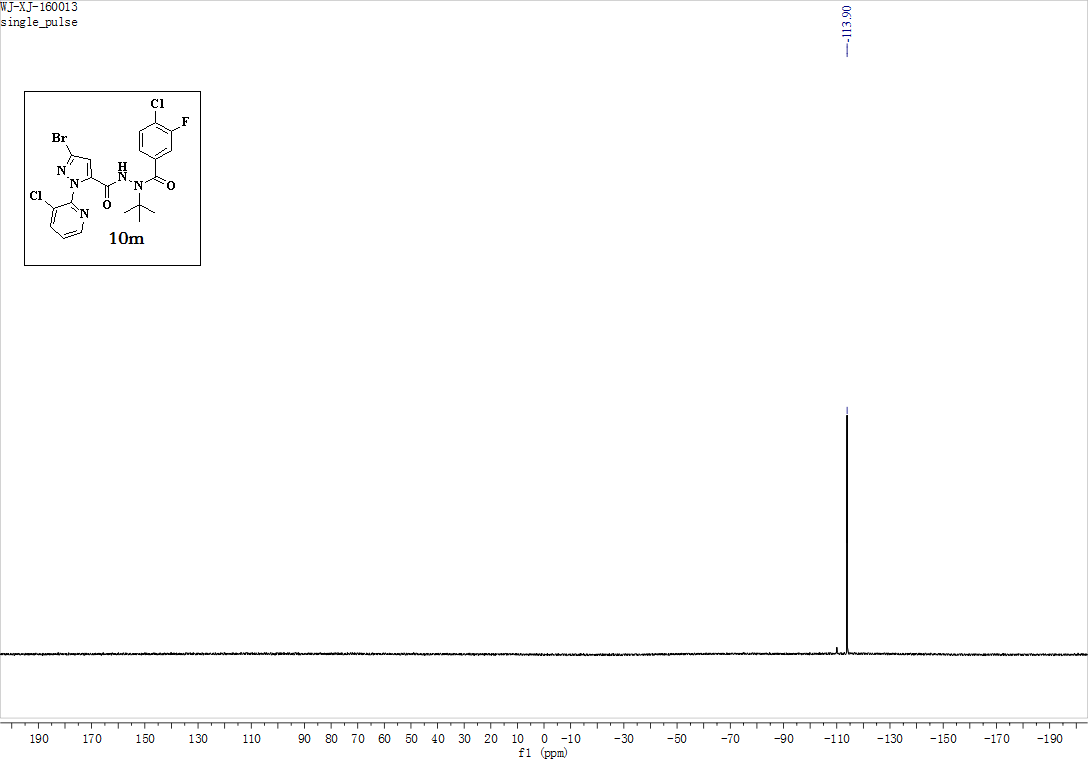


**Fig. S32** The copy of ^19^F NMR for compound **10m**


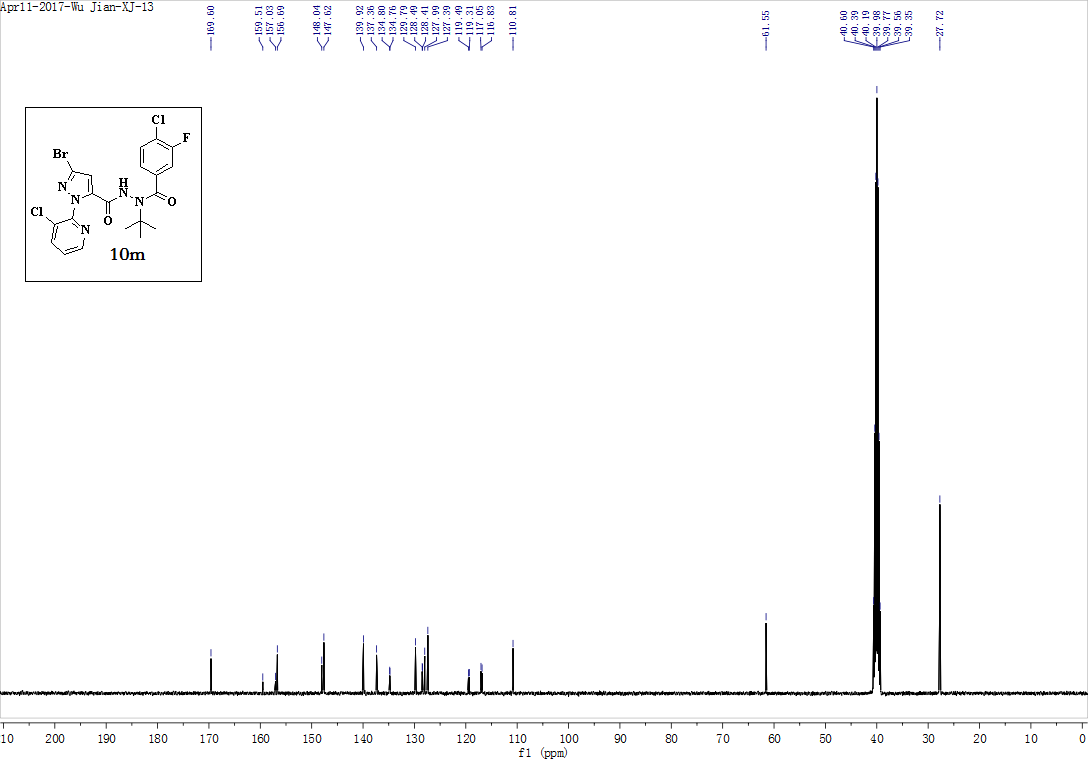


**Fig. S33** The copy of ^13^C NMR for compound **10m**


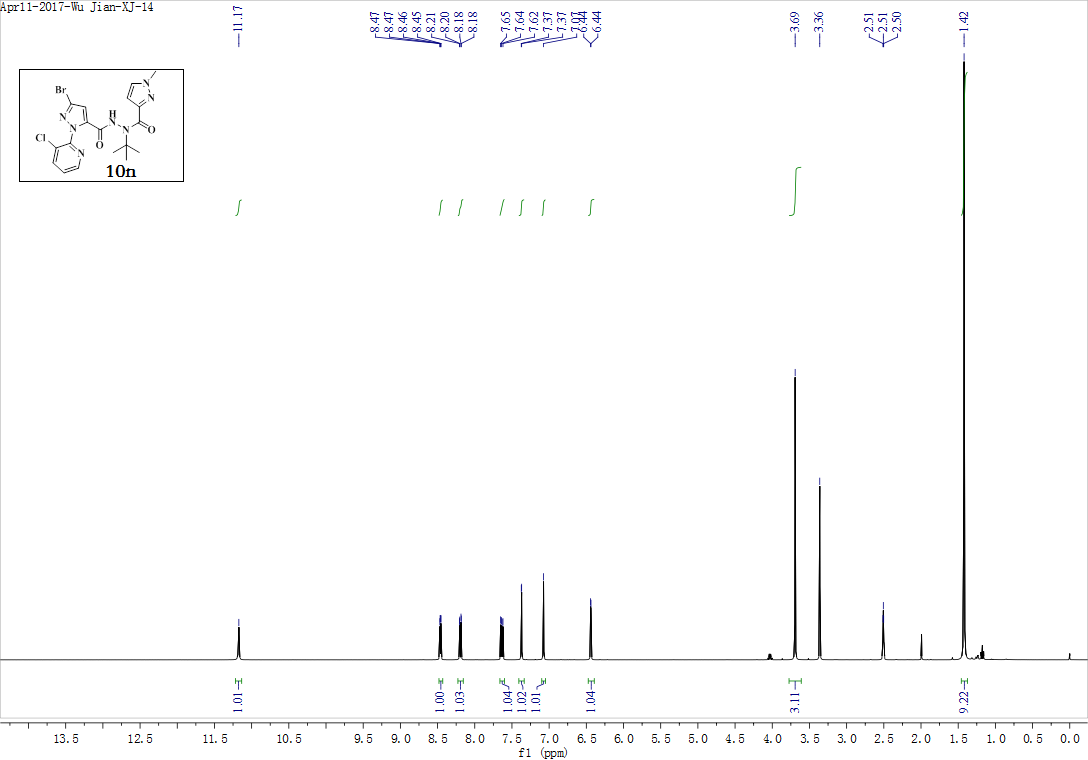


**Fig. S34** The copy of ^1^H NMR for compound **10n**


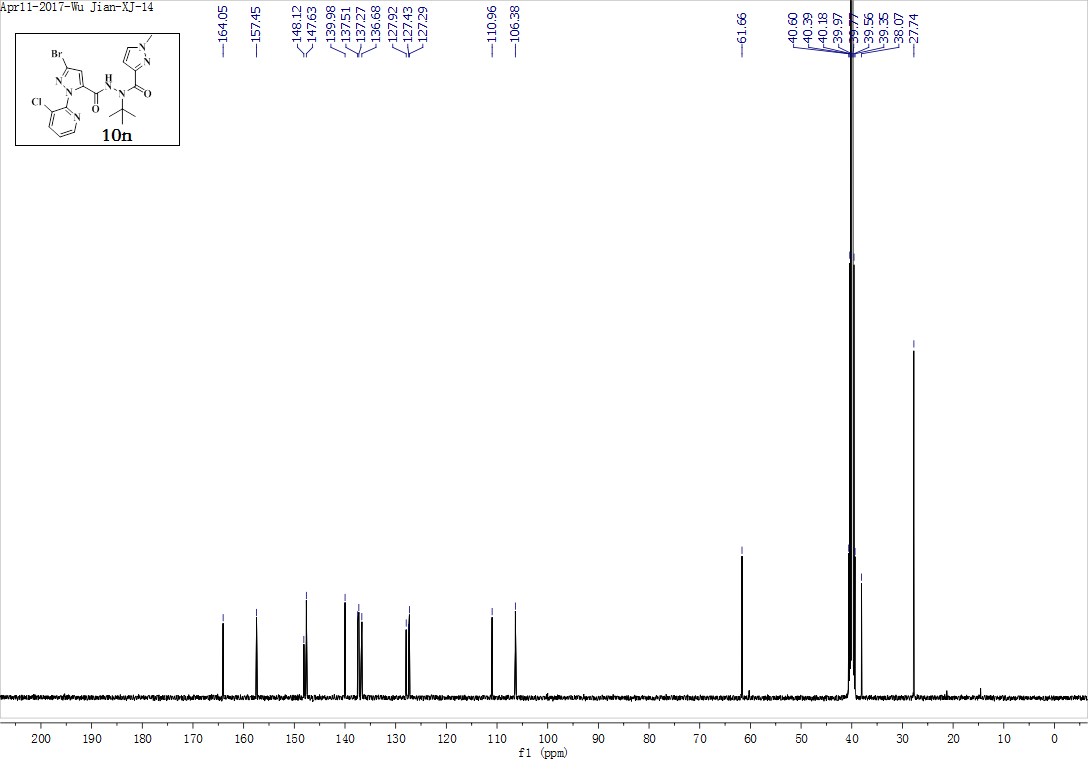


**Fig. S35** The copy of ^13^C NMR for compound **10n**


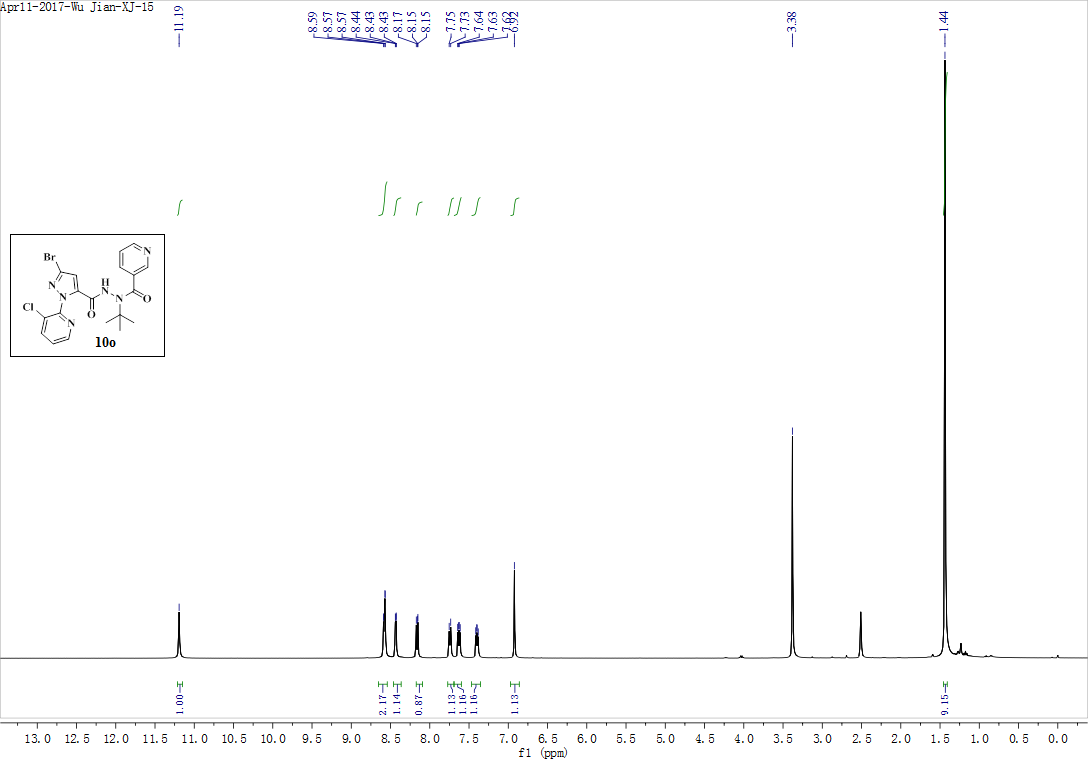


**Fig. S36** The copy of ^1^H NMR for compound **10o**


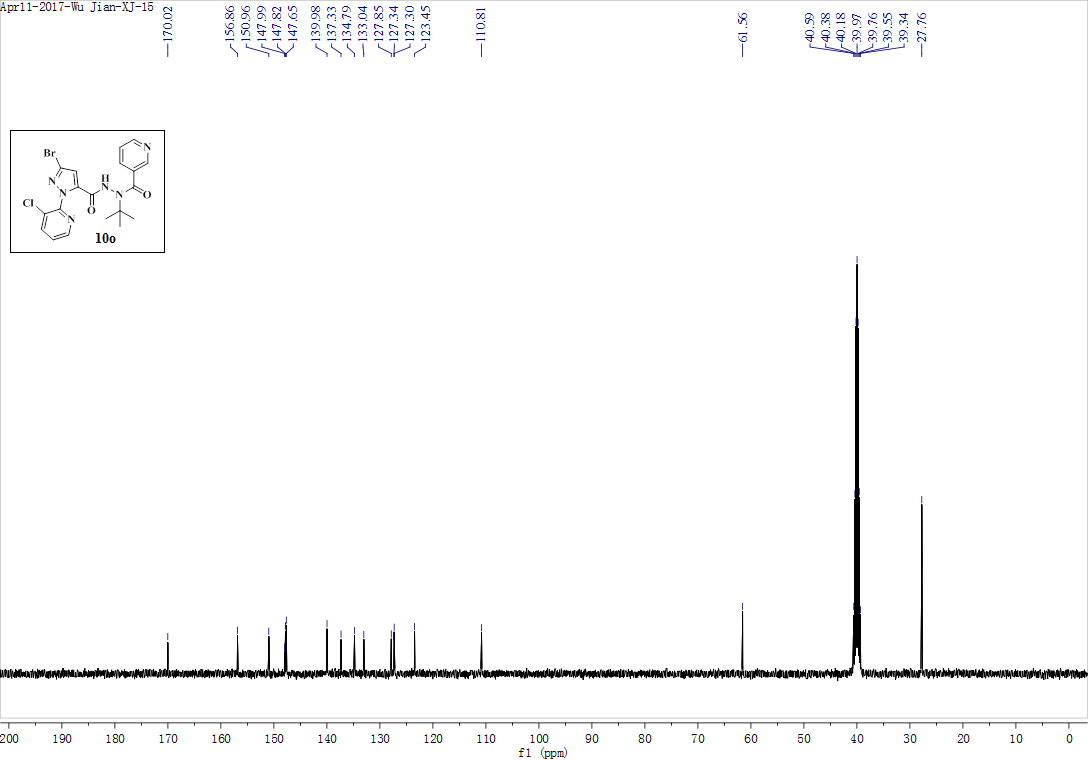


**Fig. S37** The copy of ^13^C NMR for compound **10o**


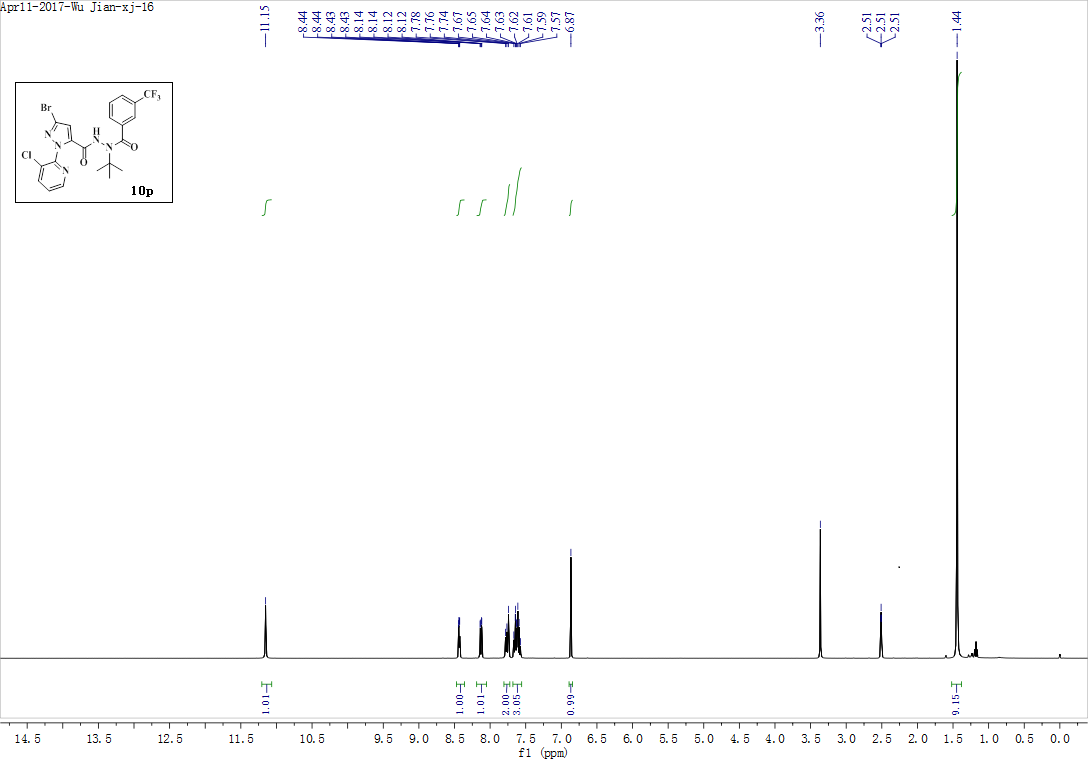


**Fig. S38** The copy of ^1^H NMR for compound **10p**


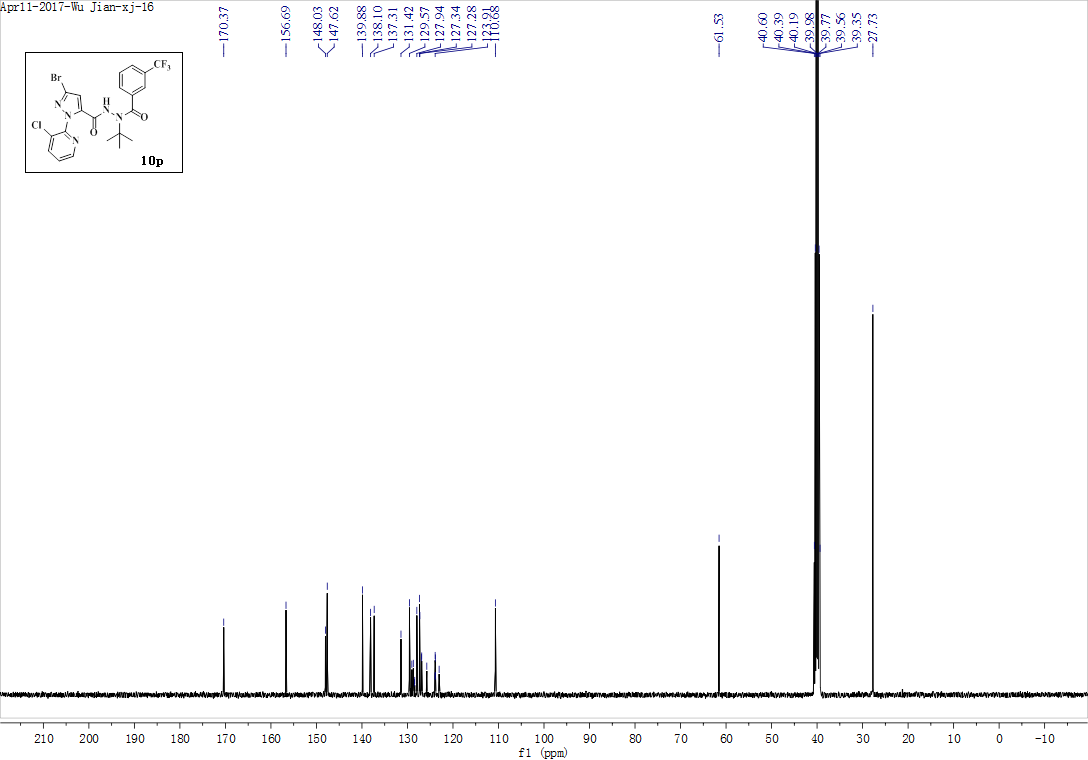


**Fig. S39** The copy of ^13^C NMR for compound 10p


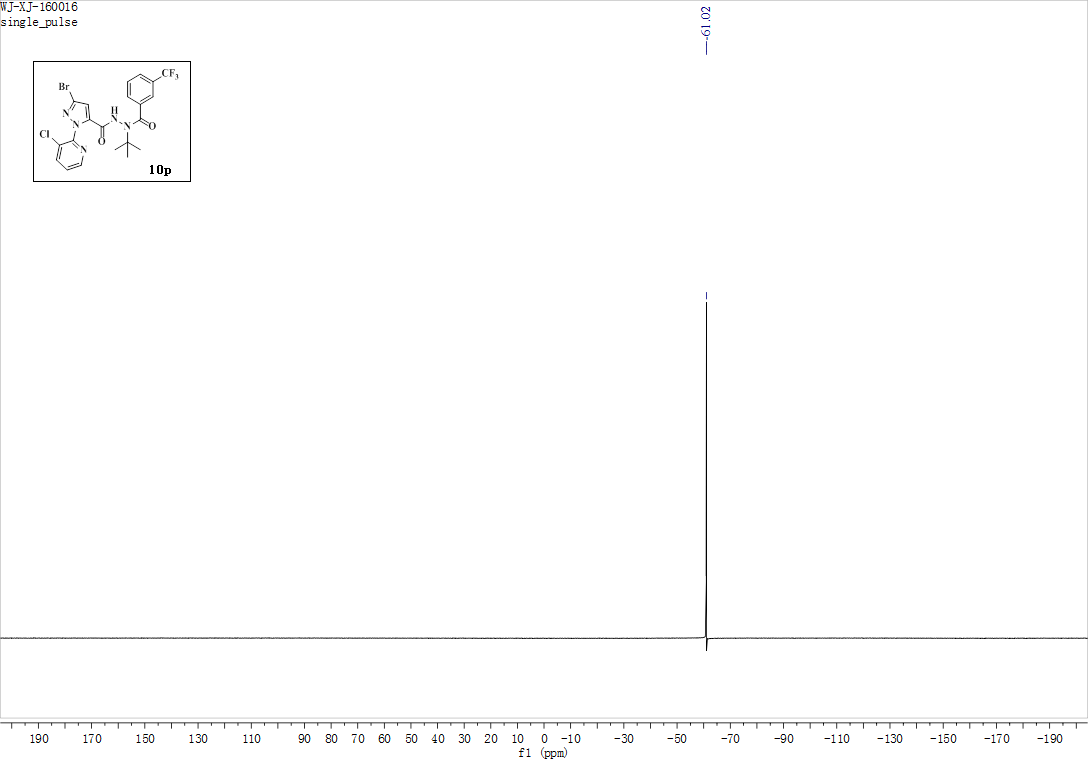


**Fig. S40** The copy of ^19^F NMR for compound **10p**


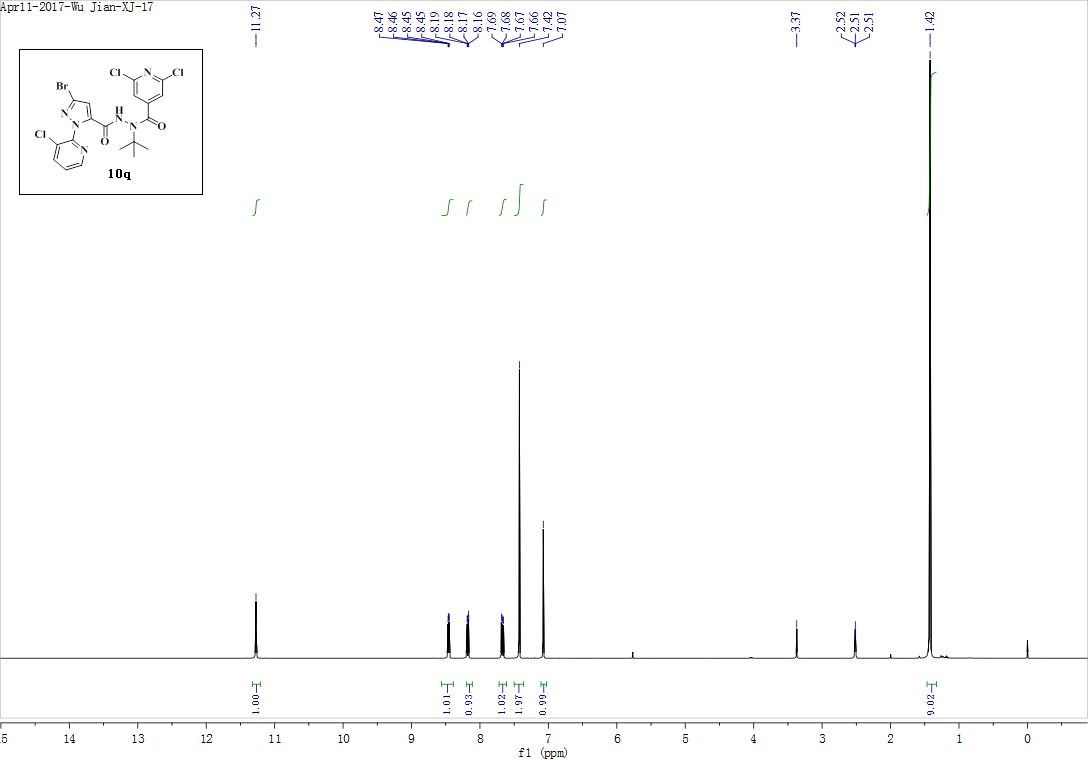


**Fig. S41** The copy of ^1^H NMR for compound **10q**


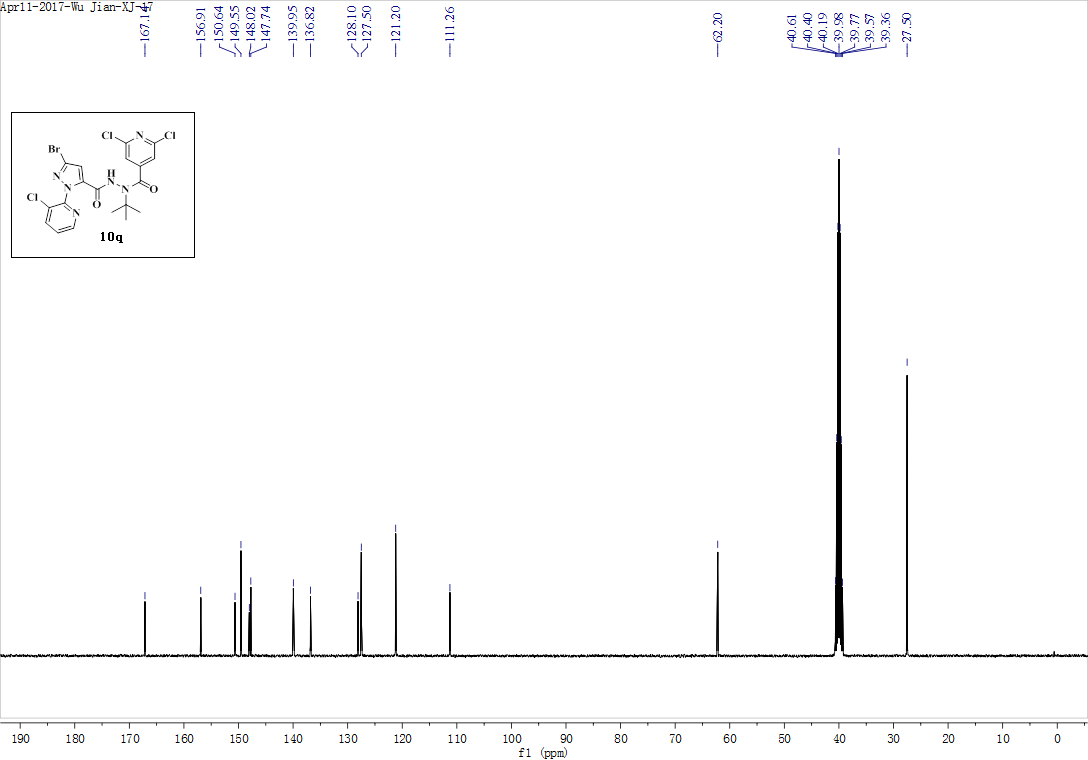


**Fig. S42** The copy of ^13^C NMR for compound **10q**


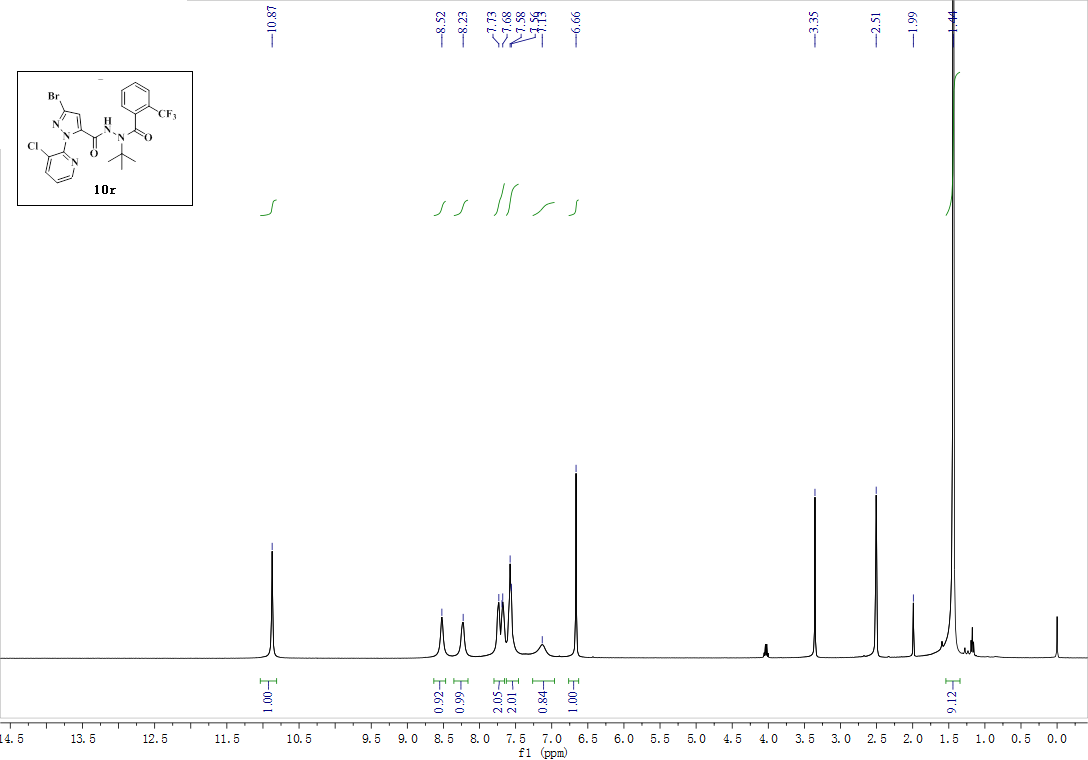


**Fig. S43** The copy of ^1^H NMR for compound **10r**


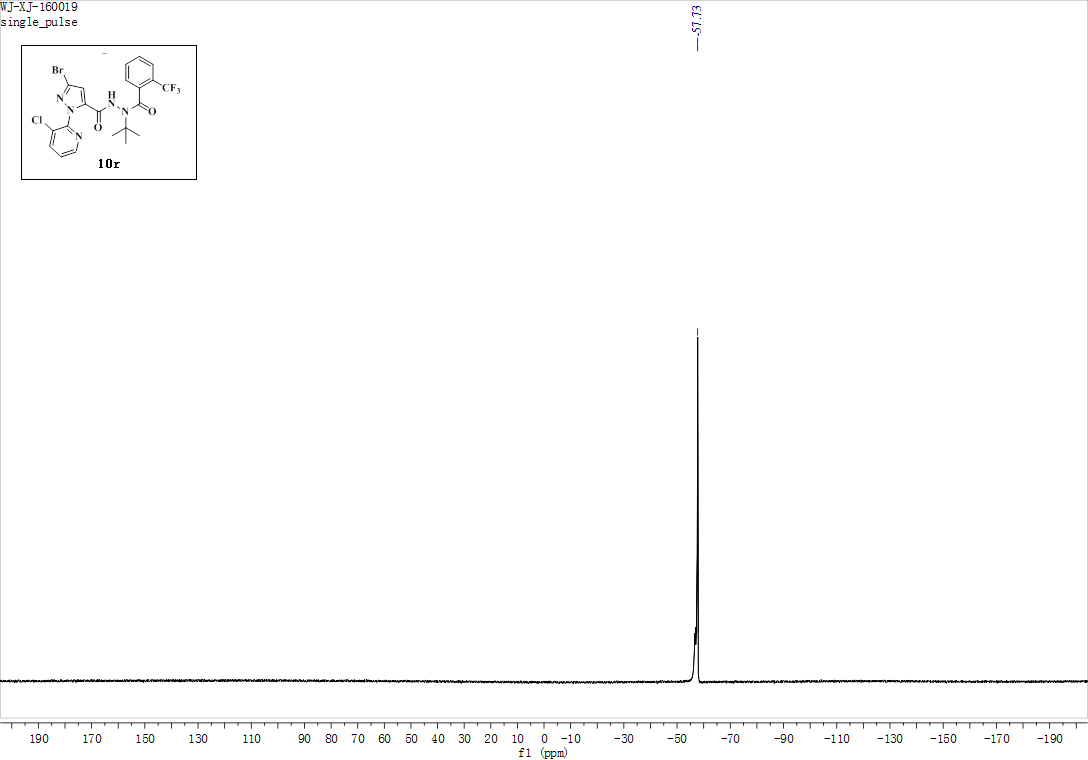


**Fig. S44** The copy of ^19^F NMR for compound **10r**


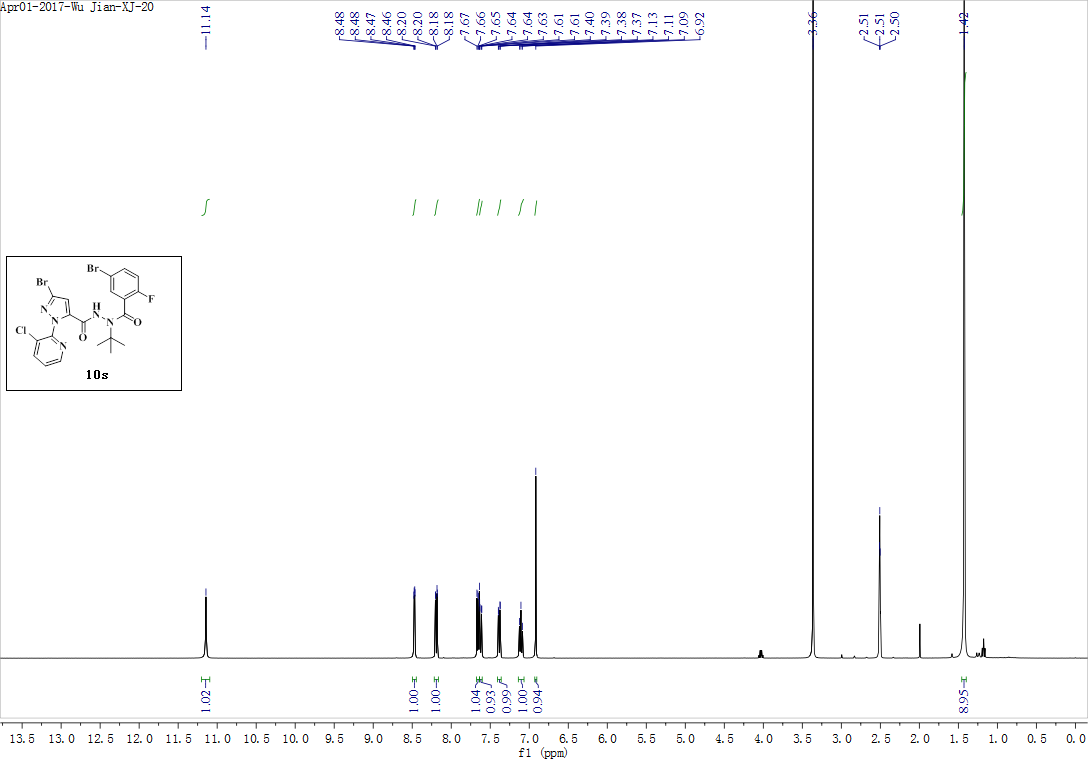


**Fig. S45** The copy of ^1^H NMR for compound 10s


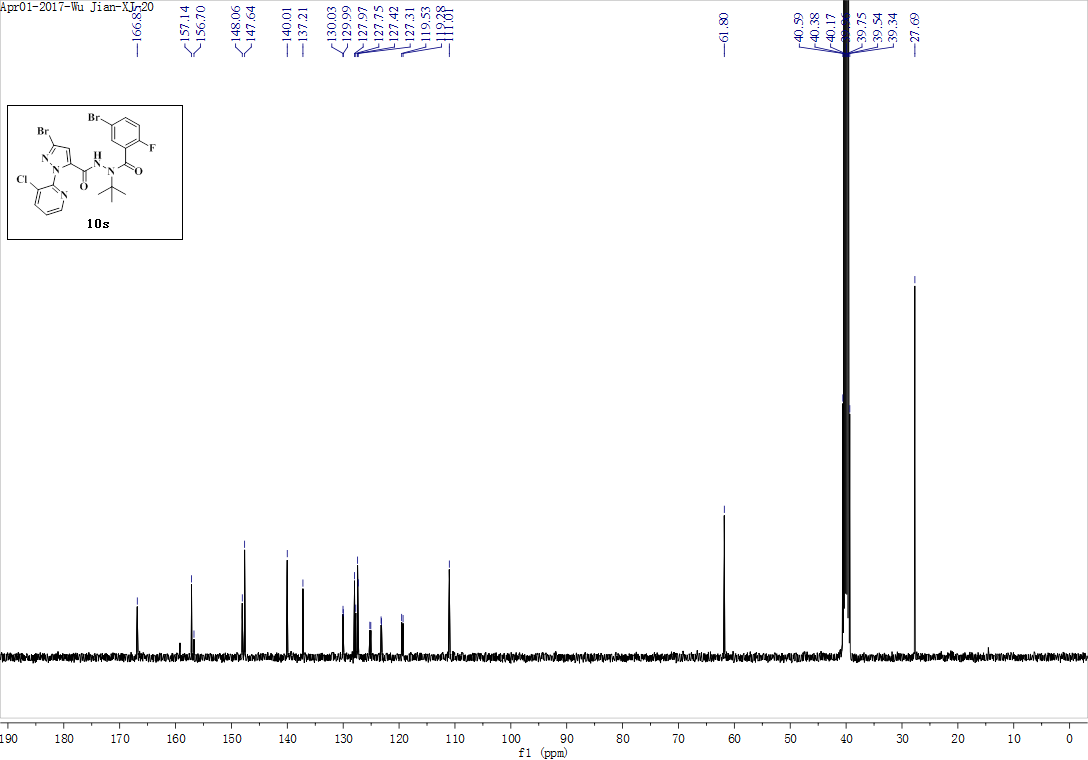


**Fig. S46** The copy of ^13^C NMR for compound **10s**


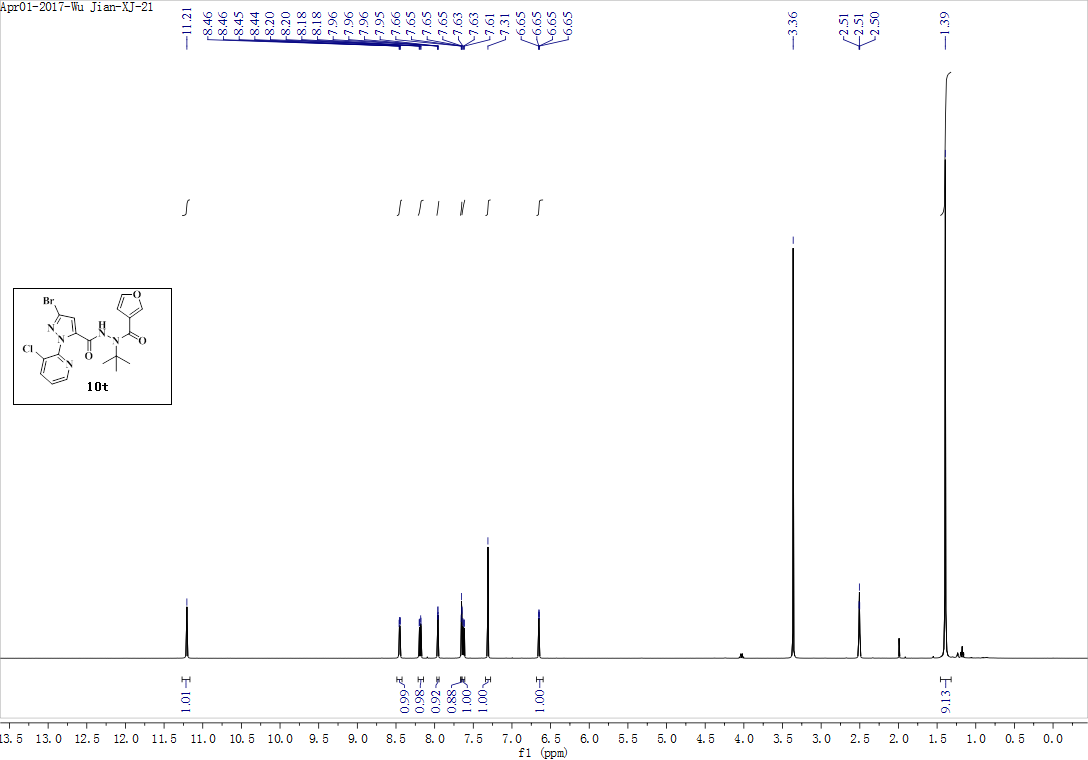


**Fig. S47** The copy of ^1^H NMR for compound **10t**


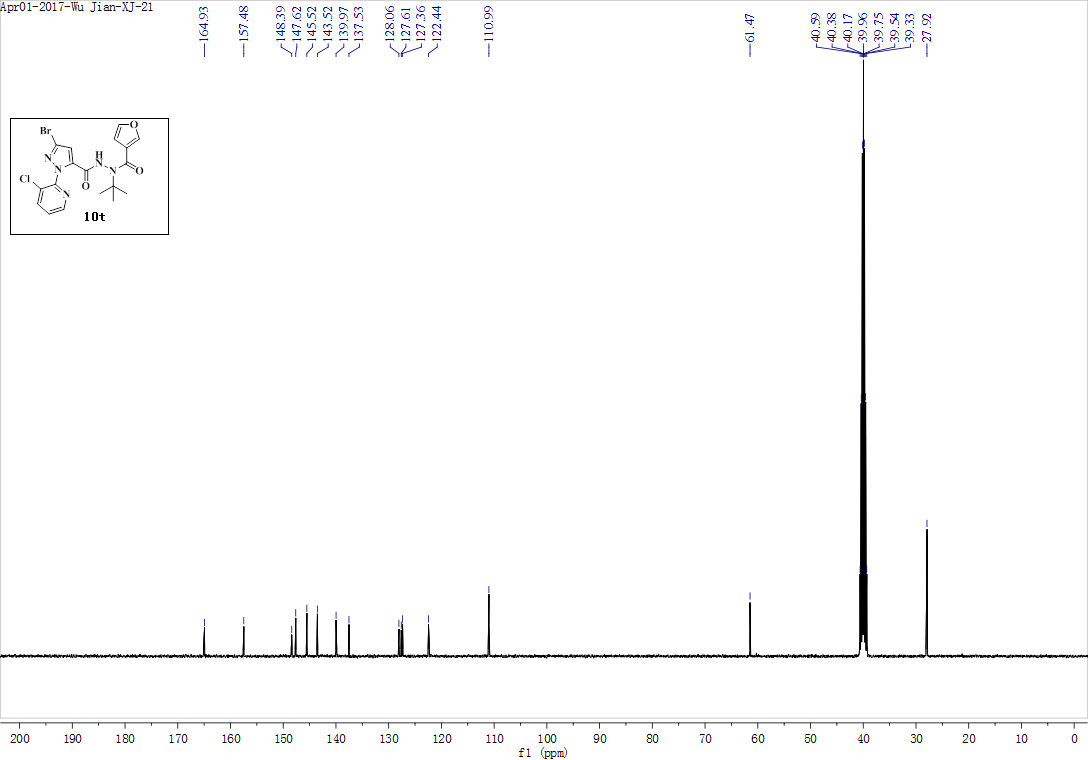


**Fig. S48** The copy of ^13^C NMR for compound **10t**


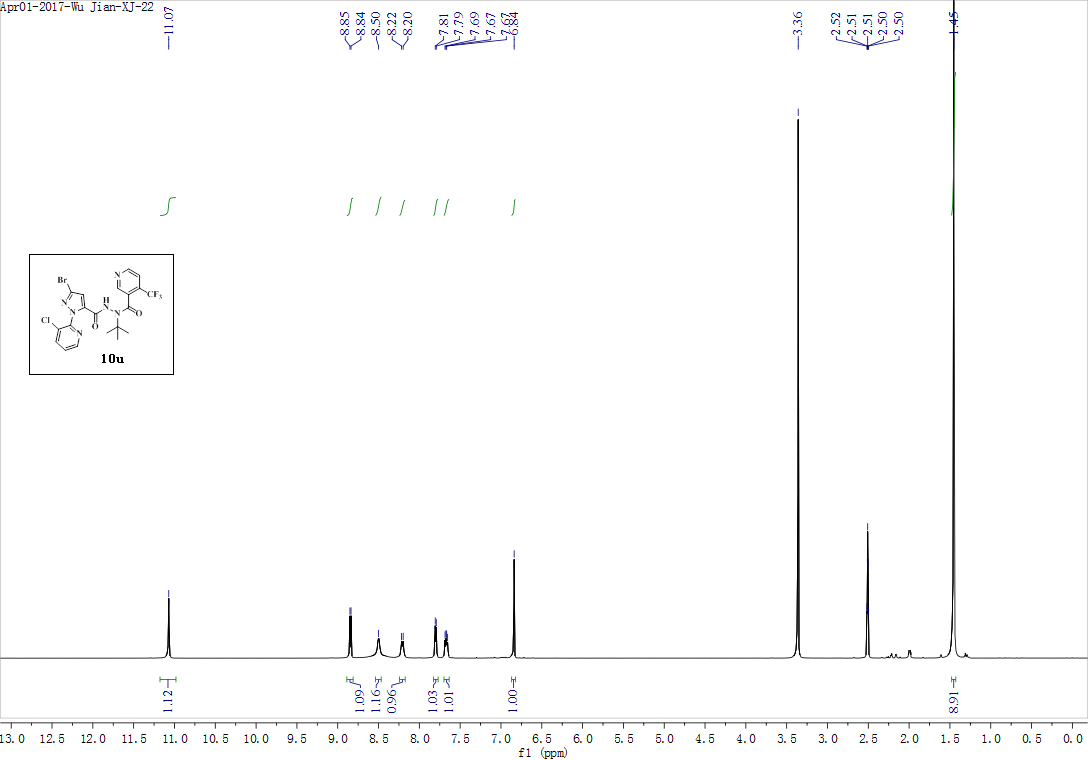


**Fig. S49** The copy of ^1^H NMR for compound **10u**


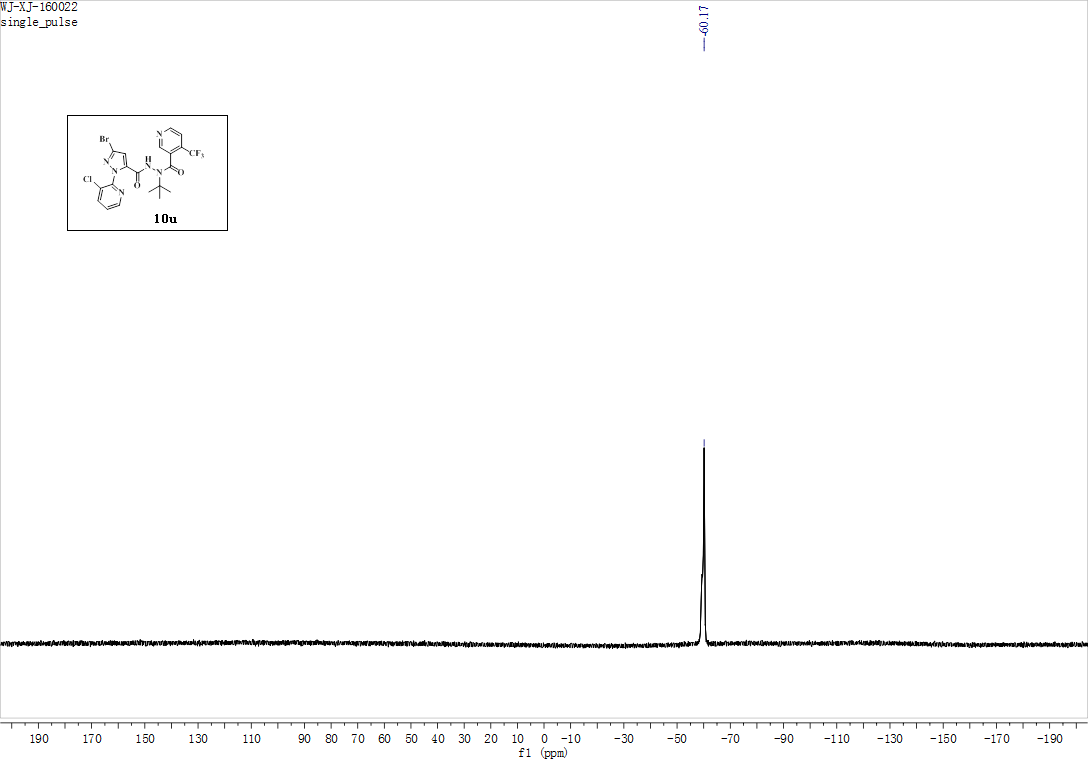


**Fig. S50** The copy of ^19^F NMR for compound **10u**


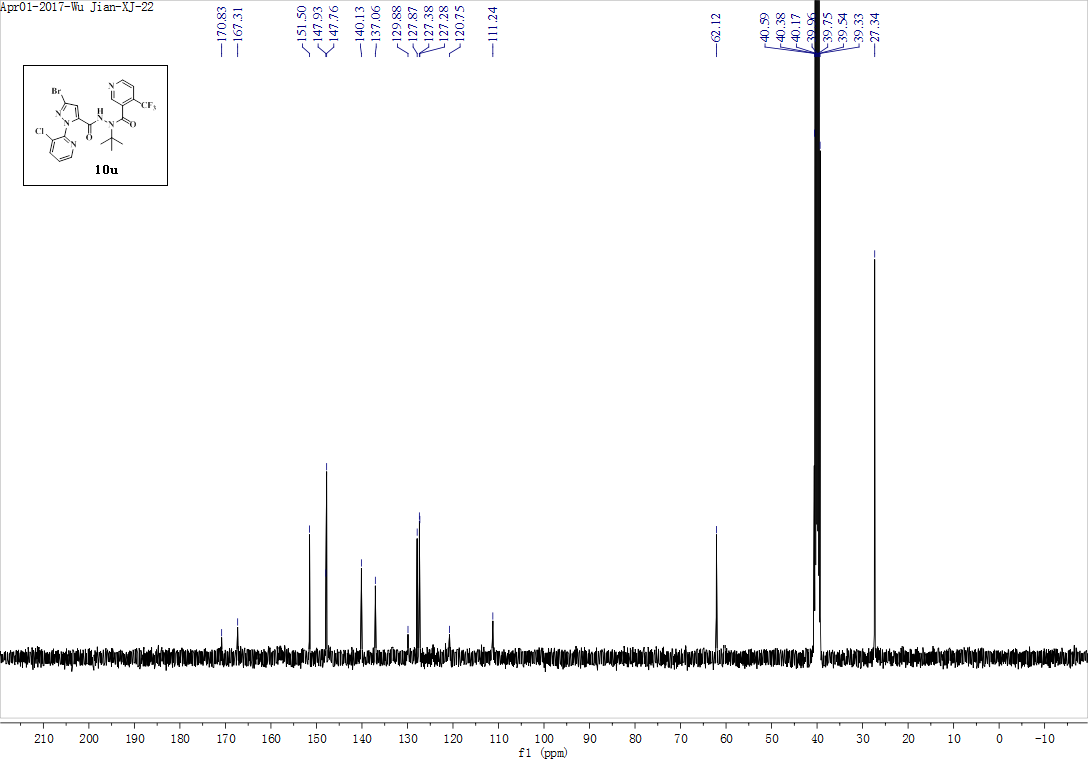


**Fig. S51** The copy of ^13^C NMR for compound **10u**


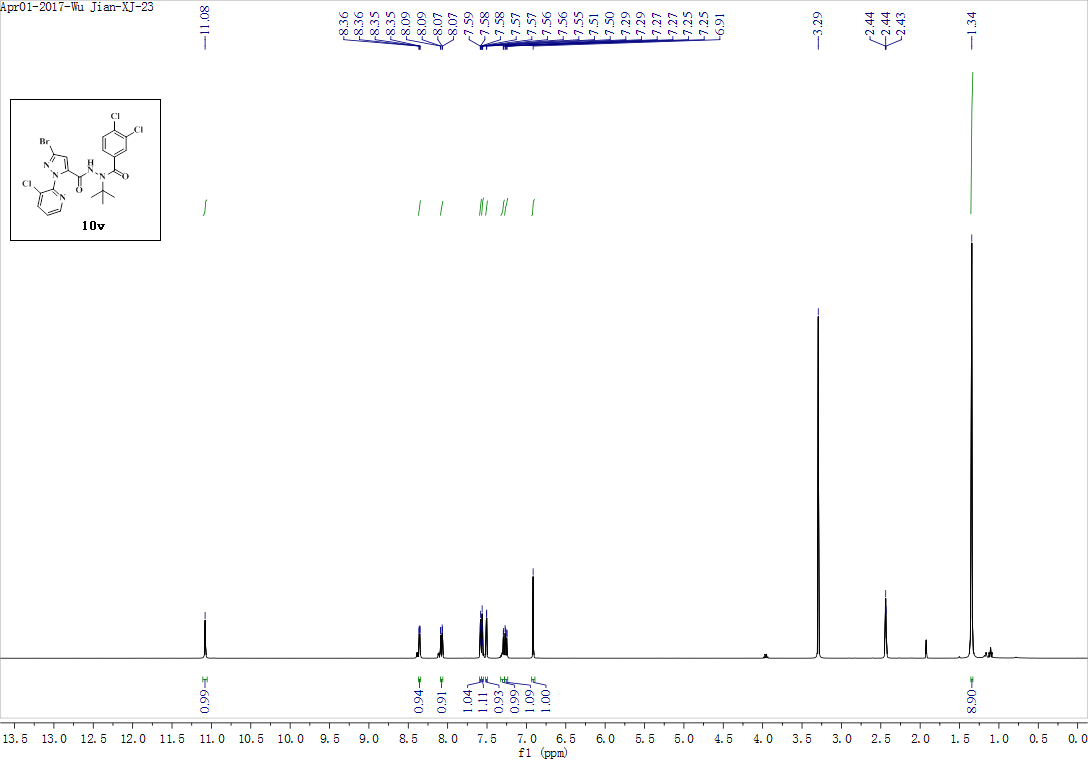


**Fig. S52** The copy of ^1^H NMR for compound **10v**


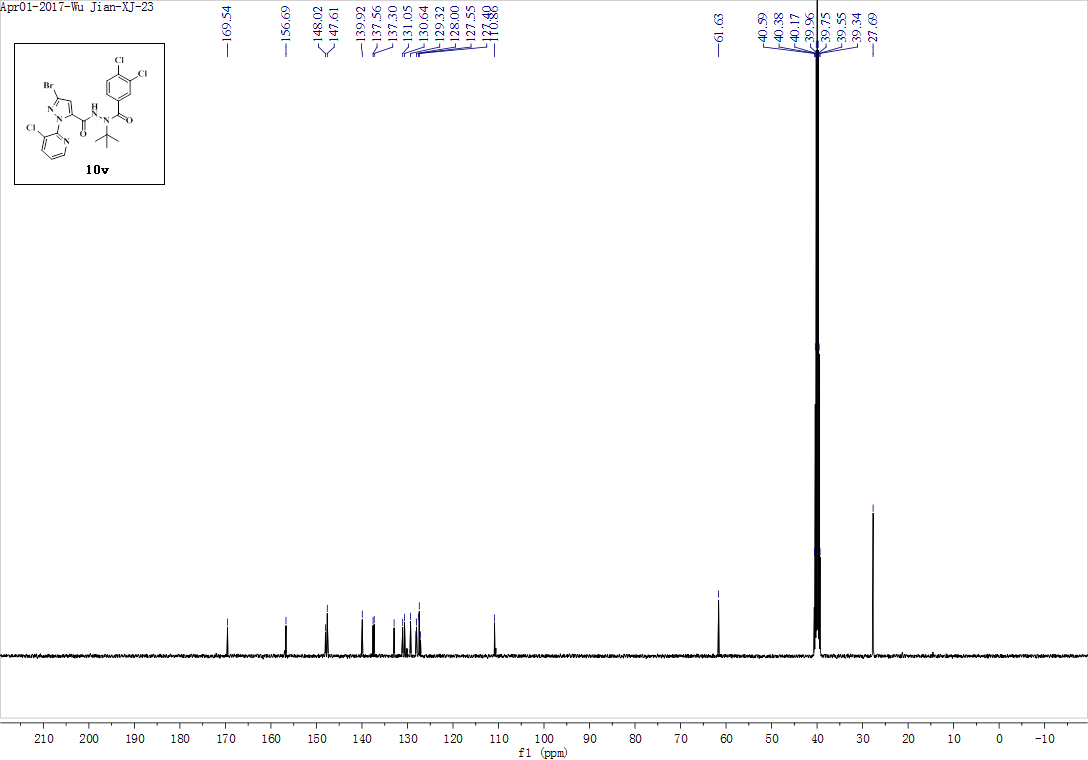


**Fig. S53** The copy of ^13^C NMR for compound **10v**


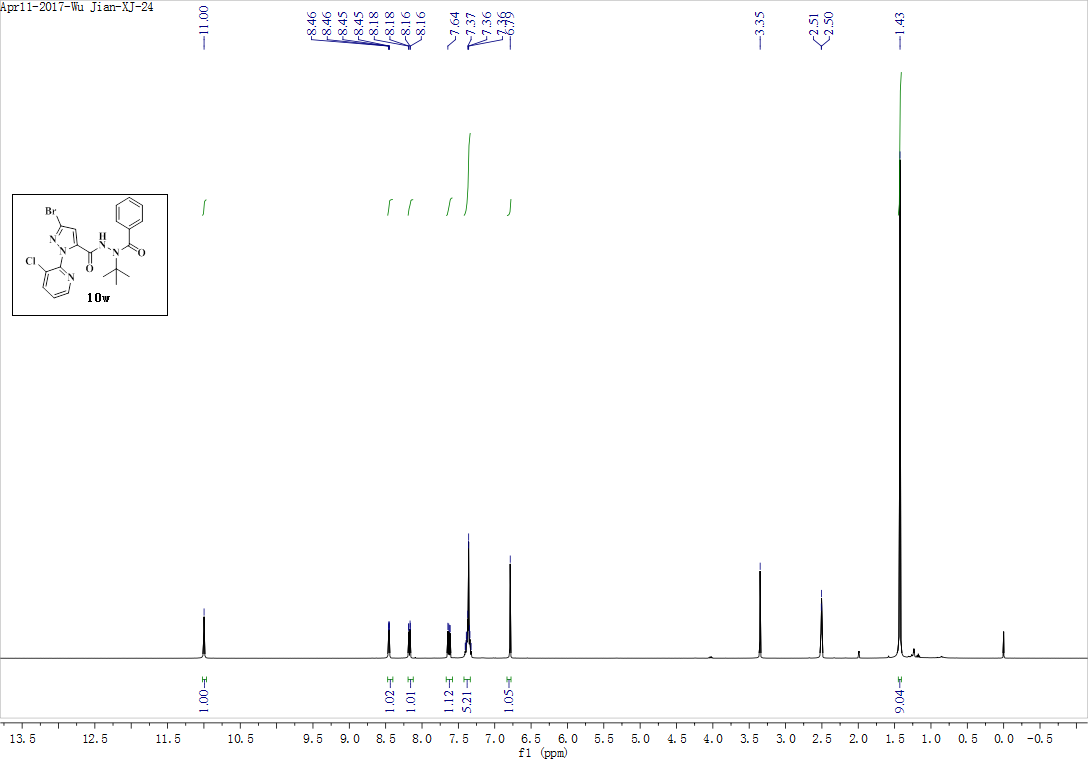


**Fig. S54** The copy of ^1^H NMR for compound **10w**


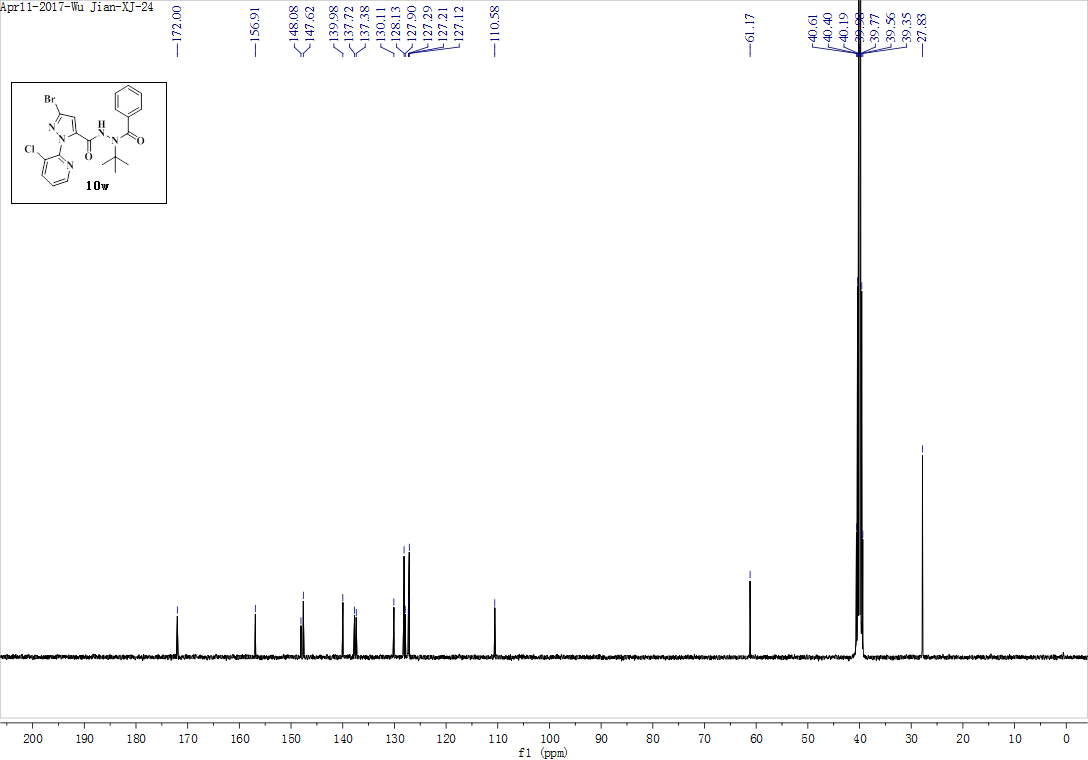


**Fig. S55** The copy of ^13^C NMR for compound **10w**


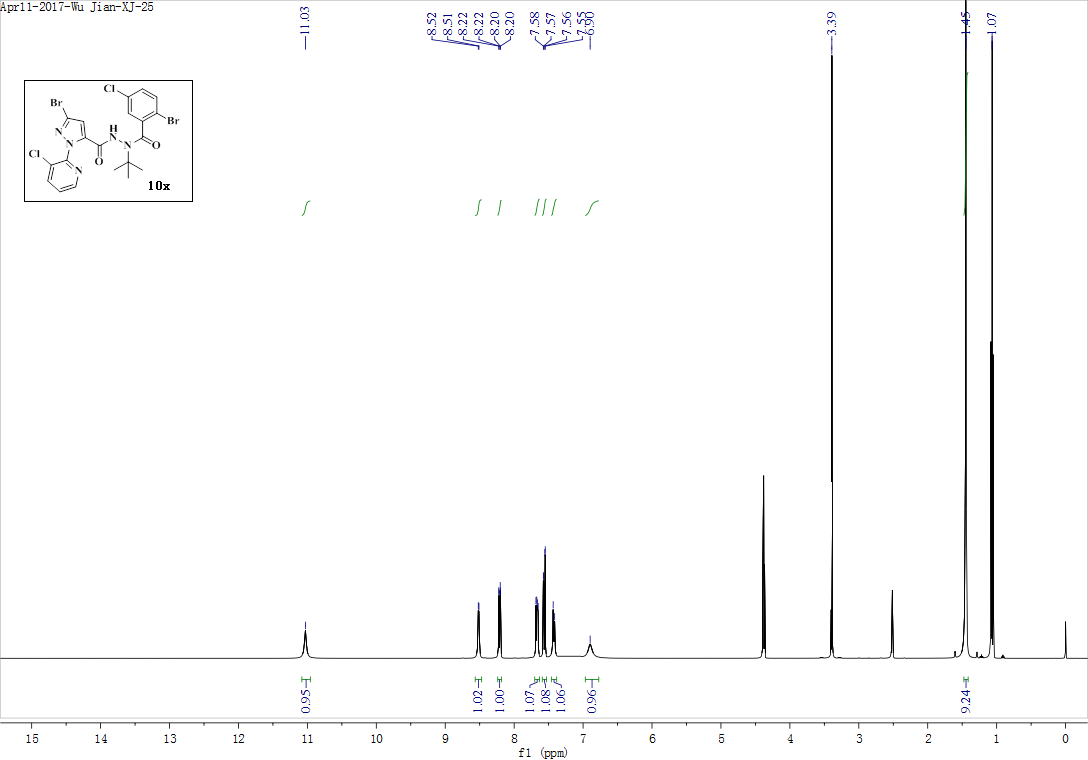


**Fig. S56** The copy of ^1^H NMR for compound **10x**


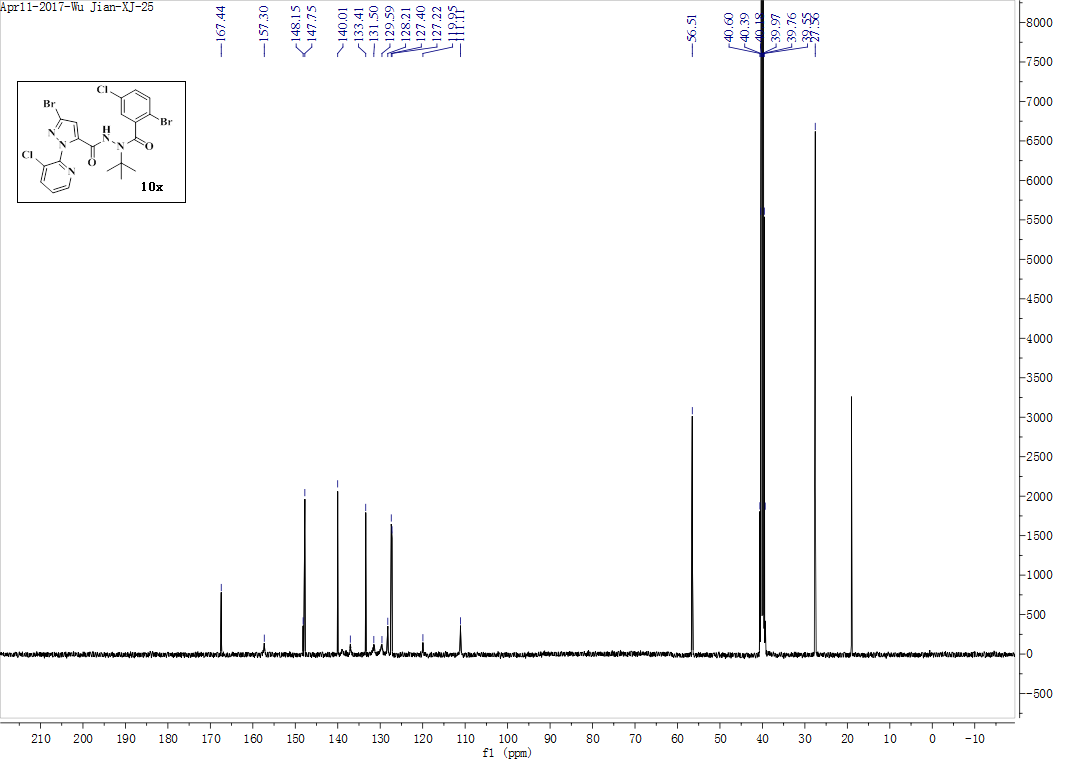


**Fig. S57** The copy of ^13^C NMR for compound **10x**
